# Evidence for exercise-based interventions across 45 different long-term conditions: an overview of systematic reviews

**DOI:** 10.1016/j.eclinm.2024.102599

**Published:** 2024-04-30

**Authors:** Grace O. Dibben, Lucy Gardiner, Hannah M.L. Young, Valerie Wells, Rachael A. Evans, Zahira Ahmed, Shaun Barber, Sarah Dean, Patrick Doherty, Nikki Gardiner, Colin Greaves, Tracy Ibbotson, Bhautesh D. Jani, Kate Jolly, Frances S. Mair, Emma McIntosh, Paula Ormandy, Sharon A. Simpson, Sayem Ahmed, Stefanie J. Krauth, Lewis Steell, Sally J. Singh, Rod S. Taylor, PERFORM research team, Samina Begum, Clara DeBarros, Firoza Davies, Kamil Sterniczuk, Rashmi Kumar, Rebecca Longley, Andrew Freeman, Jagruti Lalseta, Paul Ashby, Marc Van Grieken, Dorothy Grace Elder

**Affiliations:** a MRC/CSO Social and Public Health Sciences Unit, University of Glasgow, Glasgow, UK; b Department of Respiratory Sciences, University of Leicester, Leicester, UK; c University Hospitals of Leicester NHS Trust, Leicester, UK; d Diabetes Research Centre, University of Leicester, Leicester, UK; e Leicester Clinical Trials Unit, University of Leicester, Leicester, UK; f University of Exeter Medical School, University of Exeter, Exeter, UK; g Department of Health Sciences, University of York, York, UK; h School of Sport, Exercise and Rehabilitation Sciences, University of Birmingham, Birmingham, UK; i General Practice & Primary Care, University of Glasgow, Glasgow, UK; j Institute of Applied Health Research, University of Birmingham, Birmingham, UK; k Health Economics & Health Technology Assessment, University of Glasgow, Glasgow, UK; l School of Health and Society, University of Salford, Salford, UK; m Robertson Centre for Biostatistics, University of Glasgow, Glasgow, UK

**Keywords:** Long-term conditions, Exercise, Physical activity, Systematic review, Overview

## Abstract

**Background:**

Almost half of the global population face significant challenges from long-term conditions (LTCs) resulting in substantive health and socioeconomic burden. Exercise is a potentially key intervention in effective LTC management.

**Methods:**

In this overview of systematic reviews (SRs), we searched six electronic databases from January 2000 to October 2023 for SRs assessing health outcomes (mortality, hospitalisation, exercise capacity, disability, frailty, health-related quality of life (HRQoL), and physical activity) related to exercise-based interventions in adults (aged >18 years) diagnosed with one of 45 LTCs. Methodological quality was assessed using AMSTAR-2. International Prospective Resister of Systematic Reviews (PROSPERO) ID: CRD42022319214.

**Findings:**

Forty-two SRs plus three supplementary RCTs were included, providing 990 RCTs in 936,825 people across 39 LTCs. No evidence was identified for six LTCs. Predominant outcome domains were HRQoL (82% of SRs/RCTs) and exercise capacity (66%); whereas disability, mortality, physical activity, and hospitalisation were less frequently reported (≤25%). Evidence supporting exercise-based interventions was identified in 25 LTCs, was unclear for 13 LTCs, and for one LTC suggested no effect. No SRs considered multimorbidity in the delivery of exercise. Methodological quality varied: critically-low (33%), low (26%), moderate (26%), and high (12%).

**Interpretation:**

Exercise-based interventions improve HRQoL and exercise capacity across numerous LTCs. Key evidence gaps included limited mortality and hospitalisation data and consideration of multimorbidity impact on exercise-based interventions.

**Funding:**

This study was funded by the 10.13039/501100000272National Institute for Health and Care Research (NIHR; Personalised Exercise-Rehabilitation FOR people with Multiple long-term conditions (multimorbidity)—NIHR202020).

**Research in context**

**Evidence before this study**

Almost half of the global population suffers from at least one long-term condition (LTC) resulting in substantive health and socioeconomic burden. Exercise is a potentially key intervention in effective LTC management. Given the large number of systematic reviews of exercise-based interventions, employing an overview of reviews offers an efficient approach to consolidate evidence reported across multiple systematic reviews, to facilitate informed decision making. Preliminary searches were conducted to identify previous overviews of systematic reviews of exercise-based interventions for LTCs. Four overviews were identified which showed exercise-based interventions to be beneficial for a range of LTCs, however these overviews were limited in scope in terms of range of LTCs and health outcomes and did not consider the implications of multimorbidity.

**Added value of this study**

We provided a contemporary and comprehensive overview examining the impact of exercise-based interventions across 45 LTCs. This overview identified the value of exercise in terms of exercise capacity and HRQoL in a wide range of single index LTCs and reported on the quality of the evidence. However, there is still uncertainty about the impact of exercise for LTCs on mortality and hospitalisation. Equally our overview identified specific LTCs where the evidence for exercise is absent or less clear.

**Implications of all the available evidence**

Given the growing global burden of LTCs, healthcare systems need to urgently consider how they develop and deploy exercise interventions to better meet the needs of people living with a wider range of LTCs. Such services need to consider the impact of multiple LTCs (‘multimorbidity’) on the design and delivery of exercise interventions. Future evidence collection should focus on the effects of exercise in terms of impact on mortality and hospitalisation and provide data impacts of people with multiple LTCs.

## Introduction

Chronic disease is one the major challenges facing international healthcare systems.^1^^,^^2^ Almost half of the global population suffers from at least one long-term condition (LTC) resulting in substantive burden of premature death and morbidity, loss in health-related quality of life (HRQoL), and high socioeconomic costs.^2^, ^3^, ^4^ Defined as conditions for which there is currently no known cure,^5^ LTCs can be managed through a combination of drugs and non-pharmaceutical treatments, including exercise-based interventions (exercise training alone or in combination with others e.g., education or psychological support). Exercise-based interventions have demonstrated direct effects on both physical and mental health systems. Notably, impacts on the cardiovascular system, cognitive function, mood and mental health, metabolic health, respiratory system, and musculoskeletal system make it a potentially effective therapy for a variety of LTCs.^6^^,^^7^

Given the large number of published systematic reviews (SRs) of exercise-based interventions for LTCs, an overview of SRs provides an efficient methodology to present an overall summary of the evidence base.^8^ To date, four overviews have shown exercise-based interventions to be beneficial for a range of LTCs, reporting improvements in health outcomes including exercise capacity, HRQoL, and reductions in mortality.^9^, ^10^, ^11^, ^12^ However, there are fundamental limitations in how these previous overviews can inform how healthcare systems could best deploy exercise for people for LTCs. Notably, they focus on only a limited number of single LTCs (e.g., cardiac, pulmonary, musculoskeletal conditions), and have a narrow scope of health outcome consideration. Additionally, with increasing numbers of people living with multiple LTCs, previous studies have not formally considered the implications of co-existing LTCs (including comorbidities, i.e., presence of one or more LTC alongside a single index LTC, or multimorbidity, i.e., more than two LTCs occurring within in individual).

Therefore, the primary aim of this contemporary overview was to assess impact of exercise-based interventions in 45 different LTCs and across of a range of health outcomes (i.e., mortality, hospitalisation, exercise capacity, disability, frailty, HRQoL, and physical activity). The secondary aim was to consider the potential implications of patient multimorbidity or comorbidity.

## Methods

This study was conducted in accordance with the Cochrane guidelines for overviews of reviews,^13^ and is reported according to the Preferred Reporting Items for Overviews of Reviews (PRIOR) statement.^8^ The protocol was prospectively registered on the International Prospective Resister of Systematic Reviews (PROSPERO; ID: CRD42022319214) prior to conducting searches.

### Search strategy

A comprehensive search to 4th October 2023 was undertaken by an experienced information specialist (VW) in the electronic databases: Cochrane Database of Systematic Reviews, MEDLINE, Embase, CINAHL, and PsycINFO. A three-step sequential approach was used: (i) we first searched electronic databases using the terms “long-term condition” and “chronic disease”; (ii) for LTCs with no eligible SRs identified, we then searched electronic databases using additional LTC specific Medical Subject Headings (MeSH) terms; and (iii) for those LTCs with still no identified SR, we then performed supplementary PubMed searches using LTC descriptor terms (e.g., (anaemia OR anemia) AND exercise) for available SR or randomised controlled trial (RCT) evidence. Given the development of ‘usual medical care’ for many LTCs over the last two decades, we limited searches from the year 2000 onwards. No language restrictions were applied, and a validated filter was applied to searches i and ii to limit to SRs.^14^ Searches were first conducted in July 2022, and updated on 4th October 2023. Example search strategies are provided in Supplementary file 1.

### Eligibility criteria and SR selection

We sought SRs, published in English language within peer reviewed journals, that investigated the impact of exercise-based interventions in adults diagnosed with at least one LTC. Inclusion and exclusion criteria are detailed in Table 1. A list of 44 eligible single LTCs was determined by combining conditions identified by the Cambridge Multimorbidity Score and Barnett et al.,^1^^,^^16^ with the addition of long-COVID as an additional LTC. A full list of eligible LTCs is provided in supplementary file 2. Results of electronic database searches were deduplicated and imported into Covidence systematic review software (Veritas Health Innovation, Melbourne, Australia. Available at www.covidence.org). Two reviewers (of GOD, HY, or LG) independently conducted title and abstract screening according to inclusion and exclusion criteria. Any disagreements were resolved through discussion, or involvement of an additional reviewer (RST) if required. Full-text screening of reviews was conducted using Covidence by one reviewer (GOD) based on the inclusion and exclusion criteria. When more than one eligible SR was identified for a given LTC, the selection of a single SR followed predetermined criteria. The selected SR needed to: (i) contain RCTs; (ii) focus on a single LTC from our pre-specified list (see supplementary file 2); (iii) have the most recent and comprehensive searches; (iv) report the most outcomes of interest (see Table 1); (v) include a meta-analysis; and (vi) assess intervention reporting quality using measures such as the Template for Intervention Description and Replication (TIDieR) or Consensus on Exercise Reporting Template (CERT).^17^^,^^18^ Selection was based on consensus across reviewers (GOD, HY, LG, and RST). For some LTC categories (i.e., cancer, arthritis), we included more than one SR to reflect disease subtypes (i.e., different types of cancer, or osteo-vs. rheumatoid arthritis). Where no eligible systematic reviews were identified for a LTC, prior to concluding there is no evidence to support exercise-based interventions, we sought to include RCTs identified by our supplementary searches.

**Table 1:** Study inclusion and exclusion criteria for SRs.

Criteria	Inclusion	Exclusion
Study design	SR (defined as a literature review that includes and reports a research question, a formal search strategy, inclusion and exclusion criteria, screening methods, assessment of the quality of included studies, and provides information about data analysis and synthesis^15^) of RCTs or non-RCTs.	Narrative reviews, primary studies, case reports, case series, editorials, clinical guidelines, overviews, abstracts only.
Population	Adults (age ≥18 years) with at least one LTC diagnosis (see Supplementary Table S1).	Individuals receiving exercise training or rehabilitation as part of end-of-life care or post-transplant surgery
Intervention	Exercise-based interventions (defined as including a structured supervised or unsupervised exercise training intervention, alone or in addition to other components, delivered in any setting, including hospital, community, or home for any duration.	Prehabilitation or maintenance rehabilitation intervention. Device-based muscle training (e.g., IMT or EMS).
Comparator	No exercise control, alternative non- exercise interventions, or usual care	–
Outcomes	1 Clinical events (mortality and hospital admissions), 2 Exercise capacity (aerobic, functional or strength tests) 3 Frailty 4 Disability 5 Health-related quality of life (HRQoL), either as disease specific or generic measures 6 Physical activity levels (self-reported or device-based measurement)	No outcomes of interest reported

RCT, randomised controlled trial; LTC, long term condition; IMT, inspiratory muscle training; EMS, electrical muscle stimulation.

### Data extraction and quality appraisal

Data were extracted into a standardised, pre-piloted proforma by one reviewer (either GOD, HY, LG, or RST) and checked for accuracy by a second (either GOD, HY, LG or RST). Data were extracted on SR characteristics (i.e., search dates, number of eligible RCTs and participants); population characteristics (i.e., definitions or eligibility criteria, summary of age, sex, and diversity); intervention characteristics (i.e., intervention components, exercise details, and setting); details of comparators; outcomes for the current review; risk of bias assessments and certainty of evidence using Grading of Recommendations Assessment, Development and Evaluation (GRADE).^19^ We also extracted details regarding existence of comorbidities or multimorbidity (i.e., as an exclusion criterion or description of the prevalence amongst participants, any description of considerations, modifications or impact of co-existing LTCs on the intervention design, delivery or outcomes). For LTCs with RCT evidence only, we extracted the same details, and performed risk of bias assessment using the Cochrane Risk of bias tool,^20^ and quality of exercise intervention reporting using CERT.^18^ A single reviewer (either GOD, HY, LG or RST) applied the AMSTAR-2 (A Measurement Tool to Assess systematic Reviews) checklist to assess the methodological quality selected SRs which was checked for accuracy by a second reviewer (either GOD, HY, LG or RST). We classified the quality of the selected SRs as ‘high’, ‘moderate’, ‘low’, or ‘critically low’.^21^

### Data synthesis

As the purpose of this overview was to present and describe the current body of SR evidence,^13^ we used a data synthesis without meta-analysis (SwiM) approach, with detailed tables and graphs used to summarise and visualise the large amount of data extracted.^22^ Dichotomous outcomes (i.e., mortality and hospital admissions) are reported as risk ratios (RR) with 95% confidence interval (CI), and where not reported, we converted event data to RRs. Continuous outcomes (e.g., exercise capacity, HRQoL), are reported as mean differences (MD) and 95% CI where outcomes were reported on the same scale, or as standardized mean differences (SMD) and 95% CI for continuous outcomes reported in different units. Where subgroup results (e.g., by follow-up time, by exercise type), were reported by SRs, we selected the meta-analysis with the largest number of included participants for presentation in forest plots. Where meta-analysis was not performed within SRs we used a vote-counting approach, i.e., summing the number of statistically significant (p ≤ 0.05) results in favour of exercise intervention compared to control. Where ≥75% of outcome results within the SR for each LTC were statistically significant in favour of exercise, we concluded a ‘positive’ overall result, and where <75% of results were statistically significant in favour of exercise, we concluded ‘unclear’ overall evidence.^23^ A vote counting approach was also applied to LTCs with only RCT evidence. We checked each selected SR for potential primary study overlap and calculated the corrected covered area.^24^

### Patient and public involvement

The PERFORM (Personalised Exercise-Rehabilitation For people with Multiple long-term conditions) project Patient Advisory Group (PAG) were consulted on the design of this overview and contributed to the interpretation and presentation of the results.^25^

### Ethics

Ethical approval was not applicable for this study, as this was a secondary analysis of existing literature and data and did not involve any primary data collection from human subjects.

### Role of the funding source

The study was funded by the National Institute for Health and Care Research (NIHR; Personalised Exercise-Rehabilitation FOR people with Multiple long-term conditions (multimorbidity)—NIHR202020). The views expressed are those of the author(s) and not necessarily those of the NIHR or the Department of Health and Social Care.

## Results

### Search results

**Fig. 1:**
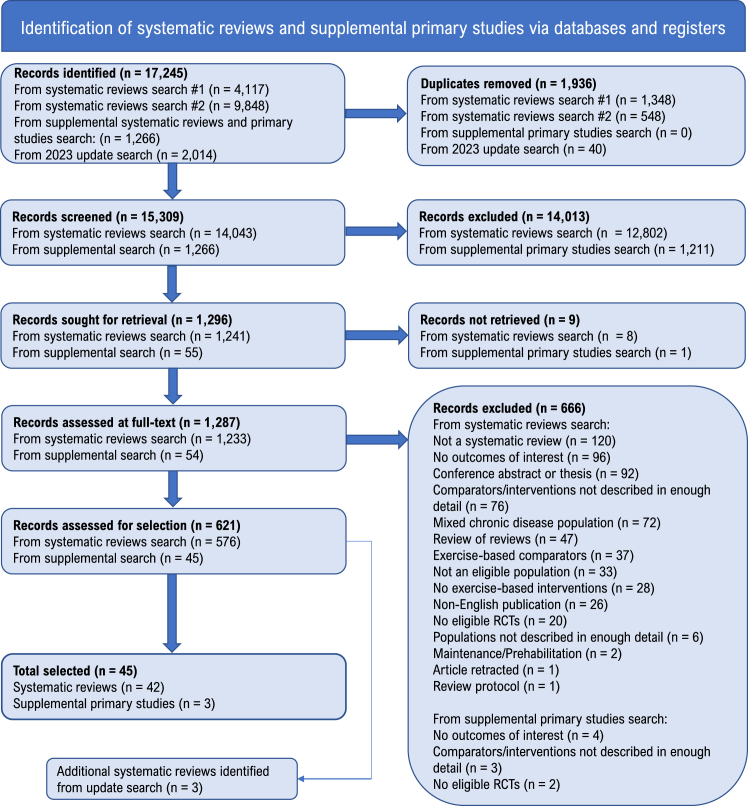
PRIOR flow diagram describing the review selection process ^a^Search #1: electronic database search using the terms “long-term condition” and “chronic disease” (conducted March 2022); ^b^Search #2: electronic database search using additional LTC specific MESH terms for LTC with no eligible SRs identified in search #1 (conducted July 2022).

Results of the search and study selection process are presented in Fig. 1. In total, 15,309 records were identified, of which 621 were eligible studies. Of these, 42 SRs were selected covering 37 LTCs,^26^, ^27^, ^28^, ^29^, ^30^, ^31^, ^32^, ^33^, ^34^, ^35^, ^36^, ^37^, ^38^, ^39^, ^40^, ^41^, ^42^, ^43^, ^44^, ^45^, ^46^, ^47^, ^48^, ^49^, ^50^, ^51^, ^52^, ^53^, ^54^, ^55^, ^56^, ^57^, ^58^, ^59^, ^60^, ^61^, ^62^, ^63^, ^64^, ^65^, ^66^, ^67^ with three LTCs having more than one SR (cancer: solid tumour, haematological and advanced metastatic; arthritis: hip osteoarthritis, knee osteoarthritis and rheumatoid arthritis; and painful condition: chronic low back pain and fibromyalgia). Two LTCs (anaemia, viral hepatitis) had no identified SRs, and instead 3 individual RCTs were identified.^68^, ^69^, ^70^ No SR or RCT evidence was identified for six LTCs (chronic sinusitis, diverticular disease, dyspepsia, Ménière's disease, psoriasis, and thyroid disease). Update searches yielded an additional 1970 records, from which a further 72 eligible SRs were identified. Following screening of these, three SRs were identified that would have met the selection criteria.^71^, ^72^, ^73^ A full list of all eligible SRs is provided in supplementary file 3. The selected evidence base included a total of 990 eligible RCTs with 936,825 individuals with a LTC (median LTC individuals per SR: 948, range 52–23,430). Seven RCTs overlapped across five of the SRs, giving a corrected covered area of 0.02% (see Supplementary file 4). As this was minimal, we did not expect the overlap to have any significant effect on the results or conclusions of this overview.^24^

### Description of evidence

The selected 42 SRs were published between 2006 and 2022, with review search dates ranging from March 2005 to November 2021. Most searches (26/42, 62%) were conducted in the last 5 years (since 2018). Thirty-six (86%) included meta-analysis. Table 2 describes the selected review characteristics. The three RCTs were published between 2008 and 2022.

**Table 2:** Characteristics of selected evidence by LTC.

LTC	Lead author (year)	Meta-analysis	Final search date	Total included studies (Eligible RCTs^b^)	N participants (N from eligible studies^b^)	Outcome follow-up duration (range)	Methodological quality assessment
Alcohol problems	Gur (2020)	Yes	July 2018	10 (5)	579 (316)	1 week to 6 months	Low
Anaemia^a^	Courneya (2008)	No	August 2022^c^	1 (1)	55	Post-intervention (1–2 weeks)	NA
Anorexia	Quiles Marcos (2021)	Yes	December 2019	10 (3)	350 (141)	Post-intervention only	Critically low
Anxiety	Stonerock (2015)	No	July 2014	12 (12)	736	NR	Low
Arthritis (osteo-, hip)	Fransen (2014)	Yes	February 2013	10 (10)	∼539 (one study NR)	Post-intervention and long-term (3–6 months)	Moderate
Arthritis (osteo-, knee)	Fransen (2015)	Yes	May 2013	54 (54)	6345	MA at immediate post-treatment, 2–6 months, >6 months	Moderate
Arthritis (rheumatoid)	Wen (2021)	Yes	August 2019	17 (13)	1010 (819)	NR	Low
Asthma	Valkenborghs (2022)	Yes	August 2021	39 (20)	2135 (933)	2 studies with 3 year follow-up	Critically low
Atrial fibrillation	Shi (2020)	Yes	December 2019	12 (12)	819	Post-intervention only	Critically low
Bronchiectasis	Lee (2017)	Yes	February 2016	4 (4)	164	Post-intervention only	Critically low
Cancer (solid tumour)	Fong (2012)	Yes	September 2011	34 (34)	3828	NR	Critically low
Cancer (haematological)	Knips (2019)	Yes	July 2018	18 (18)	1892	Range 35 days to 12 months (where reported)	Moderate
Cancer (advanced metastatic)	Chen (2020)	Yes	February 2019	15 (15)	1208	NR	Low
Chronic fatigue syndrome	Larun (2019)	Yes	May 2014	8 (7)	1518 (1404)	End of therapy (12–26 weeks) and follow up (52–70 weeks)	Moderate
Chronic kidney disease	Ibrahim (2022)	Yes	December 2020	13 (11)	619 (529)	NR	Critically low
Chronic liver disease	Aamann (2018)	Yes	February 2018	6 (6)	173	Range 8–14 weeks	Moderate
Chronic obstructive pulmonary disease	Zhang (2022)	Yes	August 2021	39 (39)	2397	Range 0.5–18 months	Critically low
Connective tissue disease	Dowman (2021)	Yes	April 2020	21 (21)	962	Range 3 weeks to 12 months	Moderate
Coronary heart disease	Dibben (2021)	Yes	September 2020	85 (85)	23,430	Median 12 months (range 6–228 months)	High
Dementia	Lam (2018)	Yes	May 2016	43 (38)	3988 (3541)	NR	Low
Depression	Schuch (2016)	Yes	August 2015	6 (6)	198	NR	Low
Diabetes mellitus	Thomas (2006)	Yes	March 2005	14 (14)	377	2 studies reported 12 month follow-up	Moderate
Endometriosis	Tennfjord (2021)	No	December 2020	3 (2)	109 (79)	Post intervention only	Low
Epilepsy	Panebianco (2015)	Yes	March 2015	2 (2)	50	6–12 months follow-up	Low
Glaucoma	Hecht (2017)	No	NR	12 (1)	1481 (90)	1 month follow-up	Critically low
Heart failure	Long (2019)	Yes	January 2018	44 (44)	5783	Median 6 months	High
Hypertension	Saredeli (2021)	Yes	August 2019	23 (23)	1952	NR	Critically low
Inflammatory bowel disease	Eckert (2019)	No	May 2018	13 (7)	603 (301)	NR	Critically low
Irritable bowel syndrome	Zhou (2019)	No	April 2018	14 (11)	683	range (where reported) 2–6 months	Critically low
Long-COVID	Fugazzaro (2022)	No	November 2021	5 (2)	512 (316)	Range 6–28 weeks	Low
Migraine	Varangot-Reille (2022)	Yes	September 2020	19 (19)	2776	Range 1 week to 8 months	Low
Multiple sclerosis	Taul-Madsen (2021)	Yes	April 2020	22 (22)	966	NR	Low
Osteoporosis	Varahra (2018)	Yes	March 2017	28 (16)	2113 (1128)	One study had 12 month follow-up (others NR)	Moderate
Painful condition (chronic back pain)	Hayden (2021)	Yes	April 2018	249 (142)	24,486 (12,872)	Median 12 weeks (IQR 8–12 weeks)	High
Painful condition (fibromyalgia)	Bidonde (2019)	Yes	December 2017	29 (23)	2088 (1675)	Range 3 weeks to 1 year	High
Parkinson's disease	Gamborg (2022)	Yes	July 2021	33 (33)	1266	NR	Critically low
Peripheral vascular disease	Lane (2017)	Yes	November 2016	32 (32)	1835	Range 2 weeks to 2 years	Moderate
Polycystic ovarian syndrome	Kite (2019)	Yes	June 2017	18 (18)	758	Post-intervention only	Moderate
Prostate disorders	Bourke (2016)	Yes	March 2015	16 (16)	1574	Range 8 weeks to 12 months	Low
Psychoactive substance misuse	Dowla (2022)	Yes	August 2021	42 (25)	2531 (2125)	NR	Critically low
Schizophrenia	Fernandez-Abscal (2021)	Yes	April 2020	57 (38)	4565 (2431)	Range 0–60 weeks	Moderate
Stroke or TIA	Saunders (2020)	Yes	July 2018	75 (75)	3617	Post-intervention to 4 years	High
Treated constipation	Gao (2019)	Yes	June 2018	9 (9)	680	Post-intervention only	Critically low
Viral hepatitis^a^	Sirisunhirun (2022) McKenna (2013)	No	August 2022^c^	2 (2)	62	Post-intervention to 1 year	NA

a RCT evidence only.

b Based on our criteria for study design (e.g. RCT), population, intervention and comparator.

c Based on our own searches.

#### LTC population demographics

The mean ages of individuals within SRs ranged widely: 18 years for schizophrenia^65^ and chronic kidney disease^39^ to 89 years for dementia.^44^ Dependent on the LTC, SRs also ranged in their sex representation i.e., all males for the prostate disorders^63^ to females for the endometriosis^47^ and polycystic ovarian syndrome.^62^ Details of diversity such as socioeconomic status or ethnicity were only reported in six SRs. Detailed descriptions of participants and eligibility criteria are presented in Supplementary Table S2.

For anaemia, the only eligible RCT identified was for people with cancer-related anaemia,^68^ and similarly for prostate disorders, the selected SR included people with prostate cancer only.^63^ The selected SR for connective tissue disease included patients with both connective tissue related, and non-connective tissue related interstitial lung disease.^41^ Fifteen SRs mentioned co-existence of LTCs among participants to some varying degree, however nine of these listed comorbidities as exclusion criteria of either the SR or included primary studies. One SR specifically reported the rate of comorbid depression amongst the included population,^38^ and one RCT specifically reported the total number of comorbidities of participants.

#### Components of exercise interventions

Training dose (in terms of exercise frequency, intensity, duration, and specific types of exercise) typically varied widely. Exercise frequency ranged from 1 session/week to several sessions/day; intensity ranged from low to maximum effort across various intensity indicators such as heart rate (HR), oxygen consumption (VO_2max/peak_), peak power output and rating of perceived exertion (RPE); duration ranged from 5 to 180 min/session; and types included cycling, walking, circuit training and water-based activities, for example. Whilst aerobic training was included across all LTCs, resistance training was also included as part of the exercise intervention across the majority of SRs (35/42, 83%). Where reported, exercise interventions within a LTC SR could include a range of differing modes and settings of delivery, e.g., supervised inpatient or outpatient hospital to unsupervised home-based exercise. None of the included SRs or RCTs provided any details of how exercise interventions may have been modified to take account of co-existing LTCs within their respective populations. Four assessments of interventions reporting quality using CERT or TIDieR were reported, with CERT scores ranging from 8 to 12 out of a total of 16, and in one SR 50% of TIDieR items were sufficiently reported. Neither CERT nor TIDieR define thresholds for ‘good’ or ‘poor’ reporting. Supplementary Table S3 provides a detailed summary of exercise intervention characteristics, and intervention reporting quality assessments (where available).

### Methodological quality of SRs

Five (12%) SRs were assessed high quality, 11 (26%) moderate quality, 12 (29%) low quality, and 14 (33%) critically low quality. Supplementary Table S4 shows the AMSTAR-2 ratings for the selected SRs. The most common critical flaws identified across the SRs were a lack of reference to protocols or PROSPERO registrations to indicate that review methods were established prior to conducting the review (14, 33%), inadequate investigation of publication bias (14, 33%), and not accounting for ROB when interpreting the SR findings (13, 31%). Common non-critical weaknesses included a lack of rationale for the selection of included study designs (41, 98%), and lack of reporting of the sources of funding of included studies (33, 79%).

### Outcome findings of SRs

**Fig. 2:**
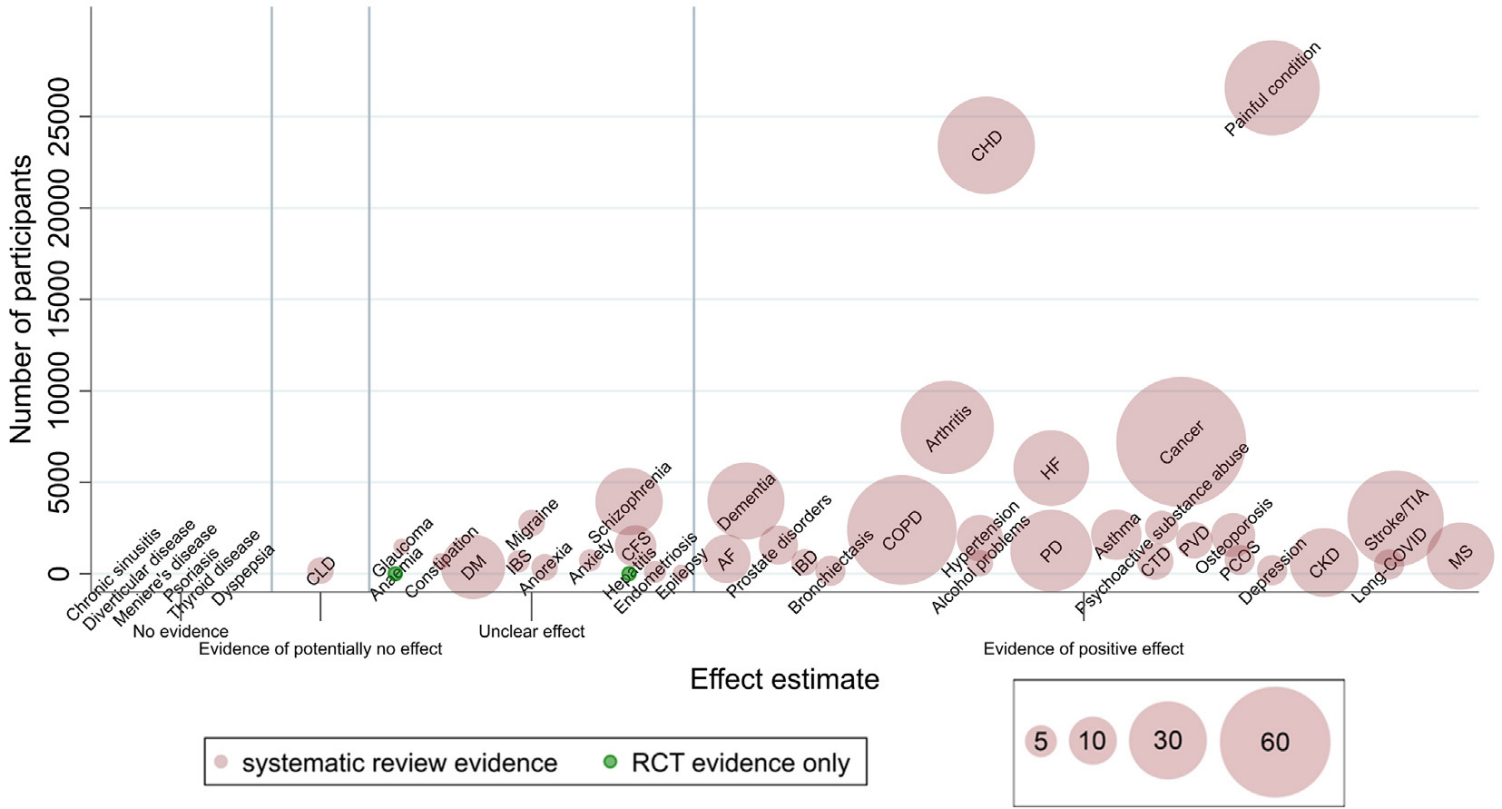
**Evidence mapping bubble plot of exercise-based interventions for long-term conditions (LTC**s). *Y-axis*: number of participants included in the selected systematic review. *X-axis*: categorisation of exercise intervention effect. • ‘No evidence’: no eligible SRs or RCTs identified • ‘Evidence of potentially no effect’: all outcomes (of interest) showed no effect + authors concluded no evidence of benefit • ‘Unclear evidence’: conflicting results for outcomes (of interest) + authors concluded unclear or insufficient evidence of benefit or all outcomes (of interest) showed no benefit, but other LTC specific outcomes showed positive effect, and authors concluded exercise is beneficial • ‘Evidence of potential positive effect’: all/most outcomes (of interest) showed positive effect and authors concluded that exercise is beneficial. • **NB- positioning within the effect estimate categories does not denote the effect size.** *Bubbles*: LTC. *Bubble size*: number of eligible SRs. *Bubble colour*: red for SR evidence; green for LTCs where only RCT evidence was identified. LTC, long-term condition; SR, systematic review; RCT, randomised controlled trial; CLD, chronic liver disease; DM, diabetes mellitus; IBS, irritable bowel syndrome; CFS, chronic fatigue syndrome; AF, atrial fibrillation; IBD, inflammatory bowel disease; COPD, chronic obstructive pulmonary disease; CHD, coronary heart disease; PD, Parkinson's disease; CTD, connective tissue disease; PVD, peripheral vascular disease; PCOS, polycystic ovarian syndrome; CKD, chronic kidney disease; TIA, transient ischaemic attack; MS, multiple sclerosis.

Based on the overall conclusions of SR authors for the reported outcomes of interest, there was ‘clear evidence’ for 25 of the 45 pre-selected LTCs (56%), unclear evidence for 13/45 (29%) LTCs, and evidence of potentially no effect for one (2%) LTC (Fig. 2 and Table 3).

**Table 3:** Overall volume of evidence, author’s conclusions, outcomes, risk of bias and overall effect of exercise-based interventions by LTC.

LTC	N SRs identified	Outcomes∗							Review authors' overall conclusions†	Risk of bias (overall description)	Overall effect
		Mortality	Hospital admission	Exercise capacity	Frailty	Disability	HRQoL	Physical activity			
Alcohol problems	3			+					+	Low	Evidence of positive effect
Anaemia	0 (RCTs only)			+			±		+	NR	Unclear
Anorexia	3			+					±	NR	Unclear
Anxiety	2			±					±	Low to medium	Unclear
Arthritis	43										Evidence of positive effect
Osteo-, hip,						+	±		+	7/10 Low	
Osteo-knee						+	+		+	20% low ROB	
Rheumatoid				+		±			+	Mean Jadad score 4	
Asthma	12			+					+	Mean PEDro score 5.5	Evidence of positive effect
Atrial fibrillation	11			+			+		+	“Limited methodological quality”	Evidence of positive effect
Bronchiectasis	4	±	±	+			+		+	NR	Evidence of positive effect
Cancer	85										Evidence of positive effect
Solid tumour				+			±		+	39% studies with unmet criteria likely to alter study conclusions	
Haematological		±		+			±		±	Unclear	
Advanced metastatic							+		+	NR	
Chronic fatigue syndrome	8						±		±	NR	Unclear
Chronic kidney disease	23			+			±		+	Mean PEDro score 5.27	Evidence of positive effect
Chronic liver disease	3	±		±			±		±	High	Evidence of potentially no effect
Chronic obstructive pulmonary disease	60			+		+	+		+	NR	Evidence of positive effect
Connective tissue disease	6	±		+			+		+	Moderate ROB in 60% studies	Evidence of positive effect
Coronary heart disease	47	±	+				+		+	NR	Evidence of positive effect
Dementia	29			+		+	±	±	+	PEDRO score: Excellent 0 Good 27 Fair 10 Poor 2	Evidence of positive effect
Depression	4						+		+	5/6 studies at higher ROB	Evidence of positive effect
Diabetes mellitus	20			+			±		+	NR	Unclear
Endometriosis	2						+		±	1 poor, 1 fair	Unclear
Epilepsy	1						±		±	NR	Unclear
Glaucoma	1						+		±	NR	Unclear
Heart failure	28	±	+				+		+	Generally low or unclear	Evidence of positive effect
Hypertension	10			+					+	PEDRO range 5–9	Evidence of positive effect
Inflammatory bowel disease	3						+		+	Rated level of evidence = 2	Evidence of positive effect
Irritable bowel syndrome	2						+		±	NR	Unclear
Long-COVID	4			+			±	+	+	1 low risk; 1 some concerns	Evidence of positive effect
Migraine	3					+	±		±	PEDRO mean score 5.3	Unclear
Multiple sclerosis	22			+					+	Median TESTEX score 9	Evidence of positive effect
Osteoporosis	9			+			+		+	Unclear or low. Mean quality 71.5%	Evidence of positive effect
Painful condition	45										Evidence of positive effect
Chronic back pain						+			+	Most judged to be at risk of bias	
Fibromyalgia				+		+	+		+	Moderate	
Parkinson’s disease	33			+			±		+	Median TESTEX score 10	Evidence of positive effect
Peripheral vascular disease	6	±		+			±		+	Moderate	Evidence of positive effect
Polycystic ovarian syndrome	4			+			±		+	NR	Evidence of positive effect
Prostate disorders	7			+			±		+	NR	Evidence of positive effect
Psychoactive substance misuse	5			±			±	+	+	Risk of bias was generally high	Evidence of positive effect
Schizophrenia	22			±		±	+	±	+	Average bias score 3.44	Unclear
Stroke or TIA	46	±		+		±	±	±	+	NR	Evidence of positive effect
Treated constipation	1						+		±	Relatively high risk of bias	Unclear
Viral hepatitis	0 (RCTs only)			±			±		±	NR	Unclear

∗ Blank cells indicate that the outcome was not reported within the SR or RCT; + = positive effect indicated by either statistically significant (p ≤ 0.05) meta-analysis of exercise compared to control, or vote counting with ≥75% statistically significant results in favour of exercise; ± = unclear or inconsistent evidence indicated by non-significant (p > 0.05) meta-analysis of exercise compared to control or vote counting with <75% statistically significant results in favour of exercise.

^†^ +: authors conclude overall that exercise is effective; ±: authors' conclude overall that evidence is unclear, inconsistent, or insufficient that exercise is effective.

The most frequently reported outcome domains across the selected SRs and RCTs were HRQoL (36/44, 82%) and exercise capacity (29/44, 66%), whereas disability (11/44, 25%), mortality (8/44, 18%), hospitalization (3/44, 7%), physical activity (5/44, 11%), and exercise intervention adherence (9/44, 20%) were less frequently reported. The outcome of frailty was not reported (Supplementary Figure S1).

#### Mortality

Mortality was reported for eight LTCs, and the number of deaths reported was generally low (see Supplementary Table S5 and Supplementary Figure S2).^34^^,^^36^^,^^40^^,^^41^^,^^43^^,^^50^^,^^61^^,^^66^ A reduction in mortality was only seen for coronary heart disease at 12–36 month (pooled RR: 0.77, 95% CI 0.63–0.93) and >36-month follow-up (pooled RR: 0.58, 95% CI 0.43–0.78) for cardiovascular related death.

#### Hospital admissions

Hospital admission data was reported for three LTCs (see Supplementary Table S6).^34^^,^^43^^,^^50^ There was evidence of a reduction in the risk of hospital admissions with exercise-based intervention for both coronary heart disease (pooled RR: 0.58, 95% CI 0.43–0.77 at 6–12 month follow-up) and heart failure (pooled RR for disease-specific hospitalisations: 0.59, 95% CI 0.42–0.84 up to 12 month follow up).

#### Exercise capacity

##### Aerobic capacity and function

Aerobic capacity and function were most consistently reported using the measures of VO_2max/peak_ or 6-min walk test (6MWT) respectively. Other aerobic capacity/function measures reported such as peak power are presented in Supplementary Table S7.

Fourteen SRs and two RCTs reported VO_2max/peak_ (Supplementary Table S7 and Supplementary Figure S3).^26^^,^^32^^,^^33^^,^^37^^,^^40^^,^^41^^,^^46^^,^^51^^,^^56^^,^^60^^,^^62^^,^^63^^,^^65^^,^^68^^,^^70^ Apart from chronic liver disease,^40^ there was consistent evidence of improvement relative to control with mean increases ranging from 0.3 to 4.9 ml/kg/min across LTCs.

A total of 12 reviews and one primary study reported 6MWT data (Supplementary Table S8 and Supplementary Figure S4).^33^^,^^37^^,^^39^, ^40^, ^41^, ^42^^,^^44^^,^^54^^,^^59^^,^^60^^,^^65^^,^^66^^,^^70^ With exception of viral hepatitis and stroke/TIA, there was significant improvement in 6MWT distance at follow-up in favour of exercise-based intervention, with mean increases ranging from 29 to 69 m.

##### Strength

Fifteen reviews and one RCT reported strength outcomes.^27^^,^^32^^,^^34^^,^^36^^,^^37^^,^^44^^,^^51^^,^^54^^,^^56^^,^^57^^,^^59^^,^^60^^,^^63^^,^^64^^,^^70^ There was consistent evidence of an improvement in strength with exercise-based intervention across 10 of the 15 LTCs (Supplementary Table S9 and Supplementary Figure S5) although effect sizes ranged from small (SMD 0.2–0.4) to large (SMD >0.8). Apart from psychoactive substance abuse,^64^ all pooled strength results were based on majority exercise programmes that consisted of either resistance training alone, or mixed exercise which incorporated some resistance training.

#### Disability

Eight LTCs reported disability using a range of disease-specific outcome measures, including the Health Assessment Questionnaire (HAQ) and Oswestry Disability scale (Supplementary Table S10).^29^, ^30^, ^31^^,^^42^^,^^44^^,^^55^^,^^58^, ^59^, ^60^^,^^65^^,^^66^ There was consistent evidence of benefit following exercise-based intervention across seven LTCs, with effect sizes ranging from small (SMD 0.1–0.37) to medium (SMD 0.52–0.57).

#### HRQoL

HRQoL was reported for 32 LTCs using a wide range of measures that included 27 different named HRQoL questionnaires—17 were disease specific measures (Supplementary Table S11)^34^^,^^37^^,^^39^, ^40^, ^41^, ^42^^,^^47^^,^^49^^,^^50^^,^^52^^,^^53^^,^^55^^,^^59^^,^^60^^,^^63^^,^^64^^,^^68^^,^^69^ and eight generic measures Supplementary Table S12, Supplementary Figures S6 and S7).^29^^,^^30^^,^^33^^,^^35^, ^36^, ^37^, ^38^, ^39^, ^40^^,^^43^, ^44^, ^45^, ^46^^,^^48^^,^^50^^,^^52^, ^53^, ^54^, ^55^^,^^57^^,^^60^, ^61^, ^62^^,^^65^, ^66^, ^67^^,^^70^

Improvements in both disease specific and generic HRQoL were found for three LTCs,^50^^,^^52^^,^^53^ there were improvements in disease specific HRQoL for eight LTCs^34^^,^^39^^,^^41^^,^^42^^,^^47^^,^^49^^,^^59^^,^^60^ and improvements in generic HRQoL for a further eight LTCs.^33^^,^^43^^,^^45^^,^^55^^,^^57^^,^^61^^,^^65^^,^^67^ For 13 LTCs there was no evidence of difference in either generic or disease specific HRQoL.^29^^,^^30^^,^^35^, ^36^, ^37^, ^38^^,^^40^^,^^44^^,^^46^^,^^48^^,^^54^^,^^62^, ^63^, ^64^^,^^66^^,^^68^, ^69^, ^70^

#### Physical activity

Physical activity data was reported for five LTCs (Supplementary Table S13)^44^^,^^54^^,^^64^, ^65^, ^66^ and measured using a variety of self-reported and objective methods. Long-COVID and psychoactive substance abuse were the only LTCs with evidence of increased physical activity with exercise-based intervention.

#### Exercise adherence

Seven SRs and two RCTs reported adherence to the exercise interventions.^34^^,^^44^^,^^51^^,^^57^^,^^58^^,^^60^^,^^66^^,^^68^^,^^69^ Adherence was summarized in terms of session attendance (ranging 33–100% across seven LTCs), achieving prescribed exercise intensity or dose (ranging 70–94.7% across two LTCs), or compliance (75%–99% across three LTCs).

## Discussion

This overview builds upon previous studies and summarises the evidence from 42 SRs (36 meta-analyses) and three supplementary RCTs, providing a total of 990 RCTs in 936,825 people across 39 different LTCs. We found that participation in exercise was beneficial in 25 out of the 45 pre-specified single LTCs, with consistent improvements in exercise capacity and HRQoL compared to no exercise control. However, the quality of evidence was mixed. Three main limitations identified across the included SRs were: the lack of an explicit statement that review methods were established prior to the conduct of the review, limited provision of a rationale for the selection of included study designs, and lack of reporting of sources of funding. It is important to note that these limitations may reflect poor reporting rather than their poor methodological quality per se.

Our overview identified limited reporting of key outcomes across LTCs including mortality and hospital admissions, disability, frailty, and physical activity. This paucity of data limits our ability to fully understand the comprehensive impact of exercise-based interventions on important aspects of health. Moreover, these later outcomes have recently been identified as core outcome measures for exercise and rehabilitation.^74^^,^^75^ Despite exercise being considered a universally effective intervention, evidence for the impact of exercise was lacking in seven out 45 LTCs and evidence was uncertain for 13 LTCs. Whilst it was a specific objective of this overview, none of the included SRs or RCTs provided information on consideration of multimorbidity in either the design and delivery of the exercise intervention, nor on its impact on the effectiveness of exercise. In contrast, the presence of other LTCs was often used as exclusion criteria by primary studies.

Our study has several strengths. Our review scope is much wider than that of previous overviews of exercise for chronic conditions that considered fewer LTCs and often only considered the outcome of exercise capacity.^9^, ^10^, ^11^, ^12^ A multistage approach to SR selection was employed to maximise the contemporariness as well as the likelihood of the quality and relevance of the SR evidence. In addition, we conducted and report this overview in accordance with current guidance,^8^^,^^13^ and we extracted TiDER and CERT assessments of the quality of intervention reporting.^17^^,^^18^ Where no SRs were found for an individual LTC, we undertook additional literature searches to seek individual RCTs prior to concluding there was no evidence for the LTC.

Despite this, it is important to acknowledge the limitations of our study. Firstly, we did not include all LTCs. However, our scope of included LTCs was informed by epidemiological evidence, and we also updated our list to include long-COVID.^1^^,^^16^ We recognise that we may have included some LTCs where the biological plausibility of benefit for exercise may be low (e.g., psoriasis). Secondly, our selection of SRs was focused on the pre-selected single LTCs, and maximising comprehensiveness, recency, consideration of relevant outcomes and their reporting in a meta-analysis. However, these criteria may have resulted in the selection of lower quality SRs at the expense of a higher quality review, potentially compromising the reliability of their findings. Thirdly, we acknowledge the rapidly evolving nature of evidence for exercise-based rehabilitation. Our updated searches identified a further three SRs, that could have been included in this overview,^71^, ^72^, ^73^ however, only one of these SRs would have changed our conclusion (i.e., to unclear evidence for IBD). Also, we are aware of a recently published SR reporting that exercise improves HRQoL for people with Type 2 diabetes that was not identified by our literature searches.^76^ Finally, we acknowledge that initial full-text screening was performed by a single reviewer, and we excluded SRs that were not published in English, which may have introduced language bias.

Given the inconsistent assessment of publication bias across the selected SRs, the impact of this potential bias remains unclear. However, for some included reviews this was the case due to insufficient RCTs with relevant outcome data to test for funnel plot asymmetry (i.e., ≤10 studies).^77^ In our protocol we stated that we aimed to explore differences in effect based on delivery setting, but as this was inconsistently reported across selected reviews, this subgroup comparison was not performed. Poor reporting of ethnicity and socio-economic status also limits our ability to examine the potential for greater health inequalities. Finally, although there exists an internationally accepted framework for developing and presenting summaries of evidence, which provides a systematic approach for making clinical practice recommendations,^19^ only 15 (36%) SRs in this overview employed GRADE.

This overview has important implications for current policy and future research. First and foremost, our findings demonstrate the need for health systems to widen their access to exercise-based interventions to include a range of LTCs. In the UK and other developed economies, access to exercise-based services is currently limited to a small group LTCs; for example, commissioned cardiac and pulmonary rehabilitation services that target exercise referral to those with a diagnosis of coronary heart disease, heart failure or chronic obstructive disease.^78^^,^^79^ The 2019 Global Burden of Disease report estimated some 2.4 billion individuals globally have conditions that would benefit from rehabilitation (including exercise), contributing to 310 million years of life lived with disability.^80^ Such future provision should include the 25 LTCs identified in this review. Second, most SRs were of low or critically low quality, therefore there is a need for improved methodological rigour and reporting of future SRs. In addition, adherence to frameworks for reporting intervention details^17^^,^^18^ would enhance the comparability of studies across LTCs, given the heterogeneity and broadness of ‘exercise’ as an intervention. Policymakers must also recognise the diversity within this overarching intervention and within LTC populations and acknowledge that a one-size-fits-all approach may not be applicable.

Third, since none of the SRs in this overview considered how exercise interventions take account of the specific needs of people with multiple LTCs, there remains a lack of clarity of how best to design and deliver exercise services for such people. Given the rising prevalence and substantive negative health burden of multimorbidity, this is a key area for future direction. A number of commentators have called for health systems to revamp their exercise-based services with a multimorbidity focus.^81^, ^82^, ^83^ There is emerging evidence supporting the feasibility of exercise programmes for multiple LTCs.^84^^,^^85^ An ongoing example is the PERFORM research programme funded by the UK National Institute for Health Research (NIHR) aimed at developing and evaluating an exercise-based service specifically designed to meet the needs of people with multiple LTCs.^25^ The findings of this overview have directly informed the inclusion criteria of the ongoing PERFORM pilot RCT.^25^ Considerations for the future evidence collection for exercise and LTCs are highlighted in Box 1.

Box 1 **Considerations for future evidence collection of exercise interventions for people with LTCs.**

• A focus on LTCs identified in this overview with no SR or RCT evidence.
• Improve methodological rigour and reporting of SRs according to PRISMA guidelines.
• Improve reporting of details of exercise intervention delivery (e.g., dose, providers, setting) and individual levels of participation/adherence to exercise programmes. Use of TiDeR and CERT reporting checklists.^14^^,^^16^
• Reporting of the impact of exercise interventions across a range of outcomes that include exercise capacity, HRQoL, mortality, hospital admissions, disability, physical activity.
• Consideration of the importance of multiple LTCs in terms of both the design and delivery of exercise interventions and their impact on outcomes.

In conclusion, we found evidence that participation in exercise-based interventions was beneficial in 25 out of the 45 pre-specified LTCs, supported by improvements in HRQoL and exercise capacity. Key evidence gaps included limited mortality and hospitalisation data and consideration of the potential impact of multimorbidity on delivery of exercise-based interventions. We also identified a need for improved methodological rigour and reporting in future SRs, and identified specific LTCs where the evidence for exercise is absent or less clear. In response to the growing global burden of LTCs, healthcare systems must urgently consider the development and implementation of exercise interventions to better address the needs of people living with a broader spectrum of LTCs. Such services need to consider the impact of multiple LTCs (‘multimorbidity’) on the design and delivery of exercise interventions.

## Contributors

GD, BDJ, FM, EM, SS, and RST conceived the study. GOD, RST and SS designed the review protocol. VW developed search strategy and ran database searches. GOD, LG, HY and RST performed screening, study selection, data extraction and quality appraisal. GOD synthesized the data. GOD and RST interpreted the data. GOD and RST drafted the manuscript. All co-authors revised drafts of the manuscript and approved the final version. GOD and RST accessed and verified that data, take final responsibility for the paper, and act as guarantors. All co-authors read and approved final manuscript.

## Data sharing statement

Data collected for the study will be made available on request to the corresponding author.

## Declaration of interests

GOD is co-author of one, and RST is co-author of two of the SRs included in this overview. LG is currently in receipt of/undertaking a Wellcome Trust doctoral fellowship (UNS144807) and declares receipt of payment for lecture on pulmonary rehabilitation (University College London, annual), Council of Allied Health Professions Research (CAHPR)/National Institute for Health and Care Research (NIHR) Research Champion: West Midlands (unpaid), British Thoracic Society (BTS): Pulmonary Rehabilitation (PR) Specialist Advisory Group (SAG) member (unpaid), Association of Chartered Physiotherapists in Respiratory Care (ACPRC) committee (honoraria received). HMLY is funded by the NIHR Advanced Fellowship (NIHR202926). SJS is Clinical Lead for National Respiratory Audit Programme—Pulmonary Rehabilitation. KJ declares funding from NIHR Applied Research Collaboration West Midlands and Sub-committee chair for NIHR Programme Grants for Applied Health Research (payment to institution). RAE declares receipt of speaker fees (Boeringher June 2021; Moderna April 2023) and ERS Group 01.02 Pulmonary Rehabilitation and Chronic Care Secretary (unpaid), and ATS Pulmonary Rehabilitation Assembly Chair (unpaid). SD declares NIHR Applied Research Collaboration: South West Peninsula (PenARC; payment to institution), receipt of the following NIHR grants (payment to institution): NIHR151938; NIHR204099; RP-PG-0514-20002; NIHR201038; NIHR201070; NIHR200428, receipt of grants (payment to institution): Gillings Family foundation (ID 943008); The Stroke Association (ID 901902); NIHR School for Primary Care Research—Exeter internal fund (ID 856766); Academic Health Science Network South West (ID 1355693), receipt of textbook royalties (John Wiley & Sons), support for meeting attendance from NIHR (p-PG-0514-20002) and Health Research Council New Zealand (21/826; 18/254), and membership of NIHR Programme Grant for Applied Research funding panel committee and The Stroke Association research funding panel. SJK declares receipt of conference funding from School of Health and Wellbeing, University of Glasgow. SAS declares presidency of the UK Society of Behavioural Medicine, membership of HTA Clinical Evaluations and Trials Committee (2016–2020), membership of Commissioning Panel for the National Institute of Health Research (NIHR) Policy Research Programme (2019–2022), and membership of Chief Scientist Office HIPS committee (2018–2023).
