# Supplementary material for: Evidence for exercise-based interventions across 45 different long-term conditions: an overview of systematic reviews
Source: eClinicalMedicine. 2024 Apr 30;72:102599. doi: 10.1016/j.eclinm.2024.102599 (PMC11247153; doi:10.1016/j.eclinm.2024.102599)
Supplement: Supplementary files [file mmc1.pdf]

Exercise-based interventions for people with long term conditions: an overview of systematic reviews

Supplementary files

Contents

1. Search strategy 3

2. Supplementary Table 1 - List of included LTCs adapted from the Cambridge Multimorbidity Score and Barnett 2012 5

3. List of eligible but not selected reviews 6

4. Primary study overlap 31

5. Supplementary Table 2 – Characteristics of LTC participants across selected SRs and RCTs as reported by authors 32

6. Supplementary Table 3 – Characteristics of exercise interventions of selected SRs and RCTs as reported by authors 38

7. Supplementary Table 4 – AMSTAR assessments for selected SRs 44

8. Supplementary Figure 1 – Frequency of outcome reporting across included studies 45

9. Supplementary Table 5 – Summary of exercise-based intervention vs control mortality by LTC 46

10. Supplementary Figure 2 – mortality forest plot comparing exercise-based intervention vs control 48

11. Supplementary Table 6 – Summary of exercise-based intervention vs control for hospital admissions by LTC 49

12. Supplementary Table 7 – Summary of exercise-based intervention vs control for aerobic exercise capacity outcomes by LTC 50

13. Supplementary Figure 3 – Forest plot showing meta-analysis results for VO2max/peak. Panel A shows results reported as ml/min/kg with mean differences and as standardised mean differences on panel B. 52

14. Supplementary Table 8 – Summary of exercise-based intervention vs control for functional exercise capacity outcomes by LTC 53

15. Supplementary Figure 4 – Forest plot showing meta-analysis results for 6MWT (m). 57

16. Supplementary Table 9 – Summary of exercise-based intervention vs control for strength outcomes by LTC 58

17. Supplementary Figure 5 – Forest plot showing meta-analysis results for strength (standardised mean difference). 61

18. Supplementary Table 10 – Summary of exercise-based intervention vs control for disability outcomes by LTC 62

19. Supplementary Table 11 – Summary of exercise-based intervention vs control for disease specific HRQoL outcomes by LTC 65

20. Supplementary Table 12 – Summary of exercise-based intervention vs control for generic HRQoL outcomes by LTC 68

21. Supplementary Figure 6 – Forest plot for generic HRQoL for exercise-based intervention vs control across LTCs – standardized mean difference scale. 74

22. Supplementary Figure 7 – Forest plot for HRQoL for exercise-based intervention vs control measured using three different SF-36 domains. 75

23. Supplementary Table 13 – Summary of exercise-based intervention vs control for physical activity outcomes by LTC 76

1. Search strategy

Search 1

Medline search 23rd March 2022

Ovid MEDLINE(R)

1	(LONG* TERM CONDITION* or CHRONIC DISEAS* or MULTIMORBIDITY or CHRONIC CONDITION* or LONG* TERM ILLNESS* or CHRONIC ILLNESS*).ab,kw,ti.
2	(long adj3 (covid* or ncov* or novel coronavirus or novel betacoronavirus or sars-ncov-2 or sars-cov-2)).mp.
3	((chronic* or longterm or long term or long-term) adj3 (post covid or post-covid or postcovid)).mp.
4	(covid adj2 (consequence* or impact or post-acute or chronic or prolonged or persist*)).ab,kw,ti.
5	Chronic Disease/
6	Rehabilitation/ or Exercise/ or Exercise therapy/
7	(rehabilit* or exercise*).ab,kw,ti.
8	(exercise adj therapy).ab,kw,ti.
9	(physical* adj (fit* or train* or therap* or activ*)).ab,kw,ti.
10	(train* adj (strength* or resistance or aerobic* or exercise*)).ab,kw,ti.
11	((exercise* or fitness) adj3 (treatment or intervent* or program*)).ab,kw,ti.
12	((lifestyle or life style) adj5 (interven* or program* or treatment*)).ab,kw,ti.
13	((behavior* or behaviour*) adj5 (modify or modificat* or therap* or change)).ab,kw,ti.
14	Physical Fitness/
15	(fitness or capacity or function*).ab,kw,ti.
16	(oxygen consumption or oxygen uptake).ab,kw,ti.
17	Oxygen consumption/
18	(VO2 adj (peak or max)).ab,kw,ti.
19	(physical* adj (fit* or train* or therap* or activ*)).ab,kw,ti.
20	"six min* walk* test".ab,kw,ti.
21	(incremental shuttle walk or ISWT).ab,kw,ti.
22	Physical Endurance/
23	(endurance or speed or distance).ab,kw,ti.
24	"Activities of Daily Living"/
25	(activities of daily living or ADL).ab,kw,ti.
26	Muscle Strength/
27	(strength or force or power).ab,kw,ti.
28	(Balance or frailty or mobility or gait or falls).ab,kw,ti.
29	"Quality of Life"/
30	(quality of life or QOL or HRQOL or symptom* or burden or qaly or short form* or SF12 or SF36 or euroqol or eq5d).ab,kw,ti.
31	Mortality/
32	(mortality or Hospital* or admission or inpatient or Disability or activity limitation or Physical activ* or pedometer or accelerometer or IPAQ or steps or energy expenditure or EE).ab,kw,ti.
33	1 or 2 or 3 or 4 or 5
34	14 or 15 or 16 or 17 or 18 or 19 or 20 or 21 or 22 or 23 or 24 or 25 or 26 or 27 or 28 or 29 or 30 or 31 or 32 or 33
35	6 or 7 or 8 or 9 or 10 or 11 or 12 or 13
36	33 and 34 and 35
37	MEDLINE.tw.
38	systematic review.tw.
39	meta-analysis.pt.
40	intervention$.ti.
41	37 or 38 or 39 or 40
42	36 and 41
43	randomized controlled trial.pt.
44	42 not 43
45	limit 44 to yr="2000 -Current"

Search 2

Medline search 1st June 2022
Ovid MEDLINE(R)

1	Alcohol-Related Disorders/ or Fatigue Syndrome, Chronic/ or Depression/ or Bulimia/ or Anorexia Nervosa/ or Irritable Bowel Syndrome/ or Peripheral Vascular Diseases/ or Thyroid Diseases/ or Anxiety/ or Liver Diseases/ or Diverticular Diseases/ or Anemia, Pernicious/ or Constipation/ or Meniere Disease/ or Polycystic Ovary Syndrome/ or Dyspepsia/ or Sinusitis/ or Epilepsy/ or Migraine Disorders/ or Prostatic Diseases/ or Hepatitis, Viral, Human/ or Atrial Fibrillation/ or Glaucoma/ or Eczema/ or Coronary Disease/ or Osteoporosis/ or Substance-Related Disorders/ or Dementia/ or Hypertension/ or Schizophrenia/ or Bipolar Disorder/
2	Rehabilitation/ or Exercise/ or Exercise therapy/
3	(rehabilit* or exercise*).ab,kw,ti.
4	(exercise adj therapy).ab,kw,ti.
5	(physical* adj (fit* or train* or therap* or activ*)).ab,kw,ti.
6	(train* adj (strength* or resistance or aerobic* or exercise*)).ab,kw,ti.
7	((exercise* or fitness) adj3 (treatment or intervent* or program*)).ab,kw,ti.
8	((lifestyle or life style) adj5 (interven* or program* or treatment*)).ab,kw,ti.
9	((behavior* or behaviour*) adj5 (modify or modificat* or therap* or change)).ab,kw,ti.
10	Physical Fitness/
11	(fitness or capacity or function*).ab,kw,ti.
12	(oxygen adj consumption).ab,kw,ti.
13	(oxygen adj uptake).ab,kw,ti.
14	Oxygen consumption/
15	(VO2 adj (peak or max)).ab,kw,ti.
16	(physical* adj (fit* or train* or therap* or activ*)).ab,kw,ti.
17	"six min* walk* test".ab,kw,ti.
18	(incremental shuttle walk or ISWT).ab,kw,ti.
19	Physical Endurance/
20	(endurance or speed or distance).ab,kw,ti.
21	"Activities of Daily Living"/
22	(activities of daily living or ADL).ab,kw,ti.
23	Muscle Strength/
24	(strength or force or power).ab,kw,ti.
25	(Balance or frailty or mobility or gait or falls).ab,kw,ti.
26	"Quality of Life"/
27	(quality of life or QOL or HRQOL or symptom* or burden or qaly or short form* or SF12 or SF36 or euroqol or eq5d).ab,kw,ti.
28	Mortality/
29	(mortality or Hospital* or admission or inpatient or Disability or activity limitation or Physical activ* or pedometer or accelerometer or IPAQ or steps or energy expenditure or EE).ab,kw,ti.
30	10 or 11 or 12 or 13 or 14 or 15 or 16 or 17 or 18 or 19 or 20 or 21 or 22 or 23 or 24 or 25 or 26 or 27 or 28 or 29
31	2 or 3 or 4 or 5 or 6 or 7 or 8 or 9
32	MEDLINE.tw.
33	systematic review.tw.
34	meta-analysis.pt.
35	intervention$.ti.
36	32 or 33 or 34 or 35
37	randomized controlled trial.pt.
38	36 not 37
39	1 and 30 and 31
40	38 and 39
41	limit 40 to yr="2000 -Current"

2. Supplementary Table 1 - List of included LTCs adapted from the Cambridge Multimorbidity Score and Barnett 2012

Alcohol problems	Chronic fatigue syndrome	Depression	Inflammatory bowel disease	Parkinson's disease	Stroke or transient ischaemic attack (TIA)
Anorexia nervosa or bulimia	Chronic kidney disease	Diabetes mellitus	Irritable bowel syndrome	Peripheral vascular disease	Thyroid disease
Anxiety	Chronic liver disease	Diverticular disease	Long-COVID	Pernicious anaemia	Treated constipation
Arthritis	Chronic obstructive pulmonary disease (COPD)	Endometriosis	Meniere's disease	Polycystic ovarian syndrome	Treated dyspepsia
Asthma	Chronic sinusitis	Epilepsy	Migraine	Prostate disorders	Viral hepatitis
Atrial fibrillation	Connective tissue disease	Glaucoma	Multiple sclerosis	Psoriasis or eczema	
Bronchiectasis	Coronary heart disease	Heart failure	Osteoporosis	Psychoactive substance misuse	
Cancer	Dementia	Hypertension	Painful condition	Schizophrenia or bipolar affective disorder	

3. List of eligible but not selected reviews

Abbott H. Exercise therapy is effective for chronic low back pain, but should be individually prescribed and supervised. New Zealand Journal of Physiotherapy. 2005;33(3):115-6.

Abdin S, Lavallee JF, Faulkner J, Husted M. A systematic review of the effectiveness of physical activity interventions in adults with breast cancer by physical activity type and mode of participation. Psycho-oncology. 2019;28(7):1381-93.

Abdul-Aziz AA, Altawil M, Lyon A, MacEachern M, Richardson CR, Rubenfire M, et al. Lifestyle Therapy for the Management of Atrial Fibrillation. The American journal of cardiology. 2018;121(9):1112-7.

Abell B, Glasziou P, Hoffmann T. The Contribution of Individual Exercise Training Components to Clinical Outcomes in Randomised Controlled Trials of Cardiac Rehabilitation: A Systematic Review and Meta-regression. Sports Med Open. 2017;3(1):19.

Abraham LN, Sibilitz KL, Berg SK, Tang LH, Risom SS, Lindschou J, et al. Exercise-based cardiac rehabilitation for adults after heart valve surgery. Cochrane Database of Systematic Reviews. 2021(5).

Ada L, Dorsch S, Canning CG. Strengthening interventions increase strength and improve activity after stroke: a systematic review. Aust J Physiother. 2006;52(4):241-8.

Adeniyi FB, Young T. Weight loss interventions for chronic asthma. Cochrane Database of Systematic Reviews. 2012(7).

Afkar A, Ashouri A, Rahmani M, Emami Sigaroudi A. Effect of exercise therapy on quality of life of patients with multiple sclerosis in Iran: a systematic review and meta-analysis. Neurological Sciences. 2017;38(11):1901-11.

Afsar B, Siriopol D, Aslan G, Eren OC, Dagel T, Kilic U, et al. The impact of exercise on physical function, cardiovascular outcomes and quality of life in chronic kidney disease patients: a systematic review. International urology and nephrology. 2018;50(5):885-904.

Ahmed SA, Mewes JC, Vrijhoef H. Assessment of the scientific rigour of randomized controlled trials on the effectiveness of cognitive behavioural therapy and graded exercise therapy for patients with myalgic encephalomyelitis/chronic fatigue syndrome: A systematic review. Journal of health psychology. 2020;25(2):240-55.

Aladro-Gonzalvo AR, Araya-Vargas GA, Machado-Diaz M, Salazar-Rojas W. Pilates-based exercise for persistent, non-specific low back pain and associated functional disability: a meta-analysis with meta-regression. Journal of bodywork and movement therapies. 2013;17(1):125-36.

Albanaqi AL, Rahimi GRM, Smart NA. Exercise Training for Pulmonary Hypertension: A Systematic Review and Meta-Analysis of Randomized Controlled Trials. Biological research for nursing. 2021;23(3):442-54.

Alharbi MG, Kalra HS, Suri M, Soni N, Okpaleke N, Yadav S, et al. Pulmonary Rehabilitation in Management of Chronic Obstructive Pulmonary Disease. Cureus. 2021;13(10):e18414.

Ali N, Tian H, Thabane L, Ma J, Wu H, Zhong Q, et al. The Effects of Dual-Task Training on Cognitive and Physical Functions in Older Adults with Cognitive Impairment; A Systematic Review and Meta-Analysis. Journal of Prevention of Alzheimer's Disease. 2022;9(2):359-70.

Alves Da Rocha P, McClelland J, Morris ME. Complementary physical therapies for movement disorders in Parkinson's disease: a systematic review. European journal of physical and rehabilitation medicine. 2015;51(6):693-704.

Amaral LKB, Souza MB, Campos MGM, Mendonca VA, Bastone A, Pereira LSM, et al. Efficacy of conservative therapy in older people with nonspecific low back pain: A systematic review with meta-analysis and GRADE recommendations. Archives of gerontology and geriatrics. 2020;90:104177.

An Q-Y, Wang L, Yuan P, Zhao Q-H, Gong S-G, Zhang R, et al. Effectiveness and safety of exercise training and rehabilitation in chronic thromboembolic pulmonary hypertension: a systematic review and meta-analysis. Annals of palliative medicine. 2021;10(7):8134-46.

Anand V, Garg S, Garg J, Bano S, Pritzker M. Impact of Exercise Training on Cardiac Function Among Patients With Type 2 Diabetes: A SYSTEMATIC REVIEW AND META-ANALYSIS. J Cardiopulm Rehabil Prev. 2018;38(6):358-65.

Anderson L, Oldridge N, Thompson DR, Zwisler A-D, Rees K, Martin N, et al. Exercise-Based Cardiac Rehabilitation for Coronary Heart Disease: Cochrane Systematic Review and Meta-Analysis. Journal of the American College of Cardiology. 2016;67(1):1-12.

Anderson L, Thompson DR, Oldridge N, Zwisler A-D, Rees K, Martin N, et al. Exercise-based cardiac rehabilitation for coronary heart disease. The Cochrane database of systematic reviews. 2016(1):CD001800.

Andrade A, de Azevedo Klumb Steffens R, Sieczkowska SM, Peyre Tartaruga LA, Torres Vilarino G. A systematic review of the effects of strength training in patients with fibromyalgia: clinical outcomes and design considerations. Advances in rheumatology (London, England). 2018;58(1):36.

Ang J, Moussa R, Shaikh S, Mele S. Effects of aerobic exercise on asthma control and quality of life in adults: a systematic review. J Asthma. 2023;60(5):845-55.
Anjos JM, Neto MG, Dos Santos FS, Almeida KO, Bocchi EA, Lima Bitar YS, et al. The impact Of high-intensity interval training On functioning And health-related quality Of life In post-stroke patients: A systematic review With meta-analysis. Clin Rehabil. 2022;36(6):726-39.
Arora E, Maiya AG, Devasia T, Bhat R, Kamath G. Effect of Supervised Exercise Program on Individuals in Peripheral Arterial Disease with Type 2 Diabetes Mellitus - A Systematic Review. Curr Diabetes Rev. 2020;16(3):248-53.
Ashdown-Franks G, Williams J, Vancampfort D, Firth J, Schuch F, Hubbard K, et al. Is it possible for people with severe mental illness to sit less and move more? A systematic review of interventions to increase physical activity or reduce sedentary behaviour. Schizophrenia research. 2018;202:3-16.
Atlihan R, Kirk B, Duque G. Non-Pharmacological Interventions in Osteosarcopenia: A Systematic Review. The journal of nutrition, health & aging. 2021;25(1):25-32.
Baillet A, Zeboulon N, Gossec L, Combescure C, Bodin LA, Juvin R, et al. Efficacy of cardiorespiratory aerobic exercise in rheumatoid arthritis: meta-analysis of randomized controlled trials. Arthritis Care Res (Hoboken). 2010;62(7):984-92.
Bajwah S, Colquitt J, Loveman E, Bausewein C, Almond H, Oluyase A, et al. Pharmacological and nonpharmacological interventions to improve symptom control, functional exercise capacity and quality of life in interstitial lung disease: An evidence synthesis. ERJ Open Research. 2021;7(1):1-18.
Barbosa M, Andrade R, de Melo CA, Torres R. Community-Based Pulmonary Rehabilitation Programs in Individuals With COPD. Respir Care. 2022;67(5):579-93.
Barcellos FC, Santos IS, Umpierre D, Bohlke M, Hallal PC. Effects of exercise in the whole spectrum of chronic kidney disease: A systematic review. Clinical Kidney Journal. 2015;8(6):753-65.
Barreto PdS, Demougeot L, Pillard F, Lapeyre-Mestre M, Rolland Y. Exercise training for managing behavioral and psychological symptoms in people with dementia: A systematic review and meta-analysis. Ageing research reviews. 2015;24(Pt B):274-85.
Bartels EM, Juhl CB, Christensen R, Hagen KB, Danneskiold-Samsøe B, Dagfinrud H, et al. Aquatic exercise for the treatment of knee and hip osteoarthritis. Cochrane Database of Systematic Reviews. 2016(3).
Bartels EM, Lund H, Hagen KB, Dagfinrud H, Christensen R, Danneskiold-Samsoe B. Aquatic exercise for the treatment of knee and hip osteoarthritis. The Cochrane database of systematic reviews. 2007(4):CD005523.
Bartholdy C, Juhl C, Christensen R, Lund H, Zhang W, Henriksen M. The role of muscle strengthening in exercise therapy for knee osteoarthritis: A systematic review and meta-regression analysis of randomized trials. Semin Arthritis Rheum. 2017;47(1):9-21.
Batalik L, Winnige P, Dosbaba F, Vlazna D, Janikova A, Lloyd-Williams M. Home-Based Aerobic and Resistance Exercise Interventions in Cancer Patients and Survivors: A Systematic Review. Cancers. 2021;13(8):1915.
Beaudry R, Kruger C, Liang Y, Parliament M, Haykowsky M, McNeely M. Effect of supervised exercise on aerobic capacity in cancer survivors: Adherence and workload predict variance in effect. World J Metaanal. 2015;3:43-53.
Beier D, Callesen HE, Carlsen LN, Birkefoss K, Tomasdottir H, Wurtzen H, et al. Manual joint mobilisation techniques, supervised physical activity, psychological treatment, acupuncture and patient education in migraine treatment. A systematic review and meta-analysis. Cephalalgia : an international journal of headache. 2022;42(1):63-72.
Bekhet AH, Abdallah AR, Ismail HM, Genena DM, Osman NA, El Khatib A, et al. Benefits of Aerobic Exercise for Breast Cancer Survivors: A Systematic Review of Randomized Controlled Trials. Asian Pac J Cancer Prev. 2019;20(11):3197-209.
Bernier-Jean A, Beruni NA, Bondonno NP, Williams G, Teixeira-Pinto A, Craig JC, et al. Exercise training for adults undergoing maintenance dialysis. Cochrane Database of Systematic Reviews. 2022;2022(1):CD014653.
Bertozzi L, Gardenghi I, Turoni F, Villafane JH, Capra F, Guccione AA, et al. Effect of therapeutic exercise on pain and disability in the management of chronic nonspecific neck pain: systematic review and meta-analysis of randomized trials. Physical therapy. 2013;93(8):1026-36.
Bidonde J, Busch AJ, Schachter CL, Overend TJ, Kim SY, Goes SM, et al. Aerobic exercise training for adults with fibromyalgia. Cochrane Database of Systematic Reviews. 2017;2017(6):CD012700.
Bidonde J, Busch AJ, Webber SC, Schachter CL, Danyliw A, Overend TJ, et al. Aquatic exercise training for fibromyalgia. Cochrane Database of Systematic Reviews. 2014(10).
Blankevoort CG, van Heuvelen MJG, Boersma F, Luning H, de Jong J, Scherder EJA. Review of effects of physical activity on strength, balance, mobility and ADL performance in elderly subjects with dementia. Dementia and geriatric cognitive disorders. 2010;30(5):392-402.

Blokzijl F, Dieperink W, Keus F, Reneman MF, Mariani MA, Van Der Horst IC. Cardiac rehabilitation for patients having cardiac surgery: A systematic review. Journal of Cardiovascular Surgery. 2018;59(6):817-29.

Bluethmann SM, Bartholomew LK, Murphy CC, Vernon SW. Use of Theory in Behavior Change Interventions. Health education & behavior : the official publication of the Society for Public Health Education. 2017;44(2):245-53.

Boing L, Vieira MCS, Moratelli J, Bergmann A, Guimarães ACA. Effects of exercise on physical outcomes of breast cancer survivors receiving hormone therapy - A systematic review and meta-analysis. Maturitas. 2020;141:71-81.

Bonnevie T, Smondack P, Elkins M, Gouel B, Medrinal C, Combret Y, et al. Advanced telehealth technology improves home-based exercise therapy for people with stable chronic obstructive pulmonary disease: a systematic review. Journal of Physiotherapy (Elsevier). 2021;67(1):27-40.

Borges-Machado F, Silva N, Farinatti P, Poton R, Ribeiro O, Carvalho J. Effectiveness of Multicomponent Exercise Interventions in Older Adults With Dementia: A Meta-Analysis. The Gerontologist. 2021;61(8):e449-e62.

Boulé NG, Kenny GP, Haddad E, Wells GA, Sigal RJ. Meta-analysis of the effect of structured exercise training on cardiorespiratory fitness in Type 2 diabetes mellitus. Diabetologia. 2003;46(8):1071-81.

Bourke L, Homer KE, Thaha MA, Steed L, Rosario DJ, Robb KA, et al. Interventions to improve exercise behaviour in sedentary people living with and beyond cancer: a systematic review. Br J Cancer. 2014;110(4):831-41.

Bourscheid G, Just KR, Costa RR, Petry T, Danzmann LC, Pereira AH, et al. Effect of different physical training modalities on peak oxygeconsumptions in post-acute myocardial infarction patients: Systematic review and meta-analysis. Jornal Vascular Brasileiro. 2021;20:e20210056.

Bradley JM, Moran F, Greenstone M. Physical training for bronchiectasis. Cochrane Database of Systematic Reviews. 2002:N.PAG-N.PAG.

Bradt J, Shim M, Goodill SW. Dance/movement therapy for improving psychological and physical outcomes in cancer patients. The Cochrane database of systematic reviews. 2015;1:CD007103.

Bravo C, Skjaerven LH, Guitard Sein-Echaluce L, Catalan-Matamoros D. Effectiveness of movement and body awareness therapies in patients with fibromyalgia: a systematic review and meta-analysis. European journal of physical and rehabilitation medicine. 2019;55(5):646-57.

Braz de Oliveira MP, Moreira Padovez RFC, Serrao PRMDS, de Noronha MA, Cezar NOC, Andrade LP. Effectiveness of physical exercise at improving functional capacity in older adults living with Alzheimer's disease: a systematic review of randomized controlled trials. Disability and rehabilitation. 2022:1-12.

Brazzelli M, Saunders DH, Greig CA, Mead GE. Physical fitness training for stroke patients. Cochrane Database Syst Rev. 2011(11):Cd003316.

Brett L, Traynor V, Stapley P. Effects of Physical Exercise on Health and Well-Being of Individuals Living With a Dementia in Nursing Homes: A Systematic Review. Journal of the American Medical Directors Association. 2016;17(2):104-16.

Breyley-Smith A, Mousa A, Teede HJ, Johnson NA, Sabag A. The Effect of Exercise on Cardiometabolic Risk Factors in Women with Polycystic Ovary Syndrome: A Systematic Review and Meta-Analysis. International journal of environmental research and public health. 2022;19(3).

Briani RV, Ferreira AS, Pazzinatto MF, Pappas E, De Oliveira Silva D, Azevedo FMd. What interventions can improve quality of life or psychosocial factors of individuals with knee osteoarthritis? A systematic review with meta-analysis of primary outcomes from randomised controlled trials. British journal of sports medicine. 2018;52(16):1031-8.

Brienesse LA, Emerson MN. Effects of Resistance Training for People With Parkinson's Disease: A Systematic Review. Journal of the American Medical Directors Association. 2013;14(4):236-41.

Broderick J, Knowles A, Chadwick J, Vancampfort D. Yoga versus standard care for schizophrenia. Cochrane Database of Systematic Reviews. 2015(10).

Broderick J, Knowles A, Chadwick J, Vancampfort D. Yoga vs Standard Care for Schizophrenia. Schizophrenia bulletin. 2016;42(1):15-7.

Broderick J, Vancampfort D. Yoga as part of a package of care versus standard care for schizophrenia. The Cochrane database of systematic reviews. 2017;9:CD012145.

Brox JI, Storheim K, Grotle M, Tveito TH, Indahl A, Eriksen HR. Systematic review of back schools, brief education, and fear-avoidance training for chronic low back pain. The spine journal : official journal of the North American Spine Society. 2008;8(6):948-58.

Buffart LM, Kalter J, Sweegers MG, Courneya KS, Newton RU, Aaronson NK, et al. Effects and moderators of exercise on quality of life and physical function in patients with cancer: An individual patient data meta-analysis of 34 RCTs. Cancer Treat Rev. 2017;52:91-104.

Burton E, Cavalheri V, Adams R, Browne CO, Bovery-Spencer P, Fenton AM, et al. Effectiveness of exercise programs to reduce falls in older people with dementia living in the community: a systematic review and meta-analysis. Clinical interventions in aging. 2015;10:421-34.
Burton M, Valet M, Caty G, Aboubakar F, Reychler G. Telerehabilitation physical exercise for patients with lung cancer through the course of their disease: A systematic review. Journal of telemedicine and telecare. 2022:1357633X221094200.
Busch AJ, Barber KA, Overend TJ, Peloso PMJ, Schachter CL. Exercise for treating fibromyalgia syndrome. Cochrane Database of Systematic Reviews. 2007(4).
Buzzetti E, Linden A, Best LMJ, Madden AM, Roberts D, Chase TJG, et al. Lifestyle modifications for nonalcohol-related fatty liver disease: a network meta-analysis. Cochrane Database of Systematic Reviews. 2021;2021(6):CD013156.
Byström MG, Rasmussen-Barr E, Grooten WJA. Motor control exercises reduces pain and disability in chronic and recurrent low back pain: a meta-analysis. Spine (03622436). 2013;38(6):E350-8.
Cai H, Li G, Zhang P, Xu D, Chen L. Effect of exercise on the quality of life in type 2 diabetes mellitus: a systematic review. Qual Life Res. 2017;26(3):515-30.
Canales-Díaz MB, Olivares-Valenzuela C, Ramírez-Arriagada A, Cruz-Montecinos C, Vilaró J, Torres-Castro R, et al. Clinical Effects of Rehabilitation on Balance in People With Chronic Obstructive Pulmonary Disease: A Systematic Review and Meta-Analysis. Front Med (Lausanne). 2022;9:868316.
Candelaria D, Randall S, Ladak L, Gallagher R. Health-related quality of life and exercise-based cardiac rehabilitation in contemporary acute coronary syndrome patients: A systematic review and meta-analysis. Quality of Life Research: An International Journal of Quality of Life Aspects of Treatment, Care & Rehabilitation. 2020;29(3):579-92.
Cao A, Feng F, Zhang L, Zhou X. Baduanjin exercise for chronic obstructive pulmonary disease: an updated systematic review and meta-analysis. Clinical rehabilitation. 2020;34(8):1004-13.
Carayol M, Bernard P, Boiche J, Riou F, Mercier B, Cousson-Gelie F, et al. Psychological effect of exercise in women with breast cancer receiving adjuvant therapy: what is the optimal dose needed? Annals of oncology : official journal of the European Society for Medical Oncology. 2013;24(2):291-300.
Carayol M, Bernard P, Boiche J, Riou F, Mercier B, Florence CG, et al. Psychological impact of exercise in women with breast cancer receiving adjuvant therapy: What is the optimal dose needed? Journal of Clinical Oncology. 2012;30(15 SUPPL. 1).
Carayol M, Delpierre C, Bernard P, Ninot G. Population-, intervention- and methodology-related characteristics of clinical trials impact exercise efficacy during adjuvant therapy for breast cancer: a meta-regression analysis. Psycho-oncology. 2015;24(7):737-47.
Carneiro L, Afonso J, Ramirez-Campillo R, Murawska-Cialowciz E, Marques A, Clemente FM. The Effects of Exclusively Resistance Training-Based Supervised Programs in People with Depression: A Systematic Review and Meta-Analysis of Randomized Controlled Trials. International journal of environmental research and public health. 2020;17(18).
Carpenter R, DiChiacchio T, Barker K. Interventions for self-management of type 2 diabetes: An integrative review. International Journal of Nursing Sciences. 2019;6(1):70-91.
Carson KV, Chandratilleke MG, Picot J, Brinn MP, Esterman AJ, Smith BJ. Physical training for asthma. Cochrane Database Syst Rev. 2013(9):Cd001116.
Cavalheri V, Burtin C, Formico VR, Nonoyama ML, Jenkins S, Spruit MA, et al. Exercise training undertaken by people within 12 months of lung resection for non-small cell lung cancer. Cochrane Database of Systematic Reviews. 2019(6).
Cavalheri V, Tahirah F, Nonoyama M, Jenkins S, Hill K. Exercise training undertaken by people within 12 months of lung resection for non-small cell lung cancer. Cochrane Database of Systematic Reviews. 2013(7):N.PAG-N.PAG.
Cavalheri V, Tahirah F, Nonoyama M, Jenkins S, Hill K. Exercise training for people following lung resection for non-small cell lung cancer - A Cochrane systematic review. Cancer treatment reviews. 2014;40(4):585-94.
Ceballos-Laita L, Estébanez-de-Miguel E, Martín-Nieto G, Bueno-Gracia E, Fortún-Agúd M, Jiménez-Del-Barrio S. Effects of non-pharmacological conservative treatment on pain, range of motion and physical function in patients with mild to moderate hip osteoarthritis. A systematic review. Complement Ther Med. 2019;42:214-22.
Cerrillo-Urbina AJ, Garcia-Hermoso A, Sanchez-Lopez M, Martinez-Vizcaino V. Effect of exercise programs on symptoms of fibromyalgia in peri-menopausal age women: A systematic review and meta-analysis of randomized controlled trials. MYOPAIN. 2015;23(1-2):56-70.
Chair SY, Zou H, Cao X. A systematic review of effects of recorded music listening during exercise on physical activity adherence and health outcomes in patients with coronary heart disease. Annals of physical and rehabilitation medicine. 2021;64(2):101447.

Chair SY, Zou H, Cao X. Effects of Exercise Therapy for Adults With Coronary Heart Disease: A Systematic Review and Meta-analysis of Randomized Controlled Trials. The Journal of cardiovascular nursing. 2021;36(1):56-77.

Chambers D, Bagnall A-M, Hempel S, Forbes C. Interventions for the treatment, management and rehabilitation of patients with chronic fatigue syndrome/myalgic encephalomyelitis: an updated systematic review. Journal of the Royal Society of Medicine. 2006;99(10):506-20.

Chan CL-W, Wang C-W, Ho RT-H, Ho AH-Y, Ziea ET-C, Taam Wong VC-W, et al. A Systematic Review of the Effectiveness of Qigong Exercise in Cardiac Rehabilitation. American Journal of Chinese Medicine. 2012;40(2):255-67.

Chan E, Giallauria F, Vigorito C, Smart NA. Exercise training in heart failure patients with preserved ejection fraction: a systematic review and meta-analysis. Monaldi Arch Chest Dis. 2016;86(1-2):759.

Chan KGF, Jiang Y, Choo WT, Ramachandran HJ, Lin Y, Wang W. Effects of exergaming on functional outcomes in people with chronic stroke: A systematic review and meta-analysis. Journal of Advanced Nursing (John Wiley & Sons, Inc). 2022;78(4):929-46.

Cheema B, Chan D, Fahey P, Atlantis E. Effect of Progressive Resistance Training on Measures of Skeletal Muscle Hypertrophy, Muscular Strength and Health-Related Quality of Life in Patients with Chronic Kidney Disease: A Systematic Review and Meta-Analysis. Sports Medicine. 2014;44(8):1125-38.

Cheema BS, Kilbreath SL, Fahey PP, Delaney GP, Atlantis E. Safety and efficacy of progressive resistance training in breast cancer: a systematic review and meta-analysis. Breast Cancer Res Treat. 2014;148(2):249-68.

Cheema BSB, Fiatarone Singh MA. Exercise training in patients receiving maintenance hemodialysis: A systematic review of clinical trials. American Journal of Nephrology. 2005;25(4):352-64.

Chen X, Savarese G, Cai Y, Ma L, Lundborg CS, Jiang W, et al. Tai Chi and Qigong Practices for Chronic Heart Failure: A Systematic Review and Meta-Analysis of Randomized Controlled Trials. Evidence-based Complementary & Alternative Medicine (eCAM). 2020:1-15.

Chen YC, Tsai JC, Liou YM, Chan P. Effectiveness of endurance exercise training in patients with coronary artery disease: A meta-analysis of randomised controlled trials. European journal of cardiovascular nursing. 2017;16(5):397-408.

Chen YM, Li Y. Safety and efficacy of exercise training in elderly heart failure patients: a systematic review and meta-analysis. Int J Clin Pract. 2013;67(11):1192-8.

Cheng CA, Chiu YW, Wu D, Kuan YC, Chen SN, Tam KW. Effectiveness of Tai Chi on fibromyalgia patients: A meta-analysis of randomized controlled trials. Complementary therapies in medicine. 2019;46:1-8.

Cheng KKF, Lim YTE, Koh ZM, Tam WWS. Home-based multidimensional survivorship programmes for breast cancer survivors. The Cochrane database of systematic reviews. 2017;8:CD011152.

Chien CL, Lee CM, Wu YW, Chen TA, Wu YT. Home-based exercise increases exercise capacity but not quality of life in people with chronic heart failure: a systematic review. Aust J Physiother. 2008;54(2):87-93.

Chipperfield K, Brooker J, Fletcher J, Burney S. The impact of physical activity on psychosocial outcomes in men receiving androgen deprivation therapy for prostate cancer: a systematic review. Health psychology : official journal of the Division of Health Psychology, American Psychological Association. 2014;33(11):1288-97.

Choo YJ, Cho CW, Chang MC. Effects of supervised exercise on aerobic capacity and quality of life in patients with chronic liver disease and patients who underwent liver transplantation: a systematic review and meta-analysis. International journal of rehabilitation research Internationale Zeitschrift fur Rehabilitationsforschung Revue internationale de recherches de readaptation. 2022;45(1):1-11.

Cinthuja P, Krishnamoorthy N, Shivapatham G. Effective interventions to improve long-term physiotherapy exercise adherence among patients with lower limb osteoarthritis. A systematic review. BMC musculoskeletal disorders. 2022;23(1):1-11.

Claes J, Buys R, Budts W, Smart N, Cornelissen VA. Longer-term effects of home-based exercise interventions on exercise capacity and physical activity in coronary artery disease patients: A systematic review and meta-analysis. European journal of preventive cardiology. 2017;24(3):244-56.

Clark AM, Hartling L, Vandermeer B, McAlister FA. Meta-analysis: secondary prevention programs for patients with coronary artery disease. Ann Intern Med. 2005;143(9):659-72.

Colledge F, Gerber M, Puhse U, Ludyga S. Anaerobic Exercise Training in the Therapy of Substance Use Disorders: A Systematic Review. Frontiers in Psychiatry. 2018;9:644.

Conceicao LSR, Neto MG, do Amaral MAS, Martins-Filho PRS, Oliveira Carvalho V. Effect of dance therapy on blood pressure and exercise capacity of individuals with hypertension: A systematic review and meta-analysis. International journal of cardiology. 2016;220:553-7.

Cooney GM, Dwan K, Greig CA, Lawlor DA, Rimer J, Waugh FR, et al. Exercise for depression. The Cochrane database of systematic reviews. 2013(9):CD004366.

Cornish AK, Broadbent S, Cheema BS. Interval training for patients with coronary artery disease: A systematic review. European journal of applied physiology. 2011;111(4):579-89.
Coventry PA, Hind D. Comprehensive pulmonary rehabilitation for anxiety and depression in adults with chronic obstructive pulmonary disease: Systematic review and meta-analysis. Journal of psychosomatic research. 2007;63(5):551-65.
Cox NS, Dal Corso S, Hansen H, McDonald CF, Hill CJ, Zanaboni P, et al. Telerehabilitation for chronic respiratory disease. The Cochrane database of systematic reviews. 2021;1:CD013040.
Cramer H, Haller H, Klose P, Ward L, Chung VCH, Lauche R. The risks and benefits of yoga for patients with chronic obstructive pulmonary disease: a systematic review and meta-analysis. Clinical rehabilitation. 2019;33(12):1847-62.
Cramer H, Lange S, Klose P, Paul A, Dobos G. Yoga for breast cancer patients and survivors: a systematic review and meta-analysis. BMC Cancer. 2012;12:412.
Cramer H, Lauche R, Klose P, Dobos G, Langhorst J. A systematic review and meta-analysis of exercise interventions for colorectal cancer patients. Eur J Cancer Care (Engl). 2014;23(1):3-14.
Cramer H, Lauche R, Klose P, Lange S, Langhorst J, Dobos GJ. Yoga for improving health-related quality of life, mental health and cancer-related symptoms in women diagnosed with breast cancer. The Cochrane database of systematic reviews. 2017;1:CD010802.
Cramer H, Lauche R, Klose P, Langhorst J, Dobos G. Yoga for schizophrenia: a systematic review and meta-analysis. BMC psychiatry. 2013;13:32.
Cramp F, Berry J, Gardiner M, Smith F, Stephens D. Health Behaviour Change Interventions for the Promotion of Physical Activity in Rheumatoid Arthritis: A Systematic Review. Musculoskeletal care. 2013;11(4):238-47.
Cramp F, Hewlett S, Almeida C, Kirwan JR, Choy EH, Chalder T, et al. Non-pharmacological interventions for fatigue in rheumatoid arthritis. Cochrane Database of Systematic Reviews. 2013(8):N.PAG-N.PAG.
Cugusi L, Yeo TJ, Bassareo PP, Manca A, Mercuro G, Kaski JC. Nordic walking and cardiovascular disease prevention: A systematic review of randomized controlled trials. European journal of preventive cardiology. 2017;24(1 Supplement 1):S46.
Cui J, Liu F, Liu X, Li R, Chen X, Zeng H. The Impact of Qigong and Tai Chi Exercise on Drug Addiction: A Systematic Review and Meta-Analysis. Frontiers in Psychiatry. 2022;13:826187.
Ćwiękała-Lewis KJ, Gallek M, Taylor-Piliae RE. The effects of Tai Chi on physical function and well-being among persons with Parkinson's Disease: A systematic review. J Bodyw Mov Ther. 2017;21(2):414-21.
Da Campo L, Hauck M, Marcolino MAZ, Pinheiro D, Plentz RDM, Cechetti F. Effects of aerobic exercise using cycle ergometry on balance and functional capacity in post-stroke patients: a systematic review and meta-analysis of randomised clinical trials. Disabil Rehabil. 2021;43(11):1558-64.
Dauwan M, Begemann MJH, Heringa SM, Sommer IE. Exercise Improves Clinical Symptoms, Quality of Life, Global Functioning, and Depression in Schizophrenia: A Systematic Review and Meta-analysis. Schizophrenia bulletin. 2016;42(3):588-99.
Davies EJ, Moxham T, Rees K, Singh S, Coats AJ, Ebrahim S, et al. Exercise based rehabilitation for heart failure. The Cochrane database of systematic reviews. 2010(4):CD003331.
Davis SP, Bolin LP, Crane PB, Crandell J. Non-pharmacological interventions for anxiety and depression in adults with inflammatory bowel disease: A systematic review and meta-analysis. Frontiers in Psychology. 2020;11.
de Almeida SIL, Gomes da Silva M, de Dias Marques ASP. Home-based physical activity programs for people with dementia: Systematic review and meta-analysis. The Gerontologist. 2020;60(8):e600-e8.
de Kam D, Smulders E, Weerdesteyn V, Smits-Engelsman BCM. Exercise interventions to reduce fall-related fractures and their risk factors in individuals with low bone density: a systematic review of randomized controlled trials. Osteoporosis international : a journal established as result of cooperation between the European Foundation for Osteoporosis and the National Osteoporosis Foundation of the USA. 2009;20(12):2111-25.
de Oliveira MPB, Lobato DFM, Smaili SM, Carvalho C, Borges JBC. Effect of aerobic exercise on functional capacity and quality of life in individuals with Parkinson's disease: A systematic review of randomized controlled trials. Arch Gerontol Geriatr. 2021;95:104422.
de Souto Barreto P, Maltais M, Rosendahl E, Vellas B, Bourdel-Marchasson I, Lamb SE, et al. Exercise Effects on Falls, Fractures, Hospitalizations, and Mortality in Older Adults With Dementia: An Individual-Level Patient Data Meta-analysis. The journals of gerontology Series A, Biological sciences and medical sciences. 2021;76(9):e203-e12.
Deng N, Gu T, Zhao Q, Zhang X, Zhao F, He H. Effects of telephone support on exercise capacity and quality of life in patients with chronic obstructive pulmonary disease: a meta-analysis. Psychology, Health & Medicine. 2018;23(8):917-33.

Dennett AM, Peiris CL, Shields N, Prendergast LA, Taylor NF. Moderate-intensity exercise reduces fatigue and improves mobility in cancer survivors: a systematic review and meta-regression. J Physiother. 2016;62(2):68-82.
Di Lorito C, Bosco A, Booth V, Goldberg S, Harwood RH, Van der Wardt V. Adherence to exercise interventions in older people with mild cognitive impairment and dementia: A systematic review and meta-analysis. Preventive Medicine Reports. 2020;19:101139.
Ding M, Zhang W, Li K, Chen X. Effectiveness of T'ai Chi and Qigong on Chronic Obstructive Pulmonary Disease: A Systematic Review and Meta-Analysis. Journal of Alternative & Complementary Medicine. 2014;20(2):79-86.
Dong J, Li Z, Luo L, Xie H. Efficacy of pulmonary rehabilitation in improving the quality of life for patients with chronic obstructive pulmonary disease: Evidence based on nineteen randomized controlled trials. International Journal of Surgery. 2020;73:78-86.
Dorsch S, Ada L, Alloggia D. Progressive resistance training increases strength after stroke but this may not carry over to activity: a systematic review. J Physiother. 2018;64(2):84-90.
Duan L, Xu Y, Li M. Effects of Mind-Body Exercise in Cancer Survivors: A Systematic Review and Meta-Analysis. Evidence-based Complementary and Alternative Medicine. 2020;2020:7607161.
Duff OM, Walsh DM, Furlong BA, O'Connor NE, Moran KA, Woods CB. Behavior Change Techniques in Physical Activity eHealth Interventions for People With Cardiovascular Disease: Systematic Review. Journal of medical Internet research. 2017;19(8):e281.
Effing T, Monninkhof EE, van der Valk PP, Zielhuis GG, Walters EH, van der Palen JJ, et al. Self management for patients with chronic obstructive pulmonary disease. Cochrane Database of Systematic Reviews. 2014(3):N.PAG-N.PAG.
Eichenberger PA, Diener SN, Kofmehl R, Spengler CM. Effects of exercise training on airway hyperreactivity in asthma: a systematic review and meta-analysis. Sports Med. 2013;43(11):1157-70.
Emmanouilidis S, Hackney ME, Slade SC, Heng H, Jazayeri D, Morris ME. Dance Is an Accessible Physical Activity for People with Parkinson's Disease. Parkinson's Disease (20420080). 2021:1-20.
English C, Hillier S. Circuit class therapy for improving mobility after stroke: a systematic review. J Rehabil Med. 2011;43(7):565-71.
English C, Hillier SL. Circuit class therapy for improving mobility after stroke. Cochrane Database Syst Rev. 2010;2010(7):Cd007513.
Ertürk G, Günday Ç, Evrendilek H, Sağır K, Aslan GK. Effects of high intensity interval training and sprint interval training in patients with asthma: a systematic review. J Asthma. 2022;59(11):2292-304.
Escalante Y, García-Hermoso A, Saavedra JM. Effects of exercise on functional aerobic capacity in lower limb osteoarthritis: a systematic review. J Sci Med Sport. 2011;14(3):190-8.
Fakhry F, van de Luijtgaarden KM, Bax L, den Hoed PT, Hunink MG, Rouwet EV, et al. Supervised walking therapy in patients with intermittent claudication. J Vasc Surg. 2012;56(4):1132-42.
Feng Z, Wang J, Xie Y, Li J. Effects of exercise-based pulmonary rehabilitation on adults with asthma: a systematic review and meta-analysis. Respir Res. 2021;22(1):33.
Fernandopulle S, Perry M, Manlapaz D, Jayakaran P. Effect of Land-Based Generic Physical Activity Interventions on Pain, Physical Function, and Physical Performance in Hip and Knee Osteoarthritis: A Systematic Review and Meta-Analysis. Am J Phys Med Rehabil. 2017;96(11):773-92.
Ferreira ML, Smeets RJEM, Kamper SJ, Ferreira PH, Machado LAC. Can we explain heterogeneity among randomized clinical trials of exercise for chronic back pain? A meta-regression analysis of randomized controlled trials. Physical therapy. 2010;90(10):1383-403.
Ferreira PH, Ferreira ML, Maher CG, Herbert RD, Refshauge K. Specific stabilisation exercise for spinal and pelvic pain: a systematic review. The Australian journal of physiotherapy. 2006;52(2):79-88.
Finlay A, Wittert G, Short CE. A systematic review of physical activity-based behaviour change interventions reaching men with prostate cancer. Journal of Cancer Survivorship. 2018;12(4):571-91.
Firth J, Cotter J, Elliott R, French P, Yung AR. A systematic review and meta-analysis of exercise interventions in schizophrenia patients. Psychological medicine. 2015;45(7):1343-61.
Forbes D, Forbes SC, Blake CM, Thiessen EJ, Forbes S. Exercise programs for people with dementia. The Cochrane database of systematic reviews. 2015(4):CD006489.
Ford CG, Vowles KE, Smith BW, Kinney AY. Mindfulness and Meditative Movement Interventions for Men Living With Cancer: A Meta-analysis. Ann Behav Med. 2020;54(5):360-73.
Fox B, Hodgkinson B, Parker D. The effects of physical exercise on functional performance, quality of life, cognitive impairment and physical activity levels for older adults aged 65 years and older with a diagnosis of dementia: A systematic review. JBI database of systematic reviews and implementation reports. 2014;12(9):158-276.

Francis T, Kabboul N, Rac V, Mitsakakis N, Pechlivanoglou P, Bielecki J, et al. The Effect of Cardiac Rehabilitation on Health-Related Quality of Life in Patients With Coronary Artery Disease: A Meta-analysis. Canadian Journal of Cardiology. 2019;35(3):352-64.

Fransen M, McConnell S, Bell M. Exercise for osteoarthritis of the hip or knee. Cochrane Database Syst Rev. 2003(3):Cd004286.

Furmaniak AC, Menig M, Markes MH. Exercise for women receiving adjuvant therapy for breast cancer. The Cochrane database of systematic reviews. 2016;9:CD005001.

Gao M, Huang Y, Wang Q, Liu K, Sun G. Effects of High-Intensity Interval Training on Pulmonary Function and Exercise Capacity in Individuals with Chronic Obstructive Pulmonary Disease: A Meta-Analysis and Systematic Review. Adv Ther. 2022;39(1):94-116.

Gao P, Tang F, Liu W, He K, Mo Y. Effect of liuzijue qigong on patients with stable chronic obstructive pulmonary disease: A systematic review and meta-analysis. Medicine (Baltimore). 2021;100(41):e27344.

García-Bravo S, Cuesta-Gómez A, Campuzano-Ruiz R, López-Navas MJ, Domínguez-Paniagua J, Araújo-Narváez A, et al. Virtual reality and video games in cardiac rehabilitation programs A systematic review. Disability and Rehabilitation: An International, Multidisciplinary Journal. 2021;43(4):448-57.

Gerritsen JK, Vincent AJ. Exercise improves quality of life in patients with cancer: a systematic review and meta-analysis of randomised controlled trials. Br J Sports Med. 2016;50(13):796-803.

Gibbs JC, Macintyre NJ, Ponzano M, Templeton JA, Thabane L, Papaioannou A, et al. Exercise for improving outcomes after osteoporotic vertebral fracture. Cochrane Database of Systematic Reviews. 2019;2019(7):CD008618.

Giesen ES, Deimel H, Bloch W. Clinical exercise interventions in alcohol use disorders: a systematic review. Journal of substance abuse treatment. 2015;52:1-9.

Gimenez-Meseguer J, Tortosa-Martinez J, Cortell-Tormo JM. The Benefits of Physical Exercise on Mental Disorders and Quality of Life in Substance Use Disorders Patients. Systematic Review and Meta-Analysis. International journal of environmental research and public health. 2020;17(10).

Gollan R, Ernst M, Lieker E, Caro-Valenzuela J, Monsef I, Dresen A, et al. Effects of Resistance Training on Motor- and Non-Motor Symptoms in Patients with Parkinson's Disease: A Systematic Review and Meta-Analysis. J Parkinsons Dis. 2022;12(6):1783-806.

Gomes Neto M, de Lacerda FFR, Lopes AA, Martinez BP, Saquetto MB. Intradialytic exercise training modalities on physical functioning and health-related quality of life in patients undergoing maintenance hemodialysis: systematic review and meta-analysis. Clinical rehabilitation. 2018;32(9):1189-202.

Gomes Neto M, Menezes MA, Oliveira Carvalho V. Dance therapy in patients with chronic heart failure: a systematic review and a meta-analysis. Clinical rehabilitation. 2014;28(12):1172-9.

Goodwin VA, Richards SH, Taylor RS, Taylor AH, Campbell JL. The effectiveness of exercise interventions for people with Parkinson's disease: a systematic review and meta-analysis. Mov Disord. 2008;23(5):631-40.

Gopaluni S, Sherif M, Ahmadouk NA. Interventions for chronic kidney disease-associated restless legs syndrome. Cochrane Database of Systematic Reviews. 2016;2016(11):CD010690.

Gorczynski P, Faulkner G. Exercise therapy for schizophrenia. The Cochrane database of systematic reviews. 2010(5):CD004412.

Grande AJ, Silva V, Andriolo BN, Riera R, Parra SA, Peccin MS. Water-based exercise for adults with asthma. Cochrane Database Syst Rev. 2014(7):Cd010456.

Grande AJ, Silva V, Sawaris Neto L, Teixeira Basmage JP, Peccin MS, Maddocks M. Exercise for cancer cachexia in adults. Cochrane Database of Systematic Reviews. 2021(3).

Graven C, Brock K, Hill K, Joubert L. Are rehabilitation and/or care co-ordination interventions delivered in the community effective in reducing depression, facilitating participation and improving quality of life after stroke? Disability and rehabilitation. 2011;33(17-18):1501-20.

Gu Q, Wu SJ, Zheng Y, Zhang Y, Liu C, Hou JC, et al. Tai Chi Exercise for Patients with Chronic Heart Failure: A Meta-analysis of Randomized Controlled Trials. Am J Phys Med Rehabil. 2017;96(10):706-16.

Gu Y, Dennis SM. Are falls prevention programs effective at reducing the risk factors for falls in people with type-2 diabetes mellitus and peripheral neuropathy: A systematic review with narrative synthesis. J Diabetes Complications. 2017;31(2):504-16.

Gunn H, Markevics S, Haas B, Marsden J, Freeman J. Systematic Review: The Effectiveness of Interventions to Reduce Falls and Improve Balance in Adults With Multiple Sclerosis. Arch Phys Med Rehabil. 2015;96(10):1898-912.

Guo C, Xiang G, Xie L, Liu Z, Zhang X, Wu Q, et al. Effects of Tai Chi training on the physical and mental health status in patients with chronic obstructive pulmonary disease: A systematic review and meta-analysis. Journal of Thoracic Disease. 2020;12(3):504-21.

Guo J-B, Chen B-L, Lu Y-M, Zhang W-Y, Zhu Z-J, Yang Y-J, et al. Tai Chi for improving cardiopulmonary function and quality of life in patients with chronic obstructive pulmonary disease: a systematic review and meta-analysis. Clinical rehabilitation. 2016;30(8):750-64.

Guzman J, Esmail R, Karjalainen K, Malmivaara A, Irvin E, Bombardier C. Multidisciplinary bio-psycho-social rehabilitation for chronic low back pain. The Cochrane database of systematic reviews. 2002(1):CD000963.

Haixia C, Peijun L, Ning L, Zhengrong W, Weibing W, Jihong W, et al. Rehabilitation effects of land and water-based aerobic exercise on lung function, dyspnea, and exercise capacity in patients with chronic obstructive pulmonary disease: A systematic review and meta-analysis. Medicine. 2021;100(33):1-13.

Hall M, Dobson F, Van Ginckel A, Nelligan RK, Collins NJ, Smith MD, et al. Comparative effectiveness of exercise programs for psychological well-being in knee osteoarthritis: A systematic review and network meta-analysis. Seminars in arthritis and rheumatism. 2021;51(5):1023-32.

Hallgren M, Vancampfort D, Giesen ES, Lundin A, Stubbs B. Exercise as treatment for alcohol use disorders: systematic review and meta-analysis. British journal of sports medicine. 2017;51(14):1058-64.

Hammer MJ, Ercolano EA, Wright F, Dickson VV, Chyun D, Melkus GD. Self-management for adult patients with cancer an integrative review: An integrative review. Cancer nursing. 2015;38(2):E10-E26.

Han A, Judd M, Welch V, Wu T, Tugwell P, Wells GA. Tai chi for treating rheumatoid arthritis. Cochrane Database of Systematic Reviews. 2004:N.PAG-N.PAG.

Hanada M, Kasawara KT, Mathur S, Rozenberg D, Kozu R, Hassan SA, et al. Aerobic and breathing exercises improve dyspnea, exercise capacity and quality of life in idiopathic pulmonary fibrosis patients: systematic review and meta-analysis. J Thorac Dis. 2020;12(3):1041-55.

Hansen S, Mikkelsen LR, Overgaard S, Mechlenburg I. Effectiveness of supervised resistance training for patients with hip osteoarthritis - a systematic review. Dan Med J. 2020;67(6).

Harder H, Parlour L, Jenkins V. Randomised controlled trials of yoga interventions for women with breast cancer: a systematic literature review. Support Care Cancer. 2012;20(12):3055-64.

Harris JE, Eng JJ. Strength training improves upper-limb function in individuals with stroke: a meta-analysis. Stroke. 2010;41(1):136-40.

Harwood AE, Russell S, Okwose NC, McGuire S, Jakovljevic DG, McGregor G. A systematic review of rehabilitation in chronic heart failure: evaluating the reporting of exercise interventions. ESC heart failure. 2021;8(5):3458-71.

Hauser W, Bernardy K, Arnold B, Offenbacher M, Schiltenwolf M. Efficacy of multicomponent treatment in fibromyalgia syndrome: A meta-analysis of randomized controlled clinical trials. Arthritis Care and Research. 2009;61(2):216-24.

Hauser W, Klose P, Langhorst J, Moradi B, Steinbach M, Schiltenwolf M, et al. Efficacy of different types of aerobic exercise in fibromyalgia syndrome: a systematic review and meta-analysis of randomised controlled trials. Arthritis research & therapy. 2010;12(3):R79.

Hegewald J, Wegewitz UE, Euler U, van Dijk JL, Adams J, Fishta A, et al. Interventions to support return to work for people with coronary heart disease. The Cochrane database of systematic reviews. 2019;3:CD010748.

Heikkinen SA, Quansah R, Jaakkola JJ, Jaakkola MS. Effects of regular exercise on adult asthma. Eur J Epidemiol. 2012;27(6):397-407.

Heiwe S, Jacobson SH. Exercise training for adults with chronic kidney disease. Cochrane Database of Systematic Reviews. 2011(10).

Heiwe S, Jacobson SH. Exercise Training in Adults With CKD: A Systematic Review and Meta-analysis. American Journal of Kidney Diseases. 2014;64(3):383-93.

Hempel S, Graham GD, Fu N, Estrada E, Chen AY, Miake-Lye I, et al. A systematic review of the effects of modifiable risk factor interventions on the progression of multiple sclerosis. Multiple Sclerosis. 2017;23(4):513-24.

Hernando-Garijo I, Jimenez-Del-Barrio S, Mingo-Gomez T, Medrano-de-la-Fuente R, Ceballos-Laita L. Effectiveness of non-pharmacological conservative therapies in adults with fibromyalgia: A systematic review of high-quality clinical trials. Journal of back and musculoskeletal rehabilitation. 2022;35(1):3-20.

Heron N, Kee F, Cardwell C, Tully MA, Donnelly M, Cupples ME. Secondary prevention lifestyle interventions initiated within 90 days after TIA or 'minor' stroke: a systematic review and meta-analysis of rehabilitation programmes. The British journal of general practice : the journal of the Royal College of General Practitioners. 2017;67(654):e57-e66.

Hindelang M, Kirsch F, Leidl R. Effectiveness of non-pharmacological COPD management on health-related quality of life - a systematic review. Expert review of pharmacoeconomics & outcomes research. 2020;20(1):79-91.

Hoffman M, Chaves G, Ribeiro-Samora GA, Britto RR, Parreira VF. Effects of pulmonary rehabilitation in lung transplant candidates: a systematic review. BMJ open. 2017;7(2):e013445.
Holland A, Hill C. Pulmonary rehabilitation for interstitial lung disease. Cochrane Database of Systematic Reviews. 2014(10):N.PAG-N.PAG.
Holland AE, Hill CJ, Jones AY, McDonald CF. Breathing exercises for chronic obstructive pulmonary disease. Cochrane Database of Systematic Reviews. 2012(10):N.PAG-N.PAG.
Hollings M, Mavros Y, Freeston J, Fiatarone Singh M. The effect of progressive resistance training on aerobic fitness and strength in adults with coronary heart disease: A systematic review and meta-analysis of randomised controlled trials. European journal of preventive cardiology. 2017;24(12):1242-59.
Howden EJ, Fassett RG, Isbel NM, Coombes JS. Exercise Training in Chronic Kidney Disease Patients. Sports Medicine. 2012;42(6):473-88.
Howland C, Wakefield B. Assessing telehealth interventions for physical activity and sedentary behavior self-management in adults with type 2 diabetes mellitus: An integrative review. Research in nursing & health. 2021;44(1):92-110.
Hu H, Xu A, Gao C, Wang Z, Wu X. The effect of physical exercise on rheumatoid arthritis: An overview of systematic reviews and meta-analysis. J Adv Nurs. 2021;77(2):506-22.
Hu L, Wang Y, Liu X, Ji X, Ma Y, Man S, et al. Tai Chi exercise can ameliorate physical and mental health of patients with knee osteoarthritis: systematic review and meta-analysis. Clin Rehabil. 2021;35(1):64-79.
Huang R, Palmer SC, Cao Y, Zhang H, Sun Y, Su W, et al. Cardiac Rehabilitation Programs for Chronic Heart Disease: A Bayesian Network Meta-analysis. The Canadian journal of cardiology. 2021;37(1):162-71.
Hunter EG, Gibson RW, Arbesman M, D'Amico M. Systematic Review of Occupational Therapy and Adult Cancer Rehabilitation: Part 1. Impact of Physical Activity and Symptom Management Interventions. The American journal of occupational therapy : official publication of the American Occupational Therapy Association. 2017;71(2):7102100030p1-p11.
Hurkmans E, van der Giesen FJ, Vliet Vlieland TP, Schoones J, Van den Ende EC. Dynamic exercise programs (aerobic capacity and/or muscle strength training) in patients with rheumatoid arthritis. Cochrane Database Syst Rev. 2009;2009(4):Cd006853.
Hurley M, Dickson K, Hallett R, Grant R, Hauari H, Walsh N, et al. Exercise interventions and patient beliefs for people with hip, knee or hip and knee osteoarthritis: a mixed methods review. The Cochrane database of systematic reviews. 2018;4:CD010842.
Hwang C-L, Chien C-L, Wu Y-T. Resistance training increases 6-minute walk distance in people with chronic heart failure: a systematic review. Journal of physiotherapy. 2010;56(2):87-96.
Irvine C, Taylor NF. Progressive resistance exercise improves glycaemic control in people with type 2 diabetes mellitus: a systematic review. Aust J Physiother. 2009;55(4):237-46.
Janssen SM, Connelly DM. The effects of exercise interventions on physical function tests and glycemic control in adults with type 2 diabetes: A systematic review. J Bodyw Mov Ther. 2021;28:283-93.
Jayakody K, Gunadasa S, Hosker C. Exercise for anxiety disorders: systematic review. Br J Sports Med. 2014;48(3):187-96.
Jayedi A, Emadi A, Shab-Bidar S. Dose-Dependent Effect of Supervised Aerobic Exercise on HbA(1c) in Patients with Type 2 Diabetes: A Meta-analysis of Randomized Controlled Trials. Sports Med. 2022;52(8):1919-38.
Jewiss D, Ostman C, Smart NA. The effect of resistance training on clinical outcomes in heart failure: A systematic review and meta-analysis. Int J Cardiol. 2016;221:674-81.
Jin X, Pan B, Wu H, Xu D. The effects of traditional Chinese exercise on hypertension: A systematic review and meta-analysis of randomized controlled trials. Medicine. 2019;98(3):e14049.
Jin X, Wang L, Liu S, Zhu L, Loprinzi PD, Fan X. The impact of mind-body exercises on motor function, depressive symptoms, and quality of life in parkinson's disease: A systematic review and meta-analysis. International journal of environmental research and public health. 2020;17(1):31.
Jolliffe JA, Rees K, Taylor RS, Thompson D, Oldridge N, Ebrahim S. Exercise-based rehabilitation for coronary heart disease. The Cochrane database of systematic reviews. 2001(1):CD001800.
Jones LW, Liang Y, Pituskin EN, Battaglini CL, Scott JM, Hornsby WE, et al. Effect of exercise training on peak oxygen consumption in patients with cancer: a meta-analysis. Oncologist. 2011;16(1):112-20.
Jordan RE, Majothi S, Heneghan NR, Blissett DB, Riley RD, Sitch AJ, et al. Supported self-management for patients with moderate to severe chronic obstructive pulmonary disease (COPD): an evidence synthesis and economic analysis. Health Technology Assessment. 2015;19(42):1-516.
Juhl C, Christensen R, Roos EM, Zhang W, Lund H. Impact of exercise type and dose on pain and disability in knee osteoarthritis: a systematic review and meta-regression analysis of randomized controlled trials. Arthritis Rheumatol. 2014;66(3):622-36.

Juvet LK, Thune I, Elvsaas I, Fors EA, Lundgren S, Bertheussen G, et al. The effect of exercise on fatigue and physical functioning in breast cancer patients during and after treatment and at 6 months follow-up: A meta-analysis. Breast. 2017;33:166-77.
Kaihara T, Intan-Goey V, Scherrenberg M, Falter M, Frederix I, Dendale P. Impact of activity trackers on secondary prevention in patients with coronary artery disease: a systematic review and meta-analysis. European journal of preventive cardiology. 2021.
Kamper SJ, Apeldoorn AT, Chiarotto A, Smeets RJ, Ostelo RW, Guzman J, et al. Multidisciplinary biopsychosocial rehabilitation for chronic low back pain. Cochrane Database of Systematic Reviews. 2014(9):N.PAG-N.PAG.
Kamper SJ, Apeldoorn AT, Chiarotto A, Smeets RJEM, Ostelo RWJG, Guzman J, et al. Multidisciplinary biopsychosocial rehabilitation for chronic low back pain: Cochrane systematic review and meta-analysis. BMJ (Clinical research ed). 2015;350:h444.
Karjalainen K, Malmivaara A, van Tulder M, Roine R, Jauhiainen M, Hurri H, et al. Multidisciplinary rehabilitation for fibromyalgia and musculoskeletal pain in working age adults. The Cochrane database of systematic reviews. 2000(2):CD001984.
Karssemeijer EGAE, Aaronson JAJ, Bossers WJW, Smits TT, Olde Rikkert MGMM, Kessels RPCR. Positive effects of combined cognitive and physical exercise training on cognitive function in older adults with mild cognitive impairment or dementia: A meta-analysis. Ageing research reviews. 2017;40:75-83.
Kato M, Kubo A, Nihei F, Ogano M, Takagi H. Effects of exercise training on exercise capacity, cardiac function, BMI, and quality of life in patients with atrial fibrillation: a meta-analysis of randomized-controlled trials. International journal of rehabilitation research Internationale Zeitschrift fur Rehabilitationsforschung Revue internationale de recherches de readaptation. 2017;40(3):193-201.
Keller-Varady K, Varady PA, Roh A, Schmitt A, Falkai P, Hasan A, et al. A systematic review of trials investigating strength training in schizophrenia spectrum disorders. Schizophrenia research. 2018;192:64-8.
Kelly C, Grundy S, Lynes D, Evans DJ, Gudur S, Milan SJ, et al. Self-management for bronchiectasis. The Cochrane database of systematic reviews. 2018;2:CD012528.
Kendall BJ, Gothe NP. Effect of Aerobic Exercise Interventions on Mobility among Stroke Patients: A Systematic Review. Am J Phys Med Rehabil. 2016;95(3):214-24.
Khanpour Ardestani S, Karkhaneh M, Stein E, Punja S, Junqueira DR, Kuzmyn T, et al. Systematic Review of Mind-Body Interventions to Treat Myalgic Encephalomyelitis/Chronic Fatigue Syndrome. Medicina (Kaunas, Lithuania). 2021;57(7).
Kim D-Y, Lee J-S, Park S-Y, Kim S-J, Son C-G. Systematic review of randomized controlled trials for chronic fatigue syndrome/myalgic encephalomyelitis (CFS/ME). Journal of translational medicine. 2020;18(1):7.
Knols R, Aaronson NK, Uebelhart D, Fransen J, Aufdemkampe G. Physical exercise in cancer patients during and after medical treatment: a systematic review of randomized and controlled clinical trials. J Clin Oncol. 2005;23(16):3830-42.
Ko SH, Chi CC, Yeh ML, Wang SH, Tsai YS, Hsu MY. Lifestyle changes for treating psoriasis. Cochrane Database of Systematic Reviews. 2019(7).
Korman N, Armour M, Chapman J, Rosenbaum S, Kisely S, Suetani S, et al. High Intensity Interval training (HIIT) for people with severe mental illness: A systematic review & meta-analysis of intervention studies- considering diverse approaches for mental and physical recovery. Psychiatry research. 2020;284:112601.
Kraal JJ, Vromen T, Spee R, Kemps HMC, Peek N. The influence of training characteristics on the effect of exercise training in patients with coronary artery disease: Systematic review and meta-regression analysis. International journal of cardiology. 2017;245:52-8.
Kruis AL, Smidt N, Assendelft WJ, Gussekloo J, Boland MR, Rutten-van Mölken M, et al. Integrated disease management interventions for patients with chronic obstructive pulmonary disease. Cochrane Database of Systematic Reviews. 2013(10):N.PAG-N.PAG.
Kuder MM, Clark M, Cooley C, Prieto-Centurion V, Danley A, Riley I, et al. A Systematic Review of the Effect of Physical Activity on Asthma Outcomes. J Allergy Clin Immunol Pract. 2021;9(9):3407-21.e8.
Kundakci B, Kaur J, Goh SL, Hall M, Doherty M, Zhang W, et al. Efficacy of nonpharmacological interventions for individual features of fibromyalgia: a systematic review and meta-analysis of randomised controlled trials. Pain. 2021.
Kuspinar A, Rodriguez AM, Mayo NE. The effects of clinical interventions on health-related quality of life in multiple sclerosis: A meta-analysis. Multiple Sclerosis Journal. 2012;18(12):1686-704.
Kwakkel G, de Goede CJT, van Wegen EEH. Impact of physical therapy for Parkinson's disease: A critical review of the literature. Parkinsonism & related disorders. 2007;13(Suppl 3):S478-S87.

Kwok JYY, Choi KC, Chan HYL. Effects of mind-body exercises on the physiological and psychosocial well-being of individuals with Parkinson's disease: A systematic review and meta-analysis. Complementary therapies in medicine. 2016;29:121-31.
La Touche R, Escalante K, Linares MT. Treating non-specific chronic low back pain through the Pilates Method. Journal of bodywork and movement therapies. 2008;12(4):364-70.
La Touche R, Fernandez Perez JJ, Proy Acosta A, Gonzalez Campodonico L, Martinez Garcia S, Adraos Juarez D, et al. Is aerobic exercise helpful in patients with migraine? A systematic review and meta-analysis. Scandinavian journal of medicine & science in sports. 2020;30(6):965-82.
Lacasse Y, Goldstein R, Lasserson TJ, Martin S. Pulmonary rehabilitation for chronic obstructive pulmonary disease. Cochrane Database of Systematic Reviews. 2015(2):N.PAG-N.PAG.
Lacasse Y, Martin S, Lasserson TJ, Goldstein RS. Meta-analysis of respiratory rehabilitation in chronic obstructive pulmonary disease: a Cochrane systematic review. Europa medicophysica. 2007;43(4):475-85.
Lahart IM, Metsios GS, Nevill AM, Carmichael AR. Physical activity for women with breast cancer after adjuvant therapy. The Cochrane database of systematic reviews. 2018;1:CD011292.
Lamotte G, Rafferty MR, Prodoehl J, Kohrt WM, Comella CL, Simuni T, et al. Effects of endurance exercise training on the motor and non-motor features of Parkinson's disease: A review. Journal of Parkinson's Disease. 2015;5(1):21-41.
Lane B, McCullagh R, Cardoso JR, McVeigh JG. The effectiveness of group and home-based exercise on psychological status in people with ankylosing spondylitis: A systematic review and meta-analysis. Musculoskeletal care. 2022.
Lange AK, Vanwanseele B, Fiatarone Singh MA. Strength training for treatment of osteoarthritis of the knee: a systematic review. Arthritis Rheum. 2008;59(10):1488-94.
Langer D, Hendriks EJM, Burtin C, Probst V, van der Schans CP, Paterson WJ, et al. A clinical practice guideline for physiotherapists treating patients with chronic obstructive pulmonary disease based on a systematic review of available evidence. Clinical rehabilitation. 2009;23(5):445-62.
Langhorst J, Klose P, Dobos GJ, Bernardy K, Hauser W. Efficacy and safety of meditative movement therapies in fibromyalgia syndrome: a systematic review and meta-analysis of randomized controlled trials. Rheumatology international. 2013;33(1):193-207.
Larsen RT, Gottliebsen CR, Wood KA, Risom SS. Lifestyle interventions after ablation for atrial fibrillation: a systematic review. European journal of cardiovascular nursing. 2020;19(7):564-79.
Larson JL, Vos CM, Fernandez D. Interventions to Increase Physical Activity in People With COPD. Annual review of nursing research. 2013;31:297-326.
Larun L, Odgaard-Jensen J, Price JR, Brurberg KG. An abridged version of the Cochrane review of exercise therapy for chronic fatigue syndrome. European journal of physical and rehabilitation medicine. 2016;52(2):244-52.
Lauche R, Hunter DJ, Adams J, Cramer H. Yoga for Osteoarthritis: a Systematic Review and Meta-analysis. Current rheumatology reports. 2019;21(9):47.
Lawlor ER, Bradley DT, Cupples ME, Tully MA. The effect of community-based interventions for cardiovascular disease secondary prevention on behavioural risk factors. Preventive Medicine: An International Journal Devoted to Practice and Theory. 2018;114:24-38.
Lawrence M, Celestino FT, Matozinho HHS, Govan L, Booth J, Beecher J. Yoga for stroke rehabilitation. Cochrane Database of Systematic Reviews. 2017;2017(12):CD011483.
Lawrence M, Celestino Junior FT, Matozinho HH, Govan L, Booth J, Beecher J. Yoga for stroke rehabilitation. The Cochrane database of systematic reviews. 2017;12:CD011483.
Lee AL, Hill CJ, McDonald CF, Holland AE. Pulmonary Rehabilitation in Individuals With Non-Cystic Fibrosis Bronchiectasis: A Systematic Review. Arch Phys Med Rehabil. 2017;98(4):774-82.e1.
Lee J. Physiologic and psychologic adaptation to exercise interventions in lung cancer patients undergoing chemotherapy: a systematic review and meta-analysis of randomized controlled trials. Supportive Care in Cancer. 2021;29(6):2863-73.
Lee J, Choi M, Yoo Y. A Meta-Analysis of Nonpharmacological Interventions for People With Parkinson's Disease. Clin Nurs Res. 2017;26(5):608-31.
Lee J, Lee MG. Effects of exercise interventions on breast cancer patients during adjuvant therapy: A systematic review and meta-analysis of randomized controlled trials. Cancer nursing. 2020;43(2):115-25.
Lee J, Stone AJ. Combined Aerobic and Resistance Training for Cardiorespiratory Fitness, Muscle Strength, and Walking Capacity after Stroke: A Systematic Review and Meta-Analysis. J Stroke Cerebrovasc Dis. 2020;29(1):104498.
Lee JE, Loh SY. Physical activity and quality of life of cancer survivors: a lack of focus for lifestyle redesign. Asian Pacific journal of cancer prevention : APJCP. 2013;14(4):2551-5.

Lee MS, Choi TY, Ernst E. Tai chi for breast cancer patients: a systematic review. Breast Cancer Res Treat. 2010;120(2):309-16.
Lee MS, Pittler MH, Ernst E. Tai chi for rheumatoid arthritis: Systematic review. Rheumatology. 2007;46(11):1648-51.
Leggio M, Fusco A, Coraci D, Villano A, Filardo G, Mazza A, et al. Exercise training and atrial fibrillation: a systematic review and literature analysis. European review for medical and pharmacological sciences. 2021;25(16):5163-75.
Leidy NK, Kimel M, Ajagbe L, Kim K, Hamilton A, Becker K. Designing trials of behavioral interventions to increase physical activity in patients with COPD: insights from the chronic disease literature. Respiratory medicine. 2014;108(3):472-81.
Leng M, Liang B, Zhou H, Zhang P, Hu M, Li G, et al. Effects of Physical Exercise on Depressive Symptoms in Patients With Cognitive Impairment: A Systematic Review and Meta-Analysis. The Journal of nervous and mental disease. 2018;206(10):809-23.
Lewinter C, Doherty P, Gale CP, Crouch S, Stirk L, Lewin RJ, et al. Exercise-based cardiac rehabilitation in patients with heart failure: a meta-analysis of randomised controlled trials between 1999 and 2013. Eur J Prev Cardiol. 2015;22(12):1504-12.
Li J, Gao X, Hao X, Kantas D, Mohamed EA, Zheng X, et al. Yoga for secondary prevention of coronary heart disease: A systematic review and meta-analysis. Complementary therapies in medicine. 2021;57:102643.
Li J, Guo NN, Jin HR, Yu H, Wang P, Xu GG. Effects of exercise training on patients with lung cancer who underwent lung resection: a meta-analysis. World J Surg Oncol. 2017;15(1):158.
Li L, Huang H, Song J, Yu Y, Jia Y, Wang Y, et al. Network Meta-Analysis of the Effects of Different Types of Traditional Chinese Exercises on Pulmonary Function, Endurance Capacity and Quality of Life in Patients With COPD. Front Med (Lausanne). 2022;9:806025.
Li N, Li P, Lu Y, Wang Z, Li J, Liu X, et al. Effects of resistance training on exercise capacity in elderly patients with chronic obstructive pulmonary disease: a meta-analysis and systematic review. Aging Clinical & Experimental Research. 2020;32(10):1911-22.
Li P, Li J, Wang Y, Xia J, Liu X. Effects of Exercise Intervention on Peripheral Skeletal Muscle in Stable Patients With COPD: A Systematic Review and Meta-Analysis. Front Med (Lausanne). 2021;8:766841.
Li R, Zhang Y, Jiang Y, Wang M, Ang WHD, Lau Y. Rehabilitation training based on virtual reality for patients with Parkinson's disease in improving balance, quality of life, activities of daily living, and depressive symptoms: A systematic review and meta-regression analysis. Clin Rehabil. 2021;35(8):1089-102.
Li W, Pu Y, Meng A, Zhi X, Xu G. Effectiveness of pulmonary rehabilitation in elderly patients with COPD: A systematic review and meta-analysis of randomized controlled trials. International Journal of Nursing Practice (John Wiley & Sons, Inc). 2019;25(5):N.PAG-N.PAG.
Li W-C, Chen Y-C, Yang R-S, Tsauo J-Y. Effects of exercise programmes on quality of life in osteoporotic and osteopenic postmenopausal women: A systematic review and meta-analysis. Clinical rehabilitation. 2009;23(10):888-96.
Li X, Gao Z, Yu H, Gu Y, Yang G. Effect of Long-term Exercise Therapy on Motor Symptoms in Parkinson Disease Patients: A Systematic Review and Meta-analysis of Randomized Controlled Trials. Am J Phys Med Rehabil. 2022;101(10):905-12.
Li X, Guo R, Wei Z, Jia J, Wei C. Effectiveness of Exercise Programs on Patients with Dementia: A Systematic Review and Meta-Analysis of Randomized Controlled Trials. BioMed research international. 2019;2019:2308475.
Li Y, Song H, Shen L, Wang Y. The efficacy and safety of moderate aerobic exercise for patients with Parkinson's disease: a systematic review and meta-analysis of randomized controlled trials. Ann Palliat Med. 2021;10(3):2638-49.
Li Y, Su Y, Chen S, Zhang Y, Zhang Z, Liu C, et al. The effects of resistance exercise in patients with knee osteoarthritis: a systematic review and meta-analysis. Clin Rehabil. 2016;30(10):947-59.
Lian Z, Yang L, Bian Y, Zeng L, Li M, Sun Y, et al. Effects of Tai chi on adults with essential hypertension in China: A systematic review and meta-analysis. European Journal of Integrative Medicine. 2017;12:153-62.
Liang H, Luo S, Chen X, Lu Y, Liu Z, Wei L. Effects of Tai Chi exercise on cardiovascular disease risk factors and quality of life in adults with essential hypertension: A meta-analysis. Heart Lung. 2020;49(4):353-63.
Liang H, Zhang H, Ji H, Wang C. Effects of home-based exercise intervention on health-related quality of life for patients with ankylosing spondylitis: a meta-analysis. Clinical rheumatology. 2015;34(10):1737-44.
Liang Z, Wang Q, Fu C, Liu R, Wang L, Pei G, et al. What conservative interventions can improve the long-term quality of life, depression, and anxiety of individuals with stable copd? A systematic review and

meta-analysis. Quality of Life Research: An International Journal of Quality of Life Aspects of Treatment, Care & Rehabilitation. 2021.
Liang Z, Wang Q, Fu C, Liu R, Wang L, Pei G, et al. What conservative interventions can improve the long-term quality of life, depression, and anxiety of individuals with stable COPD? A systematic review and meta-analysis. Quality of life research : an international journal of quality of life aspects of treatment, care and rehabilitation. 2022;31(4):977-89.
Liddle SD, Baxter GD, Gracey JH. Exercise and chronic low back pain: what works? Pain. 2004;107(1/2):176-90.
Lim ECW, Poh RLC, Low AY, Wong WP. Effects of Pilates-based exercises on pain and disability in individuals with persistent nonspecific low back pain: a systematic review with meta-analysis. The Journal of orthopaedic and sports physical therapy. 2011;41(2):70-80.
Lin KY, Frawley HC, Denehy L, Feil D, Granger CL. Exercise interventions for patients with gynaecological cancer: a systematic review and meta-analysis. Physiotherapy (United Kingdom). 2016;102(4):309-19.
Lipsett A, Barrett S, Haruna F, Mustian K, O'Donovan A. The impact of exercise during adjuvant radiotherapy for breast cancer on fatigue and quality of life: A systematic review and meta-analysis. Breast. 2017;32:144-55.
Littbrand H, Stenvall M, Rosendahl E. Applicability and effects of physical exercise on physical and cognitive functions and activities of daily living among people with dementia: a systematic review. American journal of physical medicine & rehabilitation. 2011;90(6):495-518.
Liu JX, Zhu L, Li PJ, Li N, Xu YB. Effectiveness of high-intensity interval training on glycemic control and cardiorespiratory fitness in patients with type 2 diabetes: a systematic review and meta-analysis. Aging Clin Exp Res. 2019;31(5):575-93.
Liu L, Dong H, Jin X, Brooke-Wavell K. Tackling Dementia: A Systematic Review of Interventions Based on Physical Activity. Journal of geriatric physical therapy (2001). 2021.
Liu L, He X, Feng L. Exercise on quality of life and cancer-related fatigue for lymphoma survivors: a systematic review and meta-analysis. Supportive Care in Cancer. 2019;27(11):4069-82.
Liu L, Tan H, Yu S, Yin H, Baxter GD. The effectiveness of tai chi in breast cancer patients: A systematic review and meta-analysis. Complement Ther Clin Pract. 2020;38:101078.
Liu T, Chan AW, Liu YH, Taylor-Piliae RE. Effects of Tai Chi-based cardiac rehabilitation on aerobic endurance, psychosocial well-being, and cardiovascular risk reduction among patients with coronary heart disease: A systematic review and meta-analysis. European journal of cardiovascular nursing. 2018;17(4):368-83.
Liu T, Sheng L, Huang Z. Influence of exercise intervention on pain, quality of life and functional mobility in patients with osteoporotic vertebral fractures: A systematic review and meta-analysis. International Journal of Clinical and Experimental Medicine. 2017;10(7):9864-70.
Liu X, Fu C, Hu W, Hao S, Xie L, Wu X, et al. The effect of Tai Chi on the pulmonary rehabilitation of chronic obstructive pulmonary disease: a systematic review and meta-analysis. Ann Palliat Med. 2021;10(4):3763-82.
Liu X-L, Tan J-Y, Wang T, Zhang Q, Zhang M, Yao L-Q, et al. Effectiveness of Home-Based Pulmonary Rehabilitation for Patients with Chronic Obstructive Pulmonary Disease: A Meta-Analysis of Randomized Controlled Trials. Rehabilitation Nursing. 2014;39(1):36-59.
Long A, Robinson K, Goldberg S, Gordon AL. Effectiveness of exercise interventions for adults over 65 with moderate-to-severe dementia in community settings: a systematic review. European Geriatric Medicine. 2019;10(6):843-52.
Long L, Anderson L, Dewhirst AM, He J, Bridges C, Gandhi M, et al. Exercise-based cardiac rehabilitation for adults with stable angina. Cochrane Database of Systematic Reviews. 2018(2).
Lopez P, Taaffe DR, Newton RU, Buffart LM, Galvao DA. What is the minimal dose for resistance exercise effectiveness in prostate cancer patients? Systematic review and meta-analysis on patient-reported outcomes. Prostate Cancer and Prostatic Diseases. 2021;24(2):465-81.
Loughney L, West MA, Kemp GJ, Grocott MP, Jack S. Exercise intervention in people with cancer undergoing neoadjuvant cancer treatment and surgery: A systematic review. Eur J Surg Oncol. 2016;42(1):28-38.
Luan L, Bousie J, Pranata A, Adams R, Han J. Stationary cycling exercise for knee osteoarthritis: A systematic review and meta-analysis. Clin Rehabil. 2021;35(4):522-33.
Luo L, Meng H, Wang Z, Zhu S, Yuan S, Wang Y, et al. Effect of high-intensity exercise on cardiorespiratory fitness in stroke survivors: A systematic review and meta-analysis. Ann Phys Rehabil Med. 2020;63(1):59-68.
Luo L, Zhu S, Shi L, Wang P, Li M, Yuan S. High Intensity Exercise for Walking Competency in Individuals with Stroke: A Systematic Review and Meta-Analysis. J Stroke Cerebrovasc Dis. 2019;28(12):104414.
Luo XC, Liu J, Fu J, Yin HY, Shen L, Liu ML, et al. Effect of Tai Chi Chuan in breast cancer patients: A systematic review and meta-analysis. Frontiers in Oncology. 2020;10:607.

Lyu X, Li S, Peng S, Cai H, Liu G, Ran X. Intensive walking exercise for lower extremity peripheral arterial disease: A systematic review and meta-analysis. J Diabetes. 2016;8(3):363-77.
Macedo LG, Maher CG, Latimer J, McAuley JH. Motor control exercise for persistent, nonspecific low back pain: a systematic review. Phys Ther. 2009;89(1):9-25.
Mackay-Lyons M, Thornton M, Ruggles T, Che M. Non-pharmacological interventions for preventing secondary vascular events after stroke or transient ischemic attack. The Cochrane database of systematic reviews. 2013(3):CD008656.
Marques MM, De Gucht V, Gouveia MJ, Leal I, Maes S. Differential effects of behavioral interventions with a graded physical activity component in patients suffering from Chronic Fatigue (Syndrome): An updated systematic review and meta-analysis. Clinical psychology review. 2015;40:123-37.
Martin H, Beard S, Clissold N, Andraos K, Currey L. Combined aerobic and resistance exercise interventions for individuals with schizophrenia: A systematic review. Mental Health and Physical Activity. 2017;12:147-55.
Martland R, Mondelli V, Gaughran F, Stubbs B. Can high intensity interval training improve health outcomes among people with mental illness? A systematic review and preliminary meta-analysis of intervention studies across a range of mental illnesses. Journal of affective disorders. 2020;263:629-60.
McGettigan M, Cardwell CR, Cantwell MM, Tully MA. Physical activity interventions for disease-related physical and mental health during and following treatment in people with non-advanced colorectal cancer. The Cochrane database of systematic reviews. 2020;5:CD012864.
McGregor G, Powell R, Kimani P, Underwood M. Does contemporary exercise-based cardiac rehabilitation improve quality of life for people with coronary artery disease? A systematic review and meta-analysis. BMJ open. 2020;10(6):e036089.
McMillan EM, Newhouse IJ. Exercise is an effective treatment modality for reducing cancer-related fatigue and improving physical capacity in cancer patients and survivors: a meta-analysis. Appl Physiol Nutr Metab. 2011;36(6):892-903.
McNamara RJ, McKeough ZJ, McKenzie DK, Alison JA. Water-based exercise training for chronic obstructive pulmonary disease. Cochrane Database of Systematic Reviews. 2013(12):N.PAG-N.PAG.
Meender Schäfer AG, Zalpour C, Piekartz Hv, Hall TM, Paelke V, Schäfer AGM, et al. The Efficacy of Electronic Health-Supported Home Exercise Interventions for Patients With Osteoarthritis of the Knee: Systematic Review. Journal of medical Internet research. 2018;20(4):1-.
Mehrholz J, Kugler J, Pohl M. Water-based exercises for improving activities of daily living after stroke. Cochrane Database of Systematic Reviews. 2011(1).
Mehrholz J, Kugler J, Storch A, Pohl M, Hirsch K, Elsner B. Treadmill training for patients with Parkinson's disease. Cochrane Database of Systematic Reviews. 2015(9).
Mehta S, Pereira S, Viana R, Mays R, McIntyre A, Janzen S, et al. Resistance Training for Gait Speed and Total Distance Walked During the Chronic Stage of Stroke: A Meta-Analysis. Topics in stroke rehabilitation. 2012;19(6):471-8.
Mendes Xavier D, Lanza Galvão E, Aliane Fonseca A, de Souza GM, Pereira Lima V. Effects of Home-Based Pulmonary Rehabilitation on Dyspnea, Exercise Capacity, Quality of Life and Impact of the Disease in COPD Patients: A Systematic Review. Copd. 2022;19(1):18-46.
Metsios GS, Stavropoulos-Kalinoglou A, Veldhuijzen van Zanten JJ, Treharne GJ, Panoulas VF, Douglas KM, et al. Rheumatoid arthritis, cardiovascular disease and physical exercise: a systematic review. Rheumatology (Oxford). 2008;47(3):239-48.
Michaelchuk W, Oliveira A, Marzolini S, Nonoyama M, Maybank A, Goldstein R, et al. Design and delivery of home-based telehealth pulmonary rehabilitation programs in COPD: A systematic review and meta-analysis. Int J Med Inform. 2022;162:104754.
Minns Lowe CJ, Barker KL, Dewey ME, Sackley CM. Effectiveness of physiotherapy exercise following hip arthroplasty for osteoarthritis: a systematic review of clinical trials. BMC Musculoskelet Disord. 2009;10:98.
Mira TAA, Buen MM, Borges MG, Yela DA, Benetti-Pinto CL, Benetti-Pinto CL. Systematic review and meta-analysis of complementary treatments for women with symptomatic endometriosis. International Journal of Gynecology & Obstetrics. 2018;143(1):2-9.
Miranda AdS, et al. Effects of walking and strength training on walking capacity in individuals with claudication: meta-analysis. Jornal Vascular Brasileiro. 2013;12(2):110-7.
Mishra SI, Aziz NM, Scherer RW, Baquet CR, Berlanstein DR, Geigle PM. Exercise interventions on health related quality of life for cancer survivors. Cochrane Database of Systematic Reviews. 2009(1):CD007566.
Mishra SI, Scherer RW, Geigle PM, Berlanstein DR, Topaloglu O, Gotay CC, et al. Exercise interventions on health-related quality of life for cancer survivors. Cochrane Database Syst Rev. 2012;2012(8):Cd007566.

Mishra SI, Scherer RW, Snyder C, Geigle P, Gotay C. The Effectiveness of Exercise Interventions for Improving Health-Related Quality of Life From Diagnosis Through Active Cancer Treatment. Oncology nursing forum. 2015;42(1):E33-E53.
Mishra SI, Scherer RW, Snyder C, Geigle PM, Berlanstein DR, Topaloglu O. Exercise interventions on health-related quality of life for people with cancer during active treatment. The Cochrane database of systematic reviews. 2012(8):CD008465.
Miyamoto GC, Costa LOP, Cabral CMN. Efficacy of the Pilates method for pain and disability in patients with chronic nonspecific low back pain: a systematic review with meta-analysis. Brazilian journal of physical therapy. 2013;17(6):517-32.
Mockel L. Potential benefits of yoga in patients with heart failure: A meta-analysis of controlled trials. European Journal of Integrative Medicine. 2019;31:100948.
Morris JH, Macgillivray S, McFarlane S. Interventions to promote long-term participation in physical activity after stroke: a systematic review of the literature. Arch Phys Med Rehabil. 2014;95(5):956-67.
Morris L, Stander J, Ebrahim W, Eksteen S, Meaden OA, Ras A, et al. Effect of exercise versus cognitive behavioural therapy or no intervention on anxiety, depression, fitness and quality of life in adults with previous methamphetamine dependency: a systematic review. Addiction science & clinical practice. 2018;13(1):4.
Mosalman Haghighi M, Mavros Y, Fiatarone Singh MA. The Effects of Structured Exercise or Lifestyle Behavior Interventions on Long-Term Physical Activity Level and Health Outcomes in Individuals With Type 2 Diabetes: A Systematic Review, Meta-Analysis, and Meta-Regression. Journal of physical activity & health. 2018;15(9):697-707.
Mudano AS, Tugwell P, Wells GA, Singh JA. Tai Chi for rheumatoid arthritis. Cochrane Database of Systematic Reviews. 2019(9).
Mura G, Moro MF, Patten SB, Carta MG. Exercise as an add-on strategy for the treatment of major depressive disorder: a systematic review. CNS Spectrums: The International Journal of Neuropsychiatric Medicine. 2014;19(6):496-508.
Murillo-Garcia A, Villafaina S, Adsuar JC, Gusi N, Collado-Mateo D. Effects of dance on pain in patients with fibromyalgia: A systematic review and meta-analysis. Evidence-based Complementary and Alternative Medicine. 2018;2018:8709748.
Natale P, Palmer S, Ruospo M, Hegbrant JB, Strippoli GF. Psychosocial interventions for preventing and treating depression in dialysis patients: A cochrane review and meta-analysis. Journal of the American Society of Nephrology. 2019;30:180.
Ng LWC, Ng DP, Wong WP. Is supervised exercise training safe in patients with anorexia nervosa? A meta-analysis. Physiotherapy. 2013;99(1):1-11.
Niesink A, Trappenburg JCA, de Weert-van Oene GH, Lammers JWJ, Verheij TJM, Schrijvers AJP. Systematic review of the effects of chronic disease management on quality-of-life in people with chronic obstructive pulmonary disease. Respiratory medicine. 2007;101(11):2233-9.
O'Shea SD, Taylor NF, Paratz JD. Progressive resistance exercise improves muscle strength and may improve elements of performance of daily activities for people with COPD: a systematic review. Chest. 2009;136(5):1269-83.
Oesterle A, Giancaterino S, Van Noord MG, Pellegrini CN, Fan D, Srivatsa UN, et al. Effects of Supervised Exercise Training on Atrial Fibrillation: A META-ANALYSIS OF RANDOMIZED CONTROLLED TRIALS. Journal of cardiopulmonary rehabilitation and prevention. 2022.
Ojagbemi A, Akin-Ojagbemi N. Exercise and Quality of Life in Dementia: A Systematic Review and Meta-Analysis of Randomized Controlled Trials. Journal of applied gerontology : the official journal of the Southern Gerontological Society. 2019;38(1):27-48.
Oliveira JLM, Galvão CM, Rocha SMM. Resistance exercises for health promotion in coronary patients: Evidence of benefits and risks. International journal of evidence-based healthcare. 2008;6(4):431-9.
Ora J, Prendi E, Attinà ML, Cazzola M, Calzetta L, Rogliani P. Efficacy of respiratory tele-rehabilitation in COPD patients: Systematic review and meta-analysis. Monaldi Arch Chest Dis. 2022;92(4).
Ostman C, Jewiss D, Smart NA. The Effect of Exercise Training Intensity on Quality of Life in Heart Failure Patients: A Systematic Review and Meta-Analysis. Cardiology. 2017;136(2):79-89.
Pacheco DR, Silva MJ, Alexandrino AM, Torres RM. Exercise-related quality of life in subjects with asthma: a systematic review. J Asthma. 2012;49(5):487-95.
Packer N, Hoffman-Goetz L, Ward G. Does physical activity affect quality of life, disease symptoms and immune measures in patients with inflammatory bowel disease? A systematic review. J Sports Med Phys Fitness. 2010;50(1):1-18.
Paixao C, Rocha V, Brooks D, Marques A. Unsupervised physical activity interventions for people with COPD: A systematic review and meta-analysis. Pulmonology. 2022.

Pan L, Yan J, Guo Y, Yan J. Effects of Tai Chi training on exercise capacity and quality of life in patients with chronic heart failure: a meta-analysis. European journal of heart failure. 2013;15(3):316-23.
Pan Y, Yang K, Shi X, Liang H, Zhang F, Lv Q. Tai chi chuan exercise for patients with breast cancer: a systematic review and meta-analysis. Evid Based Complement Alternat Med. 2015;2015:535237.
Pan Y, Yang K, Wang Y, Zhang L, Liang H. Could yoga practice improve treatment-related side effects and quality of life for women with breast cancer? A systematic review and meta-analysis. Asia Pac J Clin Oncol. 2017;13(2):e79-e95.
Pandey A, Garg S, Khunger M, Garg S, Kumbhani DJ, Chin KM, et al. Efficacy and Safety of Exercise Training in Chronic Pulmonary Hypertension: Systematic Review and Meta-Analysis. Circulation Heart failure. 2015;8(6):1032-43.
Panel P. Philadelphia panel evidence-based clinical practice guidelines on selected rehabilitation interventions for knee pain. Physical therapy. 2001;81(10):1675-700.
Paneroni M, Simonelli C, Vitacca M, Ambrosino N. Aerobic Exercise Training in Very Severe Chronic Obstructive Pulmonary Disease: A Systematic Review and Meta-Analysis. American journal of physical medicine & rehabilitation. 2017;96(8):541-8.
Pang MY, Charlesworth SA, Lau RW, Chung RC. Using aerobic exercise to improve health outcomes and quality of life in stroke: evidence-based exercise prescription recommendations. Cerebrovasc Dis. 2013;35(1):7-22.
Pape LM, Adriaanse MC, Kol J, van Straten A, van Meijel B. Patient-reported outcomes of lifestyle interventions in patients with severe mental illness: a systematic review and meta-analysis. BMC psychiatry. 2022;22(1):261.
Paramanandam VS, Roberts D. Weight training is not harmful for women with breast cancer-related lymphoedema: a systematic review. J Physiother. 2014;60(3):136-43.
Parmenter B, Dieberg G, Smart N. Exercise Training for Management of Peripheral Arterial Disease: A Systematic Review and Meta-Analysis. Sports Medicine. 2015;45(2):231-44.
Patten RK, Boyle RA, Moholdt T, Kiel I, Hopkins WG, Harrison CL, et al. Exercise Interventions in Polycystic Ovary Syndrome: A Systematic Review and Meta-Analysis. Frontiers in Physiology. 2020;11:606.
Patten RK, Pascoe MC, Moreno-Asso A, Boyle RA, Stepto NK, Parker AG. Effectiveness of exercise interventions on mental health and health-related quality of life in women with polycystic ovary syndrome: a systematic review. BMC public health. 2021;21(1):2310.
Pearsall R, Smith DJ, Pelosi A, Geddes J. Exercise therapy in adults with serious mental illness: a systematic review and meta-analysis. BMC psychiatry. 2014;14:117.
Peddle-McIntyre CJ, Singh F, Thomas R, Newton RU, Galvao DA, Cavalheri V. Exercise training for advanced lung cancer. Cochrane Database of Systematic Reviews. 2019;2019(2):CD012685.
Peng S, He J, Huang J, Lun L, Zeng J, Zeng S, et al. Self-management interventions for chronic kidney disease: a systematic review and meta-analysis. BMC nephrology. 2019;20(1):N.PAG-N.PAG.
Persoon S, Kersten MJ, van der Weiden K, Buffart LM, Nollet F, Brug J, et al. Effects of exercise in patients treated with stem cell transplantation for a hematologic malignancy: a systematic review and meta-analysis. Cancer Treat Rev. 2013;39(6):682-90.
Peurala SH, Karttunen AH, Sjögren T, Paltamaa J, Heinonen A. Evidence for the effectiveness of walking training on walking and self-care after stroke: a systematic review and meta-analysis of randomized controlled trials. J Rehabil Med. 2014;46(5):387-99.
Peytremann-Bridevaux I, Staeger P, Bridevaux P-O, Ghali WA, Burnand B. Effectiveness of chronic obstructive pulmonary disease-management programs: systematic review and meta-analysis. The American journal of medicine. 2008;121(5):433-43.e4.
Pigott JS, Kane EJ, Ambler G, Walters K, Schrag A. Systematic review and meta-analysis of clinical effectiveness of self-management interventions in Parkinson's disease. BMC Geriatr. 2022;22(1):45.
Pinto-Carral A, Molina AJ, de Pedro Á, Ayán C. Pilates for women with breast cancer: A systematic review and meta-analysis. Complement Ther Med. 2018;41:130-40.
Polese JC, Ada L, Dean CM, Nascimento LR, Teixeira-Salmela LF. Treadmill training is effective for ambulatory adults with stroke: a systematic review. J Physiother. 2013;59(2):73-80.
Pollock A, Baer G, Campbell P, Choo PL, Forster A, Morris J, et al. Physical rehabilitation approaches for the recovery of function and mobility following stroke. Cochrane Database of Systematic Reviews. 2014(4).
Pollock A, Gray C, Culham E, Durward BR, Langhorne P. Interventions for improving sit-to-stand ability following stroke. Cochrane Database of Systematic Reviews. 2014(5).
Ponzano M, Tibert N, Bansal S, Katzman W, Giangregorio L. Exercise for improving age-related hyperkyphosis: a systematic review and meta-analysis with GRADE assessment. Archives of osteoporosis. 2021;16(1):140.

Powell R, McGregor G, Ennis S, Kimani PK, Underwood M. Is exercise-based cardiac rehabilitation effective? A systematic review and meta-Analysis to re-examine the evidence. BMJ open. 2018;8(3):e019656.
Pu J, Jiang Z, Wu W, Li L, Zhang L, Li Y, et al. Efficacy and safety of intradialytic exercise in haemodialysis patients: a systematic review and meta-analysis. BMJ open. 2019;9(1):e020633.
Puhan MA, Gimeno-Santos E, Scharplatz M, Troosters T, Walters EH, Steurer J. Pulmonary rehabilitation following exacerbations of chronic obstructive pulmonary disease. Cochrane Database of Systematic Reviews. 2011:N.PAG-N.PAG.
Qin J, Zhao K, Chen Y, Guo S, You Y, Xie J, et al. The Effects of Exercise Interventions on Balance Capacity in Patients with Type 2 Diabetes Mellitus: A Systematic Review and Meta-Analysis. Inquiry. 2021;58:469580211018284.
Rackwitz B, de Bie R, Limm H, von Garnier K, Ewert T, Stucki G. Segmental stabilizing exercises and low back pain. What is the evidence? A systematic review of randomized controlled trials. Clinical rehabilitation. 2006;20(7):553-67.
Ramachandran HJ, Jiang Y, Tam WWS, Yeo TJ, Wang W. Effectiveness of home-based cardiac telerehabilitation as an alternative to Phase 2 cardiac rehabilitation of coronary heart disease: a systematic review and meta-analysis. European journal of preventive cardiology. 2021.
Ramirez-velez R, Zambom-ferraresi F, Garcia-hermoso A, Kievisiene J, Rauckiene-michealsson A, Agostinis-sobrinho C. Evidence-based exercise recommendations to improve mental wellbeing in women with breast cancer during active treatment: A systematic review and meta-analysis. Cancers. 2021;13(2):1-27.
Rammant E, Decaestecker K, Bultijnck R, Sundahl N, Ost P, Pauwels NS, et al. A systematic review of exercise and psychosocial rehabilitation interventions to improve health-related outcomes in patients with bladder cancer undergoing radical cystectomy. Clinical rehabilitation. 2018;32(5):594-606.
Ran G, Yi W, Ying X, Ruikun J, Song Z, Sijie L, et al. The impact of exercise training for chronic heart failure patients with cardiac resynchronization therapy: A systematic review and meta-analysis. Medicine. 2021;100(13):1-10.
Rao AK, Chou A, Bursley B, Smulofsky J, Jezequel J. Systematic review of the effects of exercise on activities of daily living in people with Alzheimer's disease. Am J Occup Ther. 2014;68(1):50-6.
Raposo F, Ramos M, Lúcia Cruz A. Effects of exercise on knee osteoarthritis: A systematic review. Musculoskeletal Care. 2021;19(4):399-435.
Reed JL, Mark AE, Reid RD, Pipe AL. The effects of chronic exercise training in individuals with permanent atrial fibrillation: a systematic review. The Canadian journal of cardiology. 2013;29(12):1721-8.
Reed JL, Terada T, Chirico D, Prince SA, Pipe AL. The Effects of Cardiac Rehabilitation in Patients With Atrial Fibrillation: A Systematic Review. The Canadian journal of cardiology. 2018;34(10 Suppl 2):S284-S95.
Rees K, Taylor RS, Singh S, Coats AJS, Ebrahim S. Exercise based rehabilitation for heart failure. The Cochrane database of systematic reviews. 2004(3):CD003331.
Regan EW, Handlery R, Beets MW, Fritz SL. Are Aerobic Programs Similar in Design to Cardiac Rehabilitation Beneficial for Survivors of Stroke? A Systematic Review and Meta-Analysis. J Am Heart Assoc. 2019;8(16):e012761.
Reger M, Kutschan S, Freuding M, Schmidt T, Josfeld L, Huebner J. Water therapies (hydrotherapy, balneotherapy or aqua therapy) for patients with cancer: a systematic review. Journal of Cancer Research and Clinical Oncology. 2022;148(6):1277-97.
Reina-Gutiérrez S, Torres-Costoso A, Martínez-Vizcaíno V, Núñez de Arenas-Arroyo S, Fernández-Rodríguez R, Pozuelo-Carrascosa DP. Effectiveness of Pulmonary Rehabilitation in Interstitial Lung Disease, Including Coronavirus Diseases: A Systematic Review and Meta-analysis. Arch Phys Med Rehabil. 2021;102(10):1989-97.e3.
Reiter BD, Arora RR. The role of exercise in heart failure: a systematic review. American journal of therapeutics. 2014;21(5):403-11.
Ren J, Xia J. Dance therapy for schizophrenia. Cochrane Database of Systematic Reviews. 2013;2013(10):CD006868.
Rietberg MB, Brooks D, Uitdehaag BMJ, Kwakkel G. Exercise therapy for multiple sclerosis. Cochrane Database of Systematic Reviews. 2005(1).
Risom SS, Zwisler A-D, Johansen PP, Sibilitz KL, Lindschou J, Gluud C, et al. Exercise-based cardiac rehabilitation for adults with atrial fibrillation. The Cochrane database of systematic reviews. 2017;2:CD011197.
Roberts MT, Lloyd J, Valimaki M, Ho GW, Freemantle M, Bekefi AZ. Video games for people with schizophrenia. The Cochrane database of systematic reviews. 2021;2:CD012844.
Roddy E, Zhang W, Doherty M. Aerobic walking or strengthening exercise for osteoarthritis of the knee? A systematic review. Ann Rheum Dis. 2005;64(4):544-8.

Rooney S, Webster A, Paul L. Systematic Review of Changes and Recovery in Physical Function and Fitness After Severe Acute Respiratory Syndrome–Related Coronavirus Infection: Implications for COVID-19 Rehabilitation. Physical therapy. 2020;100(10):1717-29.
Rubaek M, Hitz MF, Holmberg T, Schonwandt BMT, Andersen S. Effectiveness of patient education for patients with osteoporosis: a systematic review. Osteoporosis International. 2021.
Rueda JR, Sola I, Pascual A, Subirana Casacuberta M. Non-invasive interventions for improving well-being and quality of life in patients with lung cancer. Cochrane Database of Systematic Reviews. 2011;2011(9):CD004282.
Ruiz-Muelle A, López-Rodríguez MM. Dance for people with Alzheimer’s disease: A systematic-review. Current Alzheimer research. 2019;16(10):919-34.
Russ J, Weyh C, Pilat C. High-intensity exercise programs in people with dementia — a systematic review and meta-analysis. German Journal of Exercise and Sport Research. 2021;51(1):4-16.
Rutledge T, Redwine LS, Linke SE, Mills PJ. A meta-analysis of mental health treatments and cardiac rehabilitation for improving clinical outcomes and depression among patients with coronary heart disease. Psychosomatic medicine. 2013;75(4):335-49.
Sagar VA, Davies EJ, Briscoe S, Coats AJ, Dalal HM, Lough F, et al. Exercise-based rehabilitation for heart failure: systematic review and meta-analysis. Open Heart. 2015;2(1):e000163.
Salam A, Woodman A, Chu A, Al-Jamea LH, Islam M, Sagher M, et al. Effect of post-diagnosis exercise on depression symptoms, physical functioning and mortality in breast cancer survivors: A systematic review and meta-analysis of randomized control trials. Cancer epidemiology. 2022;77:102111.
Salse-Batán J, Sanchez-Lastra MA, Suarez-Iglesias D, Varela S, Ayán C. Effects of Nordic walking in people with Parkinson's disease: A systematic review and meta-analysis. Health Soc Care Community. 2022;30(5):e1505-e20.
Salter K, Musovic A, N FT. In the first 3 months after stroke is progressive resistance training safe and does it improve activity? A systematic review. Top Stroke Rehabil. 2016;23(5):366-75.
Saltychev M, Sjögren T, Bärlund E, Laimi K, Paltamaa J. Do aerobic exercises really improve aerobic capacity of stroke survivors? A systematic review and meta-analysis. Eur J Phys Rehabil Med. 2016;52(2):233-43.
Salzwedel A, Jensen K, Rauch B, Doherty P, Metzendorf MI, Hackbusch M, et al. Effectiveness of comprehensive cardiac rehabilitation in coronary artery disease patients treated according to contemporary evidence based medicine: Update of the Cardiac Rehabilitation Outcome Study (CROS-II). European journal of preventive cardiology. 2020;27(16):1756-74.
Sanchez-Lastra MA, Martinez-Aldao D, Molina AJ, Ayan C. Pilates for people with multiple sclerosis: A systematic review and meta-analysis. Multiple sclerosis and related disorders. 2019;28:199-212.
Santos EJF, Duarte C, Marques A, Cardoso D, Apóstolo J, da Silva JAP, et al. Effectiveness of non-pharmacological and non-surgical interventions for rheumatoid arthritis: an umbrella review. JBI Database System Rev Implement Rep. 2019;17(7):1494-531.
Santos-Lozano A, Sanchis-Gomar F, Barrero-Santalla S, Pareja-Galeano H, Cristi-Montero C, Sanz-Ayan P, et al. Exercise as an adjuvant therapy against chronic atrial fibrillation. International journal of cardiology. 2016;207:180-4.
Saueressig T, Owen PJ, Zebisch J, Herbst M, Belavy DL. Evaluation of Exercise Interventions and Outcomes After Hip Arthroplasty: A Systematic Review and Meta-analysis. JAMA Netw Open. 2021;4(2):e210254.
Saunders DH, Mead GE, Fitzsimons C, Kelly P, van Wijck F, Verschuren O, et al. Interventions for reducing sedentary behaviour in people with stroke. Cochrane Database of Systematic Reviews. 2021;2021(6):CD012996.
Saunders DH, Sanderson M, Brazzelli M, Greig CA, Mead GE. Physical fitness training for stroke patients. Cochrane Database Syst Rev. 2013(10):Cd003316.
Scapini KB, Bohlke M, Moraes OA, Rodrigues CG, Inacio JF, Sbruzzi G, et al. Combined training is the most effective training modality to improve aerobic capacity and blood pressure control in people requiring haemodialysis for end-stage renal disease: systematic review and network meta-analysis. Journal of physiotherapy. 2019;65(1):4-15.
Schaafsma F, Schonstein E, Whelan KM, Ulvestad E, Kenny DT, Verbeek JH. Physical conditioning as part of a return to work strategy to reduce sickness absence for workers with back pain. Cochrane Database of Systematic Reviews. 2013(8):N.PAG-N.PAG.
Schellenberg ES, Dryden DM, Vandermeer B, Ha C, Korownyk C. Lifestyle interventions for patients with and at risk for type 2 diabetes: a systematic review and meta-analysis. Ann Intern Med. 2013;159(8):543-51.

Schmitz KH, Holtzman J, Courneya KS, Masse LC, Duval S, Kane R. Controlled physical activity trials in cancer survivors: A systematic review and meta-analysis. Cancer Epidemiology Biomarkers and Prevention. 2005;14(7):1588-95.
Schootemeijer S, van der Kolk NM, Bloem BR, de Vries NM. Current Perspectives on Aerobic Exercise in People with Parkinson's Disease. Neurotherapeutics. 2020;17(4):1418-33.
Schumann D, Anheyer D, Lauche R, Dobos G, Langhorst J, Cramer H. Effect of Yoga in the Therapy of Irritable Bowel Syndrome: A Systematic Review. Clinical Gastroenterology and Hepatology. 2016;14(12):1720-31.
Scott I, Cooper C, Leverton M, Burton A, Beresford-Dent J, Rockwood K, et al. Effects of nonpharmacological interventions on functioning of people living with dementia at home: A systematic review of randomised controlled trials. International journal of geriatric psychiatry. 2019;34(10):1386-402.
Semlitsch T, Jeitler K, Hemkens LG, Horvath K, Nagele E, Schuermann C, et al. Increasing physical activity for the treatment of hypertension: a systematic review and meta-analysis. Sports medicine (Auckland, NZ). 2013;43(10):1009-23.
Shawahna R, Batta A, Asa'ad M, Jomaah M, Abdelhaq I. Exercise as a complementary medicine intervention in type 2 diabetes mellitus: A systematic review with narrative and qualitative synthesis of evidence. Diabetes Metab Syndr. 2021;15(1):273-86.
Shen X, Wong-Yu IS, Mak MK. Effects of Exercise on Falls, Balance, and Gait Ability in Parkinson's Disease: A Meta-analysis. Neurorehabil Neural Repair. 2016;30(6):512-27.
Shepherd CW, While AE. Cardiac rehabilitation and quality of life: A systematic review. International journal of nursing studies. 2012;49(6):755-71.
Shibuya M, Yamamoto S, Kobayashi S, Nishie K, Yamaga T, Kawachi S, et al. Pulmonary Rehabilitation for Patients After COPD Exacerbation. Respir Care. 2022;67(3):360-9.
Siegel P, Tencza M, Apodaca B, Poole JL. Effectiveness of Occupational Therapy Interventions for Adults With Rheumatoid Arthritis: A Systematic Review. The American journal of occupational therapy : official publication of the American Occupational Therapy Association. 2017;71(1):7101180050p1-p11.
Sin DD, McAlister FA, Man SFP, Anthonisen NR, Sin DD, McAlister FA, et al. Contemporary management of chronic obstructive pulmonary disease: scientific review. JAMA: Journal of the American Medical Association. 2003;290(17):2301-12.
Singh B, Spence R, Steele ML, Hayes S, Toohey K. Exercise for Individuals With Lung Cancer: A Systematic Review and Meta-Analysis of Adverse Events, Feasibility, and Effectiveness. Seminars in oncology nursing. 2020;36(5):151076.
Singh B, Spence RR, Steele ML, Sandler CX, Peake JM, Hayes SC. A Systematic Review and Meta-Analysis of the Safety, Feasibility, and Effect of Exercise in Women With Stage II+ Breast Cancer. Archives of physical medicine and rehabilitation. 2018;99(12):2621-36.
Smart NA, Dieberg G, Giallauria F. Intermittent versus continuous exercise training in chronic heart failure: a meta-analysis. International journal of cardiology. 2013;166(2):352-8.
Smart NA, King N, Lambert JD, Pearson MJ, Campbell JL, Risom SS, et al. Exercise-based cardiac rehabilitation improves exercise capacity and health-related quality of life in people with atrial fibrillation: A systematic review and meta-analysis of randomised and non-randomised trials. Open heart. 2018;5(2):e000880.
Smeets RJEM, Wade D, Hidding A, Van Leeuwen PJCM, Vlaeyen JWS, Knottnerus JA. The association of physical deconditioning and chronic low back pain: a hypothesis-oriented systematic review. Disability and rehabilitation. 2006;28(11):673-93.
Smith MEB, Haney E, McDonagh M, Pappas M, Daeges M, Wasson N, et al. Treatment of Myalgic Encephalomyelitis/Chronic Fatigue Syndrome: A Systematic Review for a National Institutes of Health Pathways to Prevention Workshop. Annals of internal medicine. 2015;162(12):841-50.
Song R, Grabowska W, Park M, Osypiuk K, Vergara-Diaz GP, Bonato P, et al. The impact of Tai Chi and Qigong mind-body exercises on motor and non-motor function and quality of life in Parkinson's disease: A systematic review and meta-analysis. Parkinsonism and Related Disorders. 2017;41:3-13.
Soril LJJ, Damant RW, Lam GY, Smith MP, Weatherald J, Bourbeau J, et al. The effectiveness of pulmonary rehabilitation for Post-COVID symptoms: A rapid review of the literature. Respiratory medicine. 2022;195:106782.
Sosa-Reina MD, Nunez-Nagy S, Gallego-Izquierdo T, Pecos-Martin D, Monserrat J, Alvarez-Mon M. Effectiveness of Therapeutic Exercise in Fibromyalgia Syndrome: A Systematic Review and Meta-Analysis of Randomized Clinical Trials. BioMed research international. 2017;2017:2356346.
Spence RR, Heesch KC, Brown WJ. Exercise and cancer rehabilitation: a systematic review. Cancer treatment reviews. 2010;36(2):185-94.
Spruit MA, Eterman RM, Hellwig VA, Janssen PP, Wouters EF, Uszko-Lencer NH. Effects of moderate-to-high intensity resistance training in patients with chronic heart failure. Heart. 2009;95(17):1399-408.

Stafinski T, Nagase FI, Avdagovska M, Stickland MK, Menon D. Effectiveness of home-based pulmonary rehabilitation programs for patients with chronic obstructive pulmonary disease (COPD): systematic review. BMC Health Serv Res. 2022;22(1):557.
States RA, Pappas E, Salem Y. Overground physical therapy gait training for chronic stroke patients with mobility deficits. Cochrane Database of Systematic Reviews. 2009(3):CD006075.
Stoller O, de Bruin ED, Knols RH, Hunt KJ. Effects of cardiovascular exercise early after stroke: systematic review and meta-analysis. BMC Neurol. 2012;12:45.
Strasser B, Steindorf K, Wiskemann J, Ulrich CM. Impact of resistance training in cancer survivors: a meta-analysis. Med Sci Sports Exerc. 2013;45(11):2080-90.
Su JJ, Yu DSF, Paguio JT. Effect of eHealth cardiac rehabilitation on health outcomes of coronary heart disease patients: A systematic review and meta-analysis. Journal of advanced nursing. 2020;76(3):754-72.
Suárez-Iglesias D, Santos L, Sanchez-Lastra MA, Ayán C. Systematic review and meta-analysis of randomised controlled trials on the effects of yoga in people with Parkinson's disease. Disabil Rehabil. 2022;44(21):6210-29.
Sultana M, Bryant D, Orange JB, Beedie T, Montero-Odasso M. Effect of Wii Fit© Exercise on Balance of Older Adults with Neurocognitive Disorders: A Meta-Analysis. J Alzheimers Dis. 2020;75(3):817-26.
Swartz MC, Lewis ZH, Lyons EJ, Jennings K, Middleton A, Deer RR, et al. Effect of Home- and Community-Based Physical Activity Interventions on Physical Function Among Cancer Survivors: A Systematic Review and Meta-Analysis. Arch Phys Med Rehabil. 2017;98(8):1652-65.
Tamin TZ, Murdana N, Pitoyo Y, Safitri ED. Exercise Intervention for Chronic Pain Management, Muscle Strengthening, and Functional Score in Obese Patients with Chronic Musculoskeletal Pain: A Systematic Review and Meta-analysis. Acta medica Indonesiana. 2018;50(4):299-308.
Tanaka R, Ozawa J, Kito N, Yamasaki T, Moriyama H. Evidence of Improvement in Various Impairments by Exercise Interventions in Patients with Knee Osteoarthritis: A Systematic Review and Meta-analysis of Randomized Clinical Trials. J Jpn Phys Ther Assoc. 2013;16(1):7-21.
Taylor D, Jenkins AR, Parrott K, Benham A, Targett S, Jones AW. Efficacy of unsupervised exercise in adults with obstructive lung disease: a systematic review and meta-analysis. Thorax. 2021;76(6):591-600.
Taylor RS, Brown A, Ebrahim S, Jolliffe J, Noorani H, Rees K, et al. Exercise-based rehabilitation for patients with coronary heart disease: systematic review and meta-analysis of randomized controlled trials. The American journal of medicine. 2004;116(10):682-92.
Taylor RS, Sagar VA, Davies EJ, Briscoe S, Coats AJS, Dalal H, et al. Exercise-based rehabilitation for heart failure. The Cochrane database of systematic reviews. 2014(4):CD003331.
Taylor RS, Walker S, Ciani O, Warren F, Smart NA, Piepoli M, et al. Exercise-based cardiac rehabilitation for chronic heart failure: the EXTRAMATCH II individual participant data meta-analysis. Health technology assessment (Winchester, England). 2019;23(25):1-98.
Taylor-Piliae R, Finley BA. Benefits of Tai Chi Exercise Among Adults With Chronic Heart Failure: A Systematic Review and Meta-Analysis. The Journal of cardiovascular nursing. 2020;35(5):423-34.
Teleni L, Chan RJ, Chan A, Isenring EA, Vela I, Inder WJ, et al. Exercise improves quality of life in androgen deprivation therapytreated prostate cancer: Systematic review of randomised controlled trials. Endocrine-Related Cancer. 2016;23(2):101-12.
Tennigkeit J, Feige T, Haak M, Hellqvist C, Seven US, Kalbe E, et al. Structured care and self-management education for persons with parkinson's disease: Why the first does not go without the second-systematic review, experiences and implementation concepts from sweden and germany. Journal of Clinical Medicine. 2020;9(9):1-34.
ter Hoeve N, Huisstede BM, Stam HJ, van Domburg RT, Sunamura M, van den Berg-Emons RJ. Does cardiac rehabilitation after an acute cardiac syndrome lead to changes in physical activity habits? Systematic review. Phys Ther. 2015;95(2):167-79.
Thangarasa T, Imtiaz R, Hiremath S, Zimmerman D. Physical Activity in Patients Treated With Peritoneal Dialysis: A Systematic Review and Meta-analysis. Canadian Journal of Kidney Health and Disease. 2018;5.
Thayabaranathan T, Andrew NE, Immink MA, Hillier S, Stevens P, Stolwyk R, et al. Determining the potential benefits of yoga in chronic stroke care: a systematic review and meta-analysis. Top Stroke Rehabil. 2017;24(4):279-87.
Tiihonen M, Westner BU, Butz M, Dalal SS. Parkinson's disease patients benefit from bicycling - a systematic review and meta-analysis. NPJ Parkinsons Dis. 2021;7(1):86.
Tomlinson CL, Patel S, Meek C, Herd CP, Clarke CE, Stowe R, et al. Physiotherapy versus placebo or no intervention in Parkinson's disease. Cochrane Database of Systematic Reviews. 2013(9).
Tong H, Liu Y, Zhu Y, Zhang B, Hu J. The therapeutic effects of qigong in patients with chronic obstructive pulmonary disease in the stable stage: a meta-analysis. BMC Complementary & Alternative Medicine. 2019;19(1):N.PAG-N.PAG.

Tsang A, Lynes D, McKenzie H, Spencer S, Kelly C. Self-management programmes for adult patients with bronchiectasis: a systematic review and realist synthesis. Disability and rehabilitation. 2021:1-10.
Turner MN, Hernandez DO, Cade W, Emerson CP, Reynolds JM, Best TM. The Role of Resistance Training Dosing on Pain and Physical Function in Individuals With Knee Osteoarthritis: A Systematic Review. Sports Health. 2020;12(2):200-6.
Turner RR, Steed L, Quirk H, Greasley RU, Saxton JM, Taylor SJ, et al. Interventions for promoting habitual exercise in people living with and beyond cancer. Cochrane Database Syst Rev. 2018;9(9):Cd010192.
Vaikuntharajan P, Tobis M, Richardson J. Telephone-Delivered Physiotherapy Interventions Improve Physical Function for Adults With a Chronic Condition: A Systematic Review and Meta-analysis. Archives of physical medicine and rehabilitation. 2022;103(1):131-44.e14.
Vainshelboim B, Fox BD, Oliveira J, Kramer MR. Exercise training in idiopathic pulmonary fibrosis. Expert Rev Respir Med. 2016;10(1):69-77.
van de Port IG, Wood-Dauphinee S, Lindeman E, Kwakkel G. Effects of exercise training programs on walking competency after stroke: a systematic review. Am J Phys Med Rehabil. 2007;86(11):935-51.
van der Heijden MMP, van Dooren FEP, Pop VJM, Pouwer F. Effects of exercise training on quality of life, symptoms of depression, symptoms of anxiety and emotional well-being in type 2 diabetes mellitus: a systematic review. Diabetologia. 2013;56(6):1210-25.
van der Meer S, Zwerink M, van Brussel M, van der Valk P, Wajon E, van der Palen J. Effect of outpatient exercise training programmes in patients with chronic heart failure: a systematic review. European journal of preventive cardiology. 2012;19(4):795-803.
van Duijnhoven HJ, Heeren A, Peters MA, Veerbeek JM, Kwakkel G, Geurts AC, et al. Effects of Exercise Therapy on Balance Capacity in Chronic Stroke: Systematic Review and Meta-Analysis. Stroke. 2016;47(10):2603-10.
van Haren IE, Timmerman H, Potting CM, Blijlevens NM, Staal JB, Nijhuis-van der Sanden MW. Physical exercise for patients undergoing hematopoietic stem cell transplantation: systematic review and meta-analyses of randomized controlled trials. Phys Ther. 2013;93(4):514-28.
van Tol BA, Huijsmans RJ, Kroon DW, Schothorst M, Kwakkel G. Effects of exercise training on cardiac performance, exercise capacity and quality of life in patients with heart failure: a meta-analysis. Eur J Heart Fail. 2006;8(8):841-50.
Van Tulder M, Malmivaara A, Esmail R, Koes B. Exercise therapy for low back pain: A systematic review within the framework of the cochrane collaboration back review group. Spine. 2000;25(21):2784-96.
van Weert E, Hoekstra-Weebers JE, May AM, Korstjens I, Ros WJ, van der Schans CP. The development of an evidence-based physical self-management rehabilitation programme for cancer survivors. Patient Education & Counseling. 2008;71(2):169-90.
Vancampfort D, Probst M, Helvik Skjaerven L, Catalan-Matamoros D, Lundvik-Gyllensten A, Gomez-Conesa A, et al. Systematic review of the benefits of physical therapy within a multidisciplinary care approach for people with schizophrenia. Physical therapy. 2012;92(1):11-23.
Vancampfort D, Rosenbaum S, Schuch F, Ward PB, Richards J, Mugisha J, et al. Cardiorespiratory Fitness in Severe Mental Illness: A Systematic Review and Meta-analysis. Sports medicine (Auckland, NZ). 2017;47(2):343-52.
Vancampfort D, Rosenbaum S, Ward PB, Stubbs B. Exercise improves cardiorespiratory fitness in people with schizophrenia: A systematic review and meta-analysis. Schizophr Res. 2015;169(1-3):453-7.
Vancampfort D, Vanderlinden J, De Hert M, Soundy A, Adámkova M, Skjaerven LH, et al. A systematic review of physical therapy interventions for patients with anorexia and bulemia nervosa. Disability and Rehabilitation: An International, Multidisciplinary Journal. 2014;36(8):628-34.
Vancampfort D, Vansteelandt K, Scheewe T, Probst M, Knapen J, De Herdt A, et al. Yoga in schizophrenia: a systematic review of randomised controlled trials. Acta psychiatrica Scandinavica. 2012;126(1):12-20.
Vashistha V, Singh B, Kaur S, Prokop LJ, Kaushik D. The Effects of Exercise on Fatigue, Quality of Life, and Psychological Function for Men with Prostate Cancer: Systematic Review and Meta-analyses. European Urology Focus. 2016;2(3):284-95.
Veldema J, Jansen P. Aquatic therapy in stroke rehabilitation: systematic review and meta-analysis. Acta Neurol Scand. 2021;143(3):221-41.
Vellata C, Belli S, Balsamo F, Giordano A, Colombo R, Maggioni G. Effectiveness of Telerehabilitation on Motor Impairments, Non-motor Symptoms and Compliance in Patients With Parkinson's Disease: A Systematic Review. Front Neurol. 2021;12:627999.
Vera-Garcia E, Mayoral-Cleries F, Vancampfort D, Stubbs B, Cuesta-Vargas AI. A systematic review of the benefits of physical therapy within a multidisciplinary care approach for people with schizophrenia: An update. Psychiatry research. 2015;229(3):828-39.

Verschueren S, Eskes AM, Maaskant JM, Roest AM, Latour CHM, Op Reimer WS. The effect of exercise therapy on depressive and anxious symptoms in patients with ischemic heart disease: A systematic review. Journal of psychosomatic research. 2018;105:80-91.
Vieira DS, Maltais F, Bourbeau J. Home-based pulmonary rehabilitation in chronic obstructive pulmonary disease patients. Curr Opin Pulm Med. 2010;16(2):134-43.
Vilarino GT, Andreato LV, de Souza LC, Branco JHL, Andrade A. Effects of resistance training on the mental health of patients with fibromyalgia: a systematic review. Clinical rheumatology. 2021;40(11):4417-25.
Vooijs M, Siemonsma PC, Heus I, Sont JK, Rövekamp TAJM, van Meeteren NLU. Therapeutic validity and effectiveness of supervised physical exercise training on exercise capacity in patients with chronic obstructive pulmonary disease: a systematic review and meta-analysis. Clinical rehabilitation. 2016;30(11):1037-48.
Vromen T, Kraal JJ, Kuiper J, Spee RF, Peek N, Kemps HM. The influence of training characteristics on the effect of aerobic exercise training in patients with chronic heart failure: A meta-regression analysis. International journal of cardiology. 2016;208:120-7.
Waller B, Ogonowska-Slodownik A, Vitor M, Lambeck J, Daly D, Kujala UM, et al. Effect of therapeutic aquatic exercise on symptoms and function associated with lower limb osteoarthritis: systematic review with meta-analysis. Phys Ther. 2014;94(10):1383-95.
Wang Y-Q, Liu X, Yin Y-Y, Ma R-C, Yang Z, Cao H-P, et al. Effects of home-based exercise training for patients with lung cancer. Oncology nursing forum. 2019;46(4):E119-E34.
Way KL, Vidal-Almela S, Moholdt T, Currie KD, Aksetoy ILA, Boidin M, et al. Sex Differences in Cardiometabolic Health Indicators after HIIT in Patients with Coronary Artery Disease. Medicine and science in sports and exercise. 2021;53(7):1345-55.
Wayne PM, Lee MS, Novakowski J, Osypiuk K, Ligibel J, Carlson LE, et al. Tai Chi and Qigong for cancer-related symptoms and quality of life: a systematic review and meta-analysis. Journal of cancer survivorship : research and practice. 2018;12(2):256-67.
Wen-hua L, Jin-wu C, Xin C, Lin L, Hai-yan Y, Yu-qi Z, et al. Impact of Resistance Training in Subjects With COPD: A Systematic Review and Meta-Analysis. Respiratory Care. 2015;60(8):1130-45.
Wevers L, van de Port I, Vermue M, Mead G, Kwakkel G. Effects of task-oriented circuit class training on walking competency after stroke: a systematic review. Stroke. 2009;40(7):2450-9.
Wewege MA, Booth J, Parmenter BJ. Aerobic vs. resistance exercise for chronic non-specific low back pain: A systematic review and meta-analysis. Journal of Back & Musculoskeletal Rehabilitation. 2018;31(5):889-99.
Wibowo RA, Nurámalia R, Nurrahma HA, Oktariani E, Setiawan J, Icanervilia AV, et al. The Effect of Yoga on Health-Related Fitness among Patients with Type 2 Diabetes Mellitus: A Systematic Review and Meta-Analysis. Int J Environ Res Public Health. 2022;19(7).
Wieland LS, Skoetz N, Pilkington K, Vempati R, D'Adamo CR, Berman BM. Yoga treatment for chronic non-specific low back pain. Cochrane Database of Systematic Reviews. 2017;2017(1):CD010671.
Wiles L, Cafarella P, Williams MT. Exercise training combined with psychological interventions for people with chronic obstructive pulmonary disease. Respirology (Carlton, Vic). 2015;20(1):46-55.
Wilt TJ, Niewoehner D, MacDonald R, Kane RL, Wilt TJ, Niewoehner D, et al. Management of stable chronic obstructive pulmonary disease: a systematic review for a clinical practice guideline. Annals of internal medicine. 2007;147(9):639-I41.
Wind J, Koelemay MJ. Exercise therapy and the additional effect of supervision on exercise therapy in patients with intermittent claudication. Systematic review of randomised controlled trials. Eur J Vasc Endovasc Surg. 2007;34(1):1-9.
Wong WM, Chan DNS, He X, So WKW. Effectiveness of Pharmacological and Nonpharmacological Interventions for Managing the Fatigue-Sleep Disturbance-Depression Symptom Cluster in Breast Cancer Patients Undergoing Chemotherapy: A Systematic Review. Cancer nursing. 2022.
Wu C, Xu Y, Guo H, Tang C, Chen D, Zhu M. Effects of Aerobic Exercise and Mind-Body Exercise in Parkinson's Disease: A Mixed-Treatment Comparison Analysis. Frontiers in Aging Neuroscience. 2021;13:739115.
Wu M, Tang Q, Wang L, Zhang M, Song W, Teng L, et al. Efficacy of Traditional Chinese Exercise in Improving Gait and Balance in Cases of Parkinson's Disease: A Systematic Review and Meta-analysis. Front Aging Neurosci. 2022;14:927315.
Wu P-L, Lee M, Huang T-T. Effectiveness of physical activity on patients with depression and Parkinson's disease: A systematic review. PloS one. 2017;12(7):e0181515.
Wu S, Chen J, Wang S, Jiang M, Wang X, Wen Y. Effect of Tai Chi Exercise on Balance Function of Stroke Patients: A Meta-Analysis. Med Sci Monit Basic Res. 2018;24:210-5.
Wu X, Gao S, Lian Y. Effects of continuous aerobic exercise on lung function and quality of life with asthma: a systematic review and meta-analysis. J Thorac Dis. 2020;12(9):4781-95.

Wu X, Yang L, Wang Y, Wang C, Hu R, Wu Y. Effects of combined aerobic and resistance exercise on renal function in adult patients with chronic kidney disease: a systematic review and meta-analysis. Clinical rehabilitation. 2020;34(7):851-65.
Xia T-L, Huang F-Y, Peng Y, Huang B-T, Pu X-B, Yang Y, et al. Efficacy of Different Types of Exercise-Based Cardiac Rehabilitation on Coronary Heart Disease: a Network Meta-analysis. Journal of general internal medicine. 2018;33(12):2201-9.
Xiao L, Duan H, Li P, Wu W, Shan C, Liu X. A systematic review and meta-analysis of Liuzijue in stable patients with chronic obstructive pulmonary disease. BMC Complementary Medicine & Therapies. 2020;20(1):N.PAG-N.PAG.
Xiong X, Wang P, Li S, Zhang Y, Li X. Effect of Baduanjin exercise for hypertension: a systematic review and meta-analysis of randomized controlled trials. Maturitas. 2015;80(4):370-8.
Xu S, Zhang D, He Q, Ma C, Ye S, Ge L, et al. Efficacy of Liuzijue Qigong in patients with chronic obstructive pulmonary disease: A systematic review and meta-analysis. Complementary therapies in medicine. 2022;65:N.PAG-N.PAG.
Xu X, Ji W, Lv XQ, Zhu YC, Zhao JX, Miao LZ. Impact of physical activity on health-related quality of life in osteoporotic and osteopenic postmenopausal women: A systematic review. International Journal of Nursing Sciences. 2015;2(2):204-17.
Yamamoto R, Ito T, Nagasawa Y, Matsui K, Egawa M, Nanami M, et al. Efficacy of aerobic exercise on the cardiometabolic and renal outcomes in patients with chronic kidney disease: a systematic review of randomized controlled trials. Journal of Nephrology. 2021;34(1):155-64.
Yamamoto S, Hotta K, Ota E, Mori R, Matsunaga A. Effects of resistance training on muscle strength, exercise capacity, and mobility in middle-aged and elderly patients with coronary artery disease: A meta-analysis. Journal of Cardiology. 2016;68(2):125-34.
Yan JH, Pan L, Zhang XM, Sun CX, Cui GH. Lack of efficacy of Tai Chi in improving quality of life in breast cancer survivors: a systematic review and meta-analysis. Asian Pac J Cancer Prev. 2014;15(8):3715-20.
Yang C, Moore A, Mpofu E, Dorstyn D, Li Q, Yin C. Effectiveness of combined cognitive and physical interventions to enhance functioning in older adults with mild cognitive impairment: A systematic review of randomized controlled trials. The Gerontologist. 2020;60(8):e633-e42.
Yang Y, Li XY, Gong L, Zhu YL, Hao YL. Tai Chi for improvement of motor function, balance and gait in Parkinson's disease: a systematic review and meta-analysis. PLoS One. 2014;9(7):e102942.
Yang YJ, He XH, Guo HY, Wang XQ, Zhu Y. Efficiency of muscle strength training on motor function in patients with coronary artery disease: A meta-analysis. International Journal of Clinical and Experimental Medicine. 2015;8(10):17536-50.
Yang YL, Wang YH, Wang SR, Shi PS, Wang C. The effect of Tai Chi on cardiorespiratory fitness for coronary disease rehabilitation: A systematic review and meta-analysis. Frontiers in Physiology. 2018;8(JAN):1091.
Yardley JE, Hay J, Abou-Setta AM, Marks SD, McGavock J. A systematic review and meta-analysis of exercise interventions in adults with type 1 diabetes. Diabetes Res Clin Pract. 2014;106(3):393-400.
Yeh S-W, Lin L-F, Chen H-C, Huang L-K, Hu C-J, Tam K-W, et al. High-intensity functional exercise in older adults with dementia: A systematic review and meta-analysis. Clinical rehabilitation. 2021;35(2):169-81.
Yelin D, Moschopoulos CD, Margalit I, Gkrania-Klotsas E, Landi F, Stahl JP, et al. ESCMID rapid guidelines for assessment and management of long COVID. Clinical microbiology and infection : the official publication of the European Society of Clinical Microbiology and Infectious Diseases. 2022.
Ying M, Zhao R, Jiang D, Gu S, Li M. Lifestyle interventions to alleviate side effects on prostate cancer patients receiving androgen deprivation therapy: a meta-analysis. Japanese journal of clinical oncology. 2018;48(9):827-34.
You Y, Liu J, Tang M, Wang D, Ma X. Effects of Tai Chi exercise on improving walking function and posture control in elderly patients with knee osteoarthritis: A systematic review and meta-analysis. Medicine (Baltimore). 2021;100(16):e25655.
Yu B, Tong S, Wu Y, Abdelrahim MEA, Cao M. Effects of resistance training on exercise ability in chronic obstructive pulmonary disease subjects: A systematic review and meta-analysis. Int J Clin Pract. 2021;75(9):e14373.
Yue Y-S, Wang X-D, Xie B, Li Z-H, Chen B-L, Wang X-Q, et al. Sling exercise for chronic low back pain: a systematic review and meta-analysis. PloS one. 2014;9(6):e99307.
Zeng Y, Huang M, Cheng AS, Zhou Y, So WK. Meta-analysis of the effects of exercise intervention on quality of life in breast cancer survivors. Breast Cancer. 2014;21(3):262-74.
Zeng Y, Xie X, Cheng ASK. Qigong or Tai Chi in Cancer Care: an Updated Systematic Review and Meta-analysis. Curr Oncol Rep. 2019;21(6):48.

Zhang H, Chang R. Effects of Exercise after Percutaneous Coronary Intervention on Cardiac Function and Cardiovascular Adverse Events in Patients with Coronary Heart Disease: Systematic Review and Meta-Analysis. Journal of sports science & medicine. 2019;18(2):213-22.

Zhang J, Yang KH, Tian JH, Wang CM. Effects of yoga on psychologic function and quality of life in women with breast cancer: a meta-analysis of randomized controlled trials. J Altern Complement Med. 2012;18(11):994-1002.

Zhang Q, Pang R, Cai Z. Effect and safety of traditional Chinese exercises (Qigong therapy) for patients with chronic obstructive pulmonary disease: A protocol for systematic review and meta-analysis. Medicine (Baltimore). 2022;101(49):e32284.

Zhang WW, Speare S, Churilov L, Thuy M, Donnan G, Bernhardt J. Stroke rehabilitation in China: a systematic review and meta-analysis. Int J Stroke. 2014;9(4):494-502.

Zhao Q-G, Zhang H-R, Wen X, Wang Y, Chen X-M, Chen N, et al. Exercise interventions on patients with end-stage renal disease: a systematic review. Clinical rehabilitation. 2019;33(2):147-56.

Zhao Y, Feng H, Wu X, Du Y, Yang X, Hu M, et al. Effectiveness of Exergaming in Improving Cognitive and Physical Function in People With Mild Cognitive Impairment or Dementia: Systematic Review. JMIR Serious Games. 2020;8(2):e16841.

Zhou L, Chen Q, Zhang J. Effect of Exercise on Fatigue in Patients with Lung Cancer: A Systematic Review and Meta-Analysis of Randomized Trials. Journal of palliative medicine. 2021;24(6):932-43.

Zhou Y, Zhu J, Gu Z, Yin X. Efficacy of exercise interventions in patients with acute leukemia: A meta-analysis. PloS one. 2016;11(7):e0159966.

Zhu G, Zhang X, Wang Y, Xiong H, Zhao Y, Sun F. Effects of exercise intervention in breast cancer survivors: A meta-analysis of 33 randomized controlled trails. OncoTargets and Therapy. 2016;9:2153-68.

Zhu H, Jin J, Zhao G. Effect of aquatic exercise on cardiovascular fitness in people with type 2 diabetes mellitus: a systematic review and meta-analysis. Kardiologiia. 2021;61(9):52-60.

Zhu L, Li L, Wang L, Jin X, Zhang H. Physical Activity for Executive Function and Activities of Daily Living in AD Patients: A Systematic Review and Meta-Analysis. Front Psychol. 2020;11:560461.

Zhu L, Wu W, Chen M, Xu D, Xu H, Liu L, et al. Effects of Nonpharmacological Interventions on Balance Function in Patients with Osteoporosis or Osteopenia: A Network Meta-Analysis of Randomized Controlled Trials. Evidence-based Complementary and Alternative Medicine. 2021;2021:6662510.

Zhu XY, Li Z, Chen C, Feng RL, Cheng BR, Liu RY, et al. Physical Therapies for Psychosomatic Symptoms and Quality of Life Induced by Aromatase Inhibitors in Breast Cancer Patients: A Systematic Review and Meta-Analysis. Frontiers in Oncology. 2021;11:745280.

Zou L, Yeung A, Zeng N, Wang C, Sun L, Thomas GA, et al. Effects of mind-body exercises for mood and functional capabilities in patients with stroke: An analytical review of randomized controlled trials. International journal of environmental research and public health. 2018;15(4):721.

Zou L, Zhang Y, Yang L, Loprinzi PD, Yeung AS, Kong J, et al. Are mindful exercises safe and beneficial for treating chronic lower back pain? A systematic review and meta-analysis of randomized controlled trials. Journal of Clinical Medicine. 2019;8(5):628.

Zwisler AD, Norton RJ, Dean SG, Dalal H, Tang LH, Wingham J, et al. Home-based cardiac rehabilitation for people with heart failure: A systematic review and meta-analysis. Int J Cardiol. 2016;221:963-9.

4. Primary study overlap

Total primary studies included across all selected systematic reviews and supplementary RCT studies = 1,218. Of these, 990 were RCTs that met our inclusion criteria. The primary studies listed in the table below overlapped across the listed systematic reviews (non-overlapping primary studies and systematic reviews not shown). Blue studies met our inclusion criteria and were considered in our results, orange studies were not eligible (non-RCT) and did not contribute to our results/conclusions. The total corrected covered area (CCA) was calculated as 0.02%.

**LTC** / **Primary Study**	**Cancer** (haem; Knips 2019)<br><br>18 studies included	**Anaemia** (Courneya 2009)<br><br>Single RCT	**Cancer** (advanced metastatic; Chen 2020)<br><br>15 studies included	**Alcohol Problems** (Gur 2020)<br><br>10 studies included	**Psychoactive substance abuse** (Dowla 2022)<br><br>42 studies included	**Arthritis** (osteo-hip; Fransen 2014)<br><br>10 studies included	**Arthritis** (osteo-knee; Fransen 2015)<br><br>54 studies included
Courneya 2009	x	x					
Chang 2008	x		x				
Gur 2017				x	x		
Giesen 2016				x	x		
Hallgreen 2014				x	x		
Abbott 2013						x	x
Van Baar 1998						x	x

5. Supplementary Table 2 – Characteristics of LTC participants across selected SRs and RCTs as reported by authors

LTC	Population definition	Comorbidities considered	Age (mean age range unless otherwise stated)	Sex (median % female, unless otherwise stated)	Diversity information (eg. SES, ethnicity)
**Alcohol problems**	All participants had a diagnosis of AUD based on Diagnostic and Statistical Manual of Mental Disorders (DSM)-I, DSMII, DSM-III, DSM-III-R, DSM-IV, DSM-V, the International Classification of Disease, Tenth Revision (ICD-10), the Alcohol Use Disorders Identification Test (AUDIT), or guidelines of the National Institute on Alcohol Abuse and Alcoholism. We excluded studies with a heterogeneous sample consisting of AUD participants (eg, SUBs and post-traumatic disorders).”	No	20.1 to 48.2 years	33.3%	Not reported
**Anaemia***	Eligible patients had a histologically confirmed nonmyleoid cancer diagnosis, an Hb level of 80-110 g/l, an Eastern Cooperative Oncology Group performance status score of 0-2, completed definitive surgery, an expected survival duration ≥3 months, and were English speaking and ≥18 years of age.	Participants had cancer diagnoses alongside mild-to-moderate anaemia.<br><br>Exclusion criteria included: iron deficiency (ferritin <12μg/l), had received an erythropoiesis-stimulating agent (ESA) within 4 weeks of randomization, or had uncontrolled hypertension, cardiac abnormalities, a psychiatric illness, a known hematologic disorder causing anaemia, substantial lung, pleural, or pericardial disease, pre-existing bone metastases at high risk for fracture, or contraindications to maximal exercise testing.	Mean age 56 years	81.8%	Completed university: 43.6%<br><br>Income >$60,000/year: 25.5%
**Anorexia**	Patients with anorexia nervosa	Exclusion criteria: “other diagnoses”, not specified further	20 to 29 years	99% female	“predominantly Caucasian females”
**Anxiety**	Participants had elevated anxiety symptoms using a validated assessment instrument or were diagnosed with an anxiety disorder	Some of the studies allowed individuals to participate if they had either elevated anxiety or another condition (e.g., depression, medical comorbidities). Studies that did not provide data on anxiety related outcomes specifically for the participants with elevated pre-treatment anxiety were excluded	19 to 64 years	range across included studies: 11-100%	Not reported
**Arthritis (osteo-, hip)**	Adults, men or women, with either an established diagnosis of hip OA according to accepted criteria or self reporting hip OA on the basis of chronic anterior joint pain (without radiographic confirmation).	No	58 to 70 years	range 75-80% female (only reported in 3 studies)	Not reported
**Arthritis (osteo-, knee)**	Male and female adults given an established diagnosis of knee OA according to accepted criteria (Altman 1991), or	“Many study cohorts comprised participants who were overweight	53 to 75 years	range 42-100% female	Not reported

	who self reported knee OA on the basis of chronic joint pain (with or without radiographic confirmation).	(body mass index (BMI) 25 to 29.9 kg/m2) or obese (BMI ≥ 30 kg/m2).”			
**Arthritis (rheumatoid)**	Patients with RA and did not have other serious diseases	Inclusion criteria – without other serious diseases	41.4 to 66.6 years	range 62 to 83% female	Not reported
**Asthma**	Human adults (≥18 years) with a clinical diagnosis of asthma as the primary respiratory disease	No	22 to 68 years	Range 36 to 98%	Not reported
**Atrial fibrillation**	Adults with atrial fibrillation (aged 18 and over with no upper limit). Specified populations (permanent AF, persistent AF, paroxysmal AF, chronic AF)	No	Not reported	65%	Not reported
**Bronchiectasis**	Individuals diagnosed with non-CF bronchiectasis according to physician diagnosis or clinical evidence or high resolution computed tomography	No	Not reported	Range 19-52%	Not reported
**Cancer (solid tumour)**	Included adult patients (aged ≥18), patients diagnosed with cancer, patients who had completed their main treatment for cancer but might be still undergoing hormonal treatment	No	Mean 55 (range 39-74) years	range 0-100%	Not reported
**Cancer (haematological)**	Adults (18 years and over) with confirmed diagnoses of haematological malignancies	No	20.8 to 58.8 years	Not reported	Not reported
**Cancer (advanced metastatic)**	Cancer that has spread to other places in the body and usually cannot be cured or controlled with treatment	No	47.5 to 72.4 years	27 to 67%	Not reported
**Chronic fatigue syndrome**	Male and female participants over the age of 18 years, irrespective of cultures and settings. Included studies in which participants fulfilled the following diagnostic criteria for CFS or ME:<br>• Fatigue, or a symptom synonymous with fatigue, was a prominent symptom;<br>• Fatigue was medically unexplained (i.e. other diagnoses known to cause fatigue such as anorexia nervosa or sleep apnoea could be excluded);<br>• Fatigue was sufficiently severe to significantly disable or distress the participants;<br>• Fatigue persisted for at least 6 months.	Inclusion criteria: studies involving participants with co-morbid physical or common mental disorders were eligible for inclusion only if the co-morbidity did not provide an alternative explanation for fatigue.<br><br>Depression ranged from 18-39% participants	33 to 44.6 years	range 65-78%	34-39% working or studying at least part time (where reported)
**Chronic kidney disease**	Chronic kidney disease, single or both genders of age ≤80 years.<br>Included haemodialysis and non dialysis dependent CKD patients (stages not specified)	No	18 to 75 years	Not reported	Not reported
**Chronic liver disease**	Participants with cirrhosis of any age or sex, irrespective of the severity or aetiology.<br>Included %range:<br>Alcohol 7.7-86%<br>Chronic hepatitis C 5-38.5%<br>Non-alcoholic steatohepatitis 0-33.3%	No	51 to 65.5 years	16.7 to 50%	Not reported
**Chronic obstructive pulmonary disease**	Clinical diagnosis of COPD according to the Global Obstructive Lung Disease Initiative criteria (Global Strategy for the Diagnosis, Management and Prevention of COPD)	No	52 to 77.6 years	0-70%	Not reported
**Connective tissue disease**	Interstitial lung disease of any origin, diagnosed according to investigator definitions	No	36 to 72 years	28-67%	Not reported

**Coronary heart disease**	Adult (≥18 years) men and women, in either hospital-based or community-based settings, who have had a myocardial infarction (MI), or who have undergone revascularisation (CABG, PCI) or who have angina pectoris or coronary artery disease defined by angiography.	No	47 to 77 years	<15% overall	Not reported
**Dementia**	Primary diagnosis of mild cognitive impairment or dementia	No	65.3 to 89.2 years	Not reported	Not reported
**Depression**	Adults with a primary diagnosis of major depressive disorder (MDD) according to established criteria (e.g., Diagnostic and Statistical Manual of Mental Disorders (DSM)(American PsychiatricAssociation,1994, 2013) or International Classification of Diseases (ICD) (World Health Organization, 1993) or those with increased depressive symptoms determined by a validated screening measure (e.g., Hamilton Rating Scale for Depression, (HAM-D), (Hamilton,1967), Beck Depression Inventory (BDI)(Beck etal.,1961), Geriatric Depression Scale (GDS) (Yesavage,1988) or other) according to the author's criteria. Also included studies meeting criteria that included some participants with other related diagnoses, such as dysthymia. This decision was based on the fact that dysthymia is categorized as a chronic and milder disorder within the depressive disorder spectrum (American PsychiatricAssociation, 2013)"	One study included participants with depression and participants with additional co-morbid diagnoses, such as cardiovascular diseases	38.8 to 76.4 years	50-100%	Not reported
**Diabetes mellitus**	Males and females with type 2 diabetes. Ideally, the diagnostic criteria for type 2 diabetes mellitus should have been described in the trial. To be consistent with changes in classification and diagnostic criteria of diabetes through the years, the diagnosis should have been established using the standard criteria valid at the beginning of the trial. Acceptable diagnostic criteria included those described by: the National Diabetes Data Group standards (NDDG 1979), the World Health Organisation standards (WHO 1980; WHO 1999) or the American Diabetes Association standards (ADA 1997b).	No	45 to 65 years	0-70%	Not reported
**Endometriosis**	Women with any degree of endometriosis as diagnosed with an imaging or surgical modality, who presented with pain in the pelvic region (including dysmenorrhea, dyspareunia, or CPP).	No	Not reported	100%	Not reported
**Epilepsy**	Eligible participants were people with all types of epilepsy, of all age groups, and both genders.	No	19.7 to 25.8 years	72%	Not reported
**Glaucoma**	Primary open angle glaucoma	No	Not reported	Not reported	Not reported
**Heart failure**	Adults aged 18 years of older with HF. Participants were predominantly HFrEF and NYHA classes II and III.	2 studies reported rates of comorbidity	51 to 81 years	Median 21%	Only 10 trials reported on ethnicity – 47-100% white
**Hypertension**	Hypertensive, SBP>130 mmHg or DBP >80 mmHg, following the American College of Cardiology/American Heart Association	No	52.2 to 76 years	0-100%	Not reported

**Inflammatory bowel disease**	**Adults ≥18 years diagnosed with either Chron's disease or ulcerative colitis.**	**No**	**Not reported**	**Not reported**	**Not reported**
**Irritable bowel syndrome**	Inclusion criteria Not reported<br>Diagnostic criteria of included participants:<br>Rome II (n=3 studies)<br>Rome III (n=8 studies)	No	19.6 to 53.6	Not reported	Not reported
**Long-COVID**	Adults (>18 years) diagnosed with COVID-19 at least four weeks before study enrollment, according to the definition of PACS	Exclusion criteria in eligible study: uncontrolled hypertension, uncontrolled chronic disease, cerebrovascular disease within 6 months, Intra-articular drug injection or surgical treatment of lower extremities within 6 months, Inability to walk independently with assistive device, History of severe cognitive or mental disorder or substance abuse"	49.2 to 69.4 (over all studies including non-eligible)	Mean 45% (over all studies including non-eligible)	Not reported
**Migraine**	Patients older than 18 years old and diagnosed with primary headaches based on the International Classification of Headache Disorders (ICHD), and referred by a physician were included	No	25.1 to 48 years	85%	Not reported
**Multiple sclerosis**	Adults aged>18 years with a definite diagnosis of MS, regardless of sex, disease duration, MS phenotype, or level of disability were considered eligible for inclusion	No	31.3 to 56 years	range 0-100%	Not reported
**Osteoporosis**	Persons (women and men) as community dwellers (not hospitalized) over the age of 45 diagnosed with osteoporosis (defined as a T-score≤−2.5 SD) with or without a fracture and with osteopenia (defined as a T-score between−1.0 and−2.5 SD) below the norm mean for a healthy female young adult.	Only mentioned that there was considerable diversity in the presence of comorbidities and medication use across studies	56 to 82 years	94.9% (based on all included trials – some ineligible)	"There was considerable diversity in terms of the socio demographic factors of participants." No further information provided
**Painful condition (chronic back pain)**	Adult participants with chronic nonspecific low back pain of more than 12 weeks' duration (defined as meaning back pain duration of the study group was greater than or equal to 12 weeks)	No	43.7 (95% CI 35.8 to 44.9)	59.7% (95% CI 55.1 to 64.2%)	Not reported
**Painful condition (fibromyalgia)**	Adults with fibromyalgia in studies that used published criteria for diagnosis (or classification) of fibromyalgia. Diagnosis could be based on ACR 1990 criteria - the preliminary diagnostic tool (Wolfe 1990), ACR 2010 criteria (Wolfe 2010), or a follow-up survey questionnaire (Wolfe 2011).	No	43.2 to 59 years	98%	Not reported
**Parkinson's disease**	Studies had to investigate people with PD according to established criteria, include people with PD scoring 1–3 on the Hoehn and Yahr scale	No	mean 65 years (range 59-76 years)	M:F ratio 2.1 : 1	Not reported
**Peripheral vascular disease**	Symptomatic intermittent claudication due to atherosclerotic disease. Intermittent claudication may be diagnosed objectively by an ABI < 0.9 or evidence of PAD on Doppler ultrasound or angiography, or both, or by questionnaire or	No	44 to 71 years	range 0-100%	Not reported

	clinically if objective measures such as ABI or imaging were not used or reported				
**Polycystic ovarian syndrome**	Reproductive-aged women with a diagnosis of polycystic ovary syndrome (PCOS) based on the National Institute of Health (NIH) diagnostic criteria (1990), the Rotterdam ESHRE/ASRM (2003) diagnostic criteria or the AE-PCOS Criteria (2006). We also included trials where the PCOS diagnosis had been verified by a general practitioner or specialist clinician.	Participants with T2DM, fasting hyperglycaemia, or glucose intolerance were explicitly excluded in 9 trials (50%), and 9 trials also excluded participants with any diagnosed CVD.	21.8 to 35.2 years	100%	Not reported
**Prostate disorders**	Diagnosed with prostate cancer	No	Not reported	0%	Not reported
**Psychoactive substance misuse**	Participants seeking treatment for substance use disorder (including patients who were engaged in a drug or alcohol treatment or had received a Diagnostic and Statistical Manual of mental disorders, fifth edition diagnosis for alcohol or any illicit or prescription drug)<br>Included alcohol use disorder (n=7), polysubstance use (6), stimulant type drugs (6), opioid (3), heroin (3)	4 studies excluded participants with additional substance use disorders (additional to alcohol use disorder)	27 to 55 years	22%	Not reported
**Schizophrenia**	Patients with schizophrenia-spectrum disorder (SSD) or first episode of psychosis (FEP). SSD encompassed ICD-10 codes F20-F29: schizophrenia (SCZ), schizotypal, delusional, and other non-mood psychotic disorders. Schizophrenia only (n=25), any SSD diagnosis (n=3), FEP only (n=3), mixed FEP + SSD (n=7), other conditions included Schizoaffective disorder (SAD), Delusional disorder (DD), Acute and transient psychosis (ATP), Schizophreniform (SCZPH)	No	18 to 63 years	range 0-100%	Not reported
**Stroke or TIA**	Adult stroke survivors who were considered suitable for fitness training by the studies' authors; we used the study authors' definition of stroke. Participants were considered eligible irrespective of the time since stroke onset.	People with comorbidities excluded in 14 studies	Approximately 62 years	Range 13-85%	1 study included people with below ‘per capita’ income, below minimum wage was an inclusion criteria
**Treated constipation**	Study participants were adults (≥18 years), diagnosed with non-drug or other disease-induced constipation; the participants were able to exercise without assistance	Exclusion criteria: patients with mental illnesses or cognitive disorders. 6 studies excluded due to constipation caused by disease	33.25 to 86.15 years	Not reported	Not reported
**Viral hepatitis***	Study 1: Cirrhotic adults aged 18–65 with a Child-Turcotte-Pugh (CTP) score ≤ 6 were eligible for inclusion. Cirrhosis was diagnosed using computed tomography or magnetic resonance imaging, transient elastography with liver stiffness measurement ≥ 13 kPa, or liver biopsy (Metavir fibrosis score = 4). The etiologies of cirrhosis included chronic hepatitis B (CHB) on antiviral drugs with virological suppression, chronic hepatitis C (CHC) with the sustained virological response (SVR), alcohol with abstinence ≥ 1 year, or non-alcoholic steatohepatitis (NASH). Patients were excluded if they had one or more of	Study 1: Exclusion criteria: significant comorbid diseases, such as cardiac diseases (coronary artery disease, cardiac arrhythmia, impaired ejection fraction < 60%, or positive exercise stress test ≥ 1 mm ST depression), chronic renal failure on dialysis, hematologic diseases (hemoglobin < 11 g/dL or platelet count < 50,000 cell/mm3), myopathy, or chronic lung	55.5 to 56.3 years	range 14-35%	Not reported

	the following conditions: (1) body mass index (BMI) ≥ 28 kg/m2; (2) large esophageal varices (EV) or uncontrolled EV or gastric varices; (3) active malignancy, including hepatocellular carcinoma; or, (4) significant comorbid diseases, such as cardiac diseases (coronary artery disease, cardiac arrhythmia, impaired ejection fraction < 60%, or positive exercise stress test ≥ 1 mm ST depression), chronic renal failure on dialysis, hematologic diseases (hemoglobin < 11 g/dL or platelet count < 50,000 cell/mm3), myopathy, or chronic lung diseases (severe asthma and chronic obstructive pulmonary disease).<br><br>Study 2: All registered adults in the Midwest area of the Health Services Executive, Ireland, who had contracted hepatitis C through contaminated blood products were eligible. Candidates younger than 18 years and those infected through alternative means were excluded.	diseases (severe asthma and chronic obstructive pulmonary disease).<br><br>Study 2: Total number of comorbidities (median, range) 2 (0-5). Comorbidities not described. "A simple count of medical diagnoses was used to quantify the level of comorbid disease in the cohort.	

*RCT evidence only identified. CI: confidence interval; NR: not reported; SES: socioeconomic status.

6. Supplementary Table 3 – Characteristics of exercise interventions of selected SRs and RCTs as reported by authors

Condition	N Exercise only / multi-component[a]	Exercise frequency (sessions/ week)	Exercise intensity	Duration (per session)	Exercise type/mode	Resistance/ strength training included	Overall duration (range)	Setting/ delivery method	Modi-fications for co-morbidities?	Intervention reporting quality assessment (Tidier/CERT – mean score)
**Alcohol problems**	0/5	1 to 3	55-90% HRmax or <40-<60% VO2 peak	20-90 mins	Aerobic	No	8 to 24 weeks	Centre-based (hospital/ clinic)	Not reported	Not done
**Anaemia (RCT only)**	1	3	60-100% baseline peak power output	Not reported	Aerobic, cycle ergometry	No	12 weeks	Not reported	Individually tailored to each participant	CERT – total score = 8[c]
**Anorexia**	2/1	2 to 4	Not reported	60 mins	Flexibility, anaerobic and resistance/ strength, low/high impact aerobic, yoga, recreational games	Yes – included in 3 studies	2.5 weeks to 3 months	Eating disorder outpatient clinic, eating disorder unit, residential eating disorder	Not reported	Not done
**Anxiety**	8/4	1 to 5	50-90% HRmax or work output	16-90 mins	Aerobic (n=8), aerobic + resistance (n=2), mindfulness-based stretching (n=1), participants choice (n=1)	Yes – included in 2 studies	2 weeks – 6 months	supervised exercise (n=8), partially supervised (n=2), unsupervised (n=2)		Not done
**Arthritis (osteo-, hip)**	8/2	1 to 3	Not reported	30-60 mins	Aerobic, resistance, flexibility, tai chi, range of movement	Yes – included in 6 studies	6-12 weeks	2 described as provided by physiotherapist	Not reported	Not done
**Arthritis (osteo-, knee)**	41/16[b]	1 to 5	moderate, 60-80% HRmax, 50-80% 1RM, 50-85% HRreserve	20-60 mins	Strength, combination (strength + aerobic), aerobic, tai chi, baduanjin	Yes – included in 37 studies	1 to 24 months	Concurrent clinic/home (n=4); home based (n=7); classes (n=25); individual (n=18)	Not reported	Not done
**Arthritis (rheumatoid)**	13/0	1 to 7	30-100% max load, aerobic Not reported	5-80 min	Aerobic (water based, walking), combination, tai chi, resistance	Yes – included in 8 studies	3-24 weeks	Not reported	Not reported	Not done
**Asthma**	12/11[b]	1 to 5	60-90% HRmax, 60-90% VO2 peak, RPE 7-8(/10) or 12-14(/20), 60-100% max power output	20-90 min	Aerobic (walking, circuit training, bodyweight HIIT), resistance, yoga	Yes – included in 3 studies	4 weeks -12 months	Community, outpatient clinic, lab. Supervised by exercise physiologists, physicians, respiratory therapist.	Not reported	Not done
**Atrial fibrillation**	12/0	1 to 3	30-95% HRmax or 40-70% max	30-90 min	Aerobic, anaerobic, combination (aerobic	Yes – included in 4 studies	8 weeks – 6 months	Not reported	Not reported	Not done

			exercise capacity		+ anaerobic), qigong, yoga, resistance					
**Bronchiectasis**	0/4	2 to 7	75-85% VO2 max, 80% HR peak, RPE 12-14, 60-80% 1RM	20-45 min	Aerobic (treadmill walking, cycling, stair climbing, ski machine), resistance training	Yes – included in 3	8 weeks	Hospital outpatient or inpatient	Not reported	Not done
**Cancer (solid tumour)**	34/0	1 to 7	25-85% HRmax, 60-75% VO2 peak, moderate-vigorous, RPE 10-16	10-90 min	Aerobic (walking, cycling), strength/ resistance training	Yes – included in 4 studies	Median 13 weeks, range 3-60 weeks	Not reported	Not reported	Not done
**Cancer (haematological)**	15/3	2 to 5	Low to high, 50-70% HR reserve, 60-75% peak power output	10-70 min	Aerobic (walking, cycling), resistance	Yes – included in 4 studies	5-15 weeks	Not reported	Not reported	Not done
**Cancer (advanced metastatic)**	15/0	2 to 8	55-95% HRmax, >3 MET hours/ week, 60-80% VO2 peak, 30-80% max work rate, 40-70% 1RM, ≥13 RPE	20-120 min	Endurance, strength, walking, Nordic walking, yoga	Yes – included in 9 studies	0.5-6 months	Supervised by certified physiotherapist or instructor in 14 studies	Not reported	Not done
**Chronic fatigue syndrome**	7/2[b]	3 times per week to several times per day	40-75% VO2max	5-90 min	Aerobic (graded exercise training)	No	12-26 weeks	"most of the included studies encouraged participants to exercise at home"	Not reported	Not done
**Chronic kidney disease**	11/0	3 to 4	50-70% HR max, 40% 1RM; 11-15 RPE, mild to moderate	Not reported	Aerobic, resistance, combination (aerobic and resistance), inspiratory muscle training	Yes – included in 6 studies	8-24 weeks	9 intradialytic, 2 non-dialysis. Home based mentioned in 1 study	Not reported	Not done
**Chronic liver disease**	3/3	2 to 3	moderate to high, 60-80% VO2 peak	30-70 min	Aerobic, combination, resistance	Yes – included in 3 studies	8-14 weeks	One home based, otherwise Not reported. Delivered by exercise specialist, physiotherapist, nurses, cardiologist	Not reported	Not done
**Chronic obstructive pulmonary disease**	0/39	Not reported	Not reported	Not reported	yoga, tai chi, conventional exercises	Not reported	Not reported	Not reported	Not reported	Not done

**Connective tissue disease**	9/12	2 to 7	moderate to vigorous	30-120 min	endurance, pulmonary rehabilitation	Yes – included in 13 studies	5-48 weeks	Outpatient (n=18), inpatient (n=1), home-based (n=1), tele-rehab (n=1)	Not reported	Not done
**Coronary heart disease**	38/47	1 to 7	50-90% max/peak HR or HRR, 50-95% VO2 max, 11-16 RPE	20-90 min	most often aerobic including static cycling, walking or circuit training	Yes – included in 22 studies	Median 6 months, range 3 weeks to 42 months	21 home based programmes	Not reported	Not done
**Dementia**	30/8	1 to 7	30-70% VO2 max, "gentle", moderate to high, 40-85% HR reserve, 70-80% 1RM, 60% HRmax	15-150 min	aerobic, walking, dual-task, multimodal, ADL/functional training, strengthening, others	Yes – included in 3 studies	single session to ≥12 months	Institution (n=21), hospital (n=1), community (n=13), outpatient (n=2). Delivered by nurses, physiotherapist, researcher, physician, exercise scientist, occupational therapist, students, social centre staff	Not reported	Not done
**Depression**	0/6	2 to 5	light to moderate	Not reported	aerobic, mixed (yoga, tai chi, qigong)	No	3-32 weeks	outpatient (n=5), inpatient (n=1). Most studies supervised by exercise professionals	Not reported	Not done
**Diabetes mellitus**	11/3	1 to 7	65-75% HR reserve, 50-85% VO2max/ peak, up to 85% HR peak, low-moderate	30-120 min	aerobic, resistance, combination training, qigong	Yes – included in 7 studies	8 weeks-12 months	Not reported	Not reported	Not done
**Endometriosis**	0/2	2 to 4	50-70% HR max	40-120 min	cardio-fitness, flexibility, yoga	No	8-24 weeks	Not reported	Not reported	CERT – total score 12
**Epilepsy**	1/1	2 per day	Not reported	20-30 min	Yoga	No	5 weeks-6months	Trained instructor	Not reported	Not done
**Glaucoma**	0/1	7	Not reported	30 min	Not reported	No	Not reported	Not reported	Not reported	Not done
**Heart failure**	30/14	1 to 7	40-80% HRmax, 50-85% VO2 max, RPE 11-18	10-120 min	Aerobic, or aerobic + resistance	Yes – included in 14 studies	8-120 weeks	Centre-based, combination, or home-based (n=10)	Not reported	Not done
**Hypertension**	23/0	2 to 7	60-90% HRreserve, 45-75% VO2max, 50-80% 1RM, 11-13 RPE	20-210 min	Aerobic (walking, cycling, circuits), resistance (band, full body, upper/lower limbs & trunk)	Yes – included in 14 studies	6-36 weeks	Not reported	Not reported	Not done

**Inflammatory bowel disease**	5/2	1 to 7	60% VO2 max, 60% HR max	20 min – 6 hours	Aerobic, resistance, mind-body therapy (including yoga), lifestyle intervention	Yes – included in 3 studies	8 weeks- 1 year	1 reported as home based	Not reported	Not done
**Irritable bowel syndrome**	11/1[b]	2 per week to 2 per day	low to moderate	20-120 min	yoga, physical activity, Tai Ji, mountaineering	No	6-24 weeks	Not reported	Not reported	Not done
**Long-COVID**	0/2	1 to 4	40-80% HRmax	5-90 min	Aerobic + strength, pulmonary rehabilitation	Yes – included in 1 studies	6 weeks	Home-based	Not reported	Not done
**Migraine**	12/7	2 per week to every 2-3 hours	45-85% VO2 max, 70-95% HRmax, 13-16 RPE, 70-80% 12RM, ventilatory threshold	10-60 min	Aerobic, strength, combination, yoga	Yes – included in 8 studies	3 weeks – 6 months	Not reported	Not reported	TIDieR – total percentage 50%
**Multiple sclerosis**	22/0	1 to 5	60% VO2 max, RPE 8, 40-80% peak power output, 40-95% HRmax, 60-75% Wmax, 50-80% 1RM	27-69 min	Aerobic, resistance	Yes – included in 8 studies	3-26 weeks	Not reported	Not reported	Not done
**Osteoporosis**	15/1	1 to 7	50-80% 1RM	30-90 min	Gait, balance, functional, strength/ resistance, tai chi, multicomponent	Yes – included in 8 studies	5-52 weeks	Not reported	Not reported	Not done
**Painful condition (chronic back pain)**	<50%/>50 %	Not reported	“low dose”	Not reported	Resistance training, mixed, pilates, stretching, aerobic	Yes – included in 85 studies	0.14-26 weeks	Not reported	Not reported	Not done
**Painful condition (fibromyalgia)**	17/6	1 to 7	40-85% HRmax	45-180 min	Aerobic (walking, jogging, stationary bike), resistance (isotonic/ isometric), flexibility, belly dance, agility, co-ordination, balance	Yes – included in 19 studies	4-26 weeks	2 primarily home-based	Not reported	Not done
**Parkinson’s disease**	33/0	mean 2.7	60-85% HRmax, 30-90% 1RM	16-45 min	Treadmill, cycle, resistance – whole body programmes	Yes – included in 18 studies	4-104 weeks	Some home-based	Not reported	Not done
**Peripheral vascular disease**	30/2	2 to 7	Not reported	26-60 min	aerobic (walking, treadmill, arm cranking), resistance and combined training	Yes – included in 3 studies	2 weeks – 12 months	Primarily supervised and some included home-based exercise	Not reported	Not done
**Polycystic ovarian syndrome**	14/6[b]	3 to 5	40-95% HR max, 40-70% VO2 max/peak,	25-60 min	aerobic, resistance or combined	Yes – included in 6 studies	8-48 weeks	Not reported	Not reported	Not done

			1050-4200 kJ/week, 40-75% 1RM, 5-6/10 RPE							
**Prostate disorders**	16/0	2 to 7	55-100% HR max, 9-13 RPE, 3.0-6.0 METs, 55-100% VO2 peak, 500-1000 MET min/week	20-45 min	aerobic (walking, running), resistance or combined	Yes – included in 7 studies	8-36 weeks	Supervised (n=5), home-based (n=3), mix of supervised and home-based (n=4), supervised with suggested home-based (n=2)	Not reported	Not done
**Psycho-active substance misuse**	25/0	1 per week to 2 per day	Not reported	10-150 min	Aerobic, mind-body exercise (yoga/tai chi/qigong), aerobic + strength, strength only	Yes – included in 7	single session – 1 year	Inpatient addiction treatment facility/ rehabilitation centre/ residential treatment programme (n=15), outpatient unit/ treatment centre/clinic/AA/ community (n=5), withdrawal management (n=1)	Not reported	Not done
**Schizophrenia**	31/7	1 to 7	light to vigorous	20-360 min	aerobic, anaerobic, mind-body exercise (e.g. yoga), mixed (combination of aerobic + anaerobic or aerobic + mind-body)	Yes – included in 7	3-52 weeks	Inpatient (n=12), outpatient (n=26)	Not reported	Not done
**Stroke or TIA**	75/0	2 to 7	40-85% HR max, 40-70% HR reserve, 60-75% VO2 peak, 30-50% maximum effort, RPE ≤13, 40-100% 1RM	7-120 min	aerobic, resistance, mixed/combination	Yes – included in 43	2-24 weeks	Rehab centre/outpatient (n=34), community (n=10), hospital (n=12), home (n=5), mixed setting (n=3), exercise lab (n=1) Delivered by therapist, certified trainer, or PI assisted by physio students	Not reported	Not done
**Treated constipation**	9/0	2 to 7	Not reported	20-60 min	aerobic, resistance	Yes – included in 1 study	30 days – 24 weeks	Not reported	Not reported	Not done

**Viral hepatitis (RCT only)**	1/1	2 to 4	60-85% HR max	40-60 min	aerobic, resistance	Yes – included in 1 study	6-12 weeks	Centre based (n=1), home based (n=1). Delivered by sport scientist or physiotherapist	Not reported	CERT – total score 11

[a]Multicomponent = exercise + other (e.g., diet, education, psychological, medication); [b]: some studies had multiple intervention arms; [C]based on our own assessment
HR: heart rate; 1RM: one repetition max; VO2: oxygen consumption; RPE: rating of perceived exertion; TIDieR: Template for Intervention Description and Replication; CERT: Consensus on Exercise Reporting Template

7. Supplementary Table 4 – AMSTAR assessments for selected SRs

Condition	Q1	Q2*	Q3	Q4*	Q5	Q6	Q7*	Q8	Q9*	Q10	Q11*	Q12	Q13*	Q14	Q15*	Q16	Overall rating
Alcohol problems	Y	PY	N	N	N	Y	PY	PY	Y/N	N	N	N	N	Y	Y	Y	Low
Anorexia	Y	PY	N	N	Y	N	PY	PY	Y/N	N	Y	N	Y	Y	N	Y	Critically low
Anxiety	Y	N	N	N	Y	Y	N	PY	Y	N	NA	NA	Y	N	NA	Y	Low
Arthritis (osteo-, hip)	Y	Y	N	PY	Y	Y	Y	PY	Y	N	Y	N	Y	Y	Y	Y	Moderate
Arthritis (osteo-, knee)	Y	Y	N	PY	Y	Y	Y	Y	Y	N	Y	Y	Y	Y	Y	Y	Moderate
Arthritis (rheumatoid)	Y	N	N	N	Y	Y	PY	PY	Y	N	Y	N	Y	Y	Y	Y	Low
Asthma	Y	Y	N	N	Y	Y	PY	Y	Y/N	N	Y	N	Y	Y	N	Y	Critically low
Atrial fibrillation	Y	N	N	PY	N	N	N	PY	Y	N	Y	N	N	Y	Y	Y	Critically low
Bronchiectasis	Y	Y	N	PY	Y	Y	PY	N	Y	N	N	N	Y	N	N	N	Critically low
Cancer (solid tumour)	N	N	Y	PY	Y	Y	PY	N	Y	N	Y	Y	N	Y	Y	Y	Critically low
Cancer (haematological)	Y	Y	N	PY	Y	Y	Y	Y	Y	Y	Y	Y	Y	Y	N	Y	Moderate
Cancer (advanced metastatic)	Y	N	N	Y	Y	Y	PY	PY	Y	N	Y	Y	Y	Y	N	Y	Low
Chronic fatigue syndrome	Y	Y	N	PY	Y	Y	Y	Y	Y	Y	Y	Y	Y	Y	N	N	Moderate
Chronic kidney disease	Y	N	N	N	Y	N	PY	PY	PY	N	N	N	N	N	N	Y	Critically low
Chronic liver disease	Y	Y	N	Y	Y	Y	Y	Y	Y	Y	Y	N	Y	N	N	Y	Moderate
COPD	N	N	N	PY	N	Y	PY	N	Y	N	Y	N	N	Y	Y	Y	Critically low
Connective tissue disease	Y	Y	N	PY	Y	Y	Y	PY	Y	N	Y	Y	Y	Y	Y	Y	Moderate
Coronary heart disease	Y	Y	N	Y	Y	Y	Y	Y	Y	Y	Y	Y	Y	Y	Y	Y	High
Dementia	Y	N	N	PY	Y	N	PY	Y	PY	N	Y	Y	Y	Y	Y	Y	Low
Depression	Y	N	N	PY	Y	Y	Y	Y	PY	N	Y	N	Y	Y	Y	Y	Low
Diabetes mellitus	Y	Y	N	PY	Y	Y	Y	Y	Y	Y	Y	Y	N	Y	Y	Y	Moderate
Endometriosis	Y	Y	N	PY	Y	Y	N	PY	Y	N	NA	NA	Y	Y	NA	Y	Low
Epilepsy	Y	Y	N	Y	Y	N	Y	PY	Y	N	N	N	Y	Y	N	Y	Low
Glaucoma	N	N	N	N	Y	N	N	N	N	N	NA	NA	N	N	NA	Y	Critically low
Heart failure	Y	Y	N	Y	Y	Y	Y	Y	Y	Y	Y	Y	Y	Y	Y	Y	High
Hypertension	Y	N	N	N	N	N	Y	PY	Y	N	Y	N	N	N	Y	Y	Critically low
Inflammatory bowel disease	N	N	N	N	Y	Y	N	N	N	N	NA	NA	N	N	NA	Y	Critically low
Irritable bowel syndrome	N	N	N	N	Y	Y	N	N	Y	N	NA	NA	N	N	NA	Y	Critically low
Long-COVID	Y	PY	N	PY	Y	Y	PY	PY	Y	N	NA	NA	N	Y	NA	Y	Low
Migraine	Y	PY	N	PY	Y	Y	PY	PY	Y	N	N	N	Y	Y	Y	Y	Low
Multiple sclerosis	Y	Y	N	PY	Y	Y	PY	PY	Y	N	Y	N	N	Y	N	N	Low
Osteoporosis	Y	Y	N	PY	Y	N	PY	N	Y	N	Y	Y	Y	Y	N	Y	Moderate
Painful condition (chronic back pain)	Y	Y	N	Y	Y	Y	Y	Y	Y	Y	Y	Y	Y	Y	Y	Y	High
Painful condition (fibromyalgia)	Y	Y	N	Y	Y	Y	Y	Y	Y	Y	Y	Y	Y	Y	Y	Y	High
Parkinson’s disease	Y	PY	N	Y	Y	Y	Y	PY	PY	N	Y	N	N	N	N	Y	Critically low
Peripheral vascular disease	Y	Y	N	Y	Y	Y	Y	Y	Y	N	Y	N	Y	N	Y	Y	Moderate
Polycystic ovarian syndrome	Y	Y	N	PY	Y	N	Y	PY	Y	N	Y	Y	Y	Y	Y	Y	Moderate
Prostate disorders	Y	Y	N	Y	Y	Y	PY	Y	Y	N	Y	Y	Y	N	N	Y	Low
Psychoactive substance misuse	Y	PY	N	PY	Y	Y	PY	PY	N	N	N	N	Y	N	N	Y	Critically low
Schizophrenia	Y	Y	N	PY	Y	Y	PY	PY	Y	N	Y	Y	Y	Y	Y	N	Moderate
Stroke or TIA	Y	Y	N	Y	Y	Y	Y	Y	Y	N	Y	Y	Y	Y	Y	Y	High
Treated constipation	N	N	N	PY	Y	Y	PY	PY	Y	N	N	N	Y	Y	N	Y	Critically low

*Critical domains. Y: yes, PY partial yes; N: no; NA: not applicable; Y/N: yes (sub domain one), no (sub domain two)

8. Supplementary Figure 1 – Frequency of outcome reporting across included studies

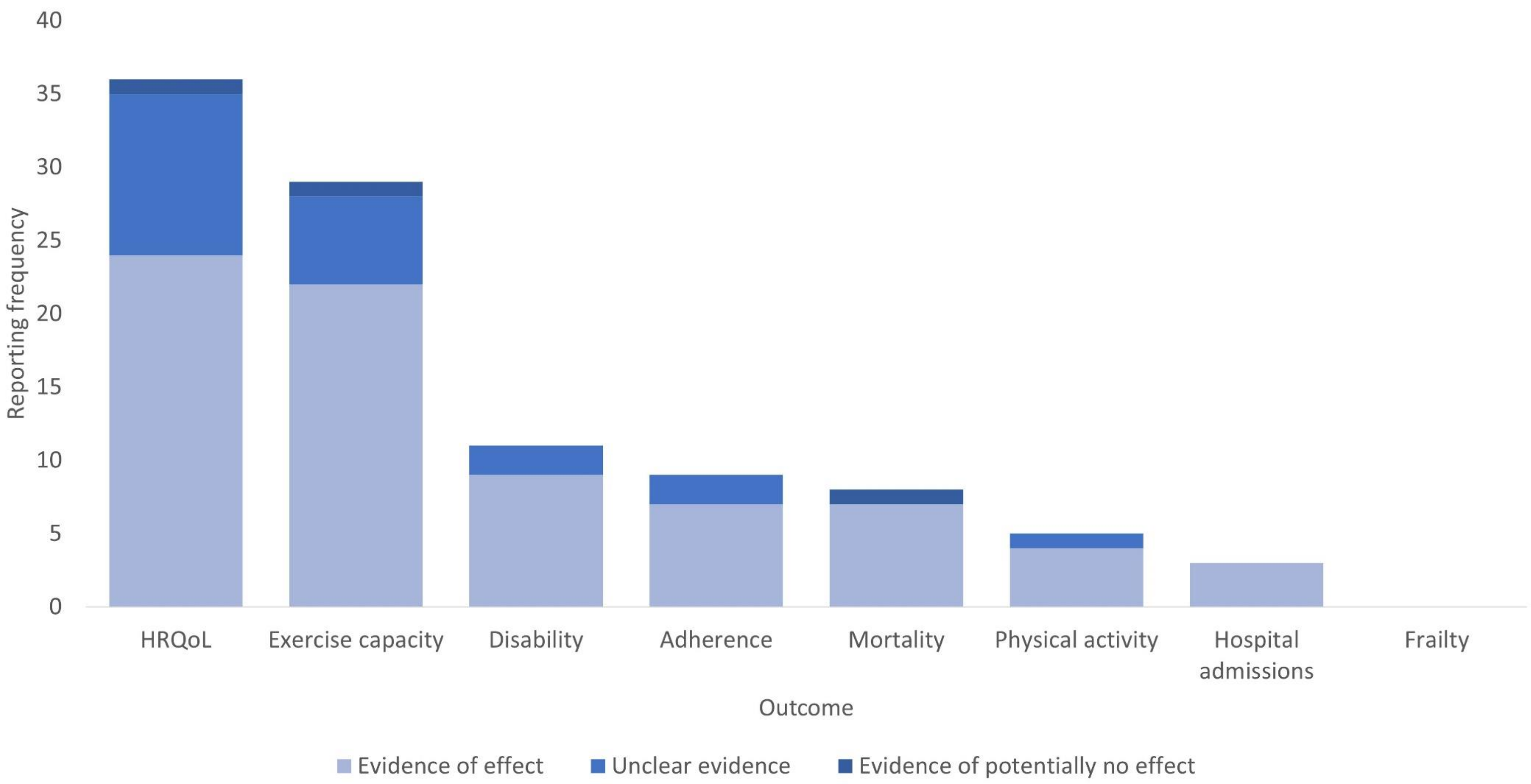

9. Supplementary Table 5 – Summary of exercise-based intervention vs control mortality by LTC

LTC	Effect estimates<br>*Follow-up*	N trials	N participants	N events<br>Intervention vs Control	Exercise vs control effect RR (95% CI)	I^2	P-value	GRADE rating*
Bronchiectasis	Risk ratio<br>*Follow-up NR*	1	NR	NR	3.0 (0.74 to 12.7)	NA	NR	NA
Cancer (haem)	Risk ratio<br>*Follow-up NR*	7	1172	97 vs 87	1.10 (0.79 to 1.52)	29%	0.59	Low
Chronic liver disease	Risk ratio<br>*Follow-up NR*	6	173	0 vs 2<br>(5 studies had 0 deaths in both groups)	0.19 (0.01 to 3.73)	NA	0.27	Moderate
Connective tissue disease	Odds ratio<br>*6-12 months*	4	291	5 vs 12	0.40 (0.14 to 1.12)	0%	0.08	Low
	Risk ratio†	4	291		0.41 (0.15 to 1.11)			
Coronary heart disease	Risk ratio<br>**All cause:**<br>*6-12 months*	26	8823	228 vs 242	0.87 (0.73 to 1.04)	0%	0.13	Moderate
	>12-36 months	16	11,073	467 vs 498	0.90 (0.80 to 1.02)	0%	0.10	NR
	>36 months	11	3828	476 vs 493	0.91 (0.75 to 1.10)	35%	0.34	NR
	**CV mortality:**<br>*6-12 months*	15	5360	109 vs 114	0.88 (0.68 to 1.14)	0%	0.34	NR
	>12-36 months	5	3614	199 vs 239	0.77 (0.63 to 0.93)	5%	0.008	NR
	>36 months	8	1392	56 vs 100	0.58 (0.43 to 0.78)	0%	0.0004	NR
Heart failure	Risk ratio<br>*<12 months*	27	2596	67 vs 75	0.89 (0.66 to 1.21)	0%	0.47	Low
	>12 months	6	2845	244 vs 280	0.88 (0.75 to 1.02)	34%	0.09	NR
Peripheral vascular disease	Risk ratio<br>*Follow-up NR*	5	540	8 vs 9	0.92 (0.39 to 2.17)	0%	0.85	Moderate
Stroke/TIA	Risk difference<br>**Cardiorespiratory training**<br>*End of intervention (months NR)*	32	1631	2 vs 2	0.0 (-0.01 to 0.01)	0%	NA	Low
	End of follow-up (months NR)	6	360	1 vs 1	0.0 (-0.03 to 0.03)	0%	NA	NR
	**Resistance training** *End of intervention*							
	End of follow-up	20	803	1 vs 1	0.0 (-0.02 to 0.02)	0%	0.99	NR
	**Mixed training**<br>*End of intervention*	5	251	2 vs 2	0.0 (-0.04 to 0.04)	0%	0.97	NR

	End of follow-up	23<br><br>13	1231<br><br>906	4 vs 9<br><br>4 vs 10	-0.0 (-0.02 to 0.01)<br><br>-0.01 (-0.03 to 0.01)	0%<br><br>0%	0.5<br><br>0.22	NR<br><br>NR

*GRADE rating as reported in SR. †Converted OR to RR by overview team. CV: cardiovascular; NA: not applicable; NR: not reported by SR authors

10. Supplementary Figure 2 – mortality forest plot comparing exercise-based intervention vs control

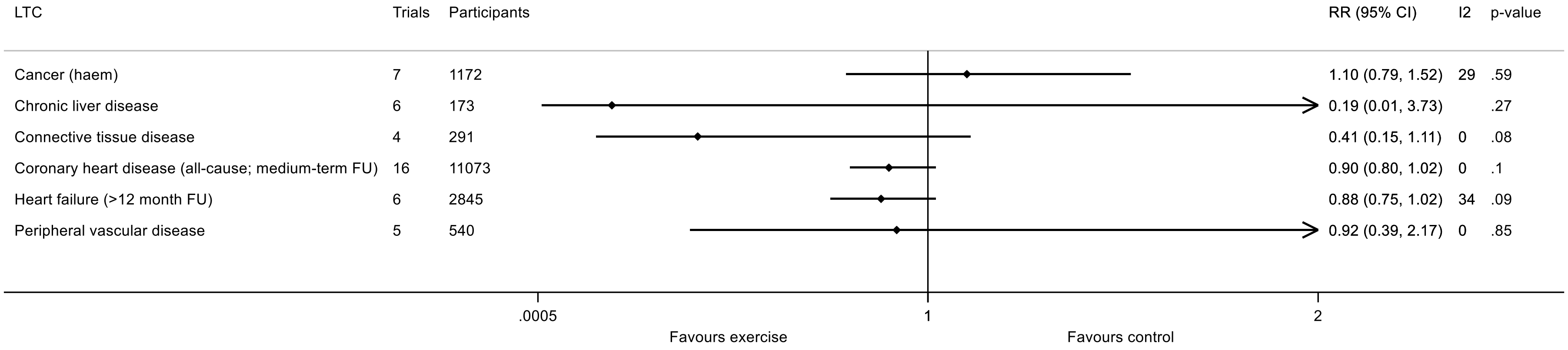

11. Supplementary Table 6 – Summary of exercise-based intervention vs control for hospital admissions by LTC

LTC	Effect estimate<br>*Follow-up*	N trials	N participants	N events<br>Intervention vs control	Exercise vs control effect RR (95% CI)	I^2	P-value	GRADE rating*
Bronchiectasis	Relative risk	1	NR	NR	1.5 (0.75 to 3.0)	NA	NR	NR
Coronary heart disease	Risk ratio<br>**All cause:**<br>*6-12 months*	14	2030	130 vs 209	0.58 (0.43 to 0.77)	42%	0.0001	Moderate
	>12-36 months	9	5995	392 vs 417	0.92 (0.82 to 1.03)	0%	0.14	NR
	**CV hospital admission:**<br>*6-12 months*	6	1087	40 vs 42	0.80 (0.41 to 1.59)	53%	0.53	Low
	>12-36 months	3	943	129 vs 141	0.92 (0.76 to 1.12)	0%	0.40	NR
Heart failure	Risk ratio<br>**All cause:**<br>*<12 months*	21	2182	180 vs 258	0.70 (0.60 to 0.83)	19%	0.001	Moderate
	>12 months	6	2691	772 vs 825	0.70 (0.47 to 1.05)	66%	0.08	NR
	**Disease specific:**<br>*<12 months*	14	1114	40 vs 61	0.59 (0.42 to 0.84)	11%	0.003	Low

*GRADE rating as reported in SR. NR: not reported by SR authors; CV: cardiovascular

12. Supplementary Table 7 – Summary of exercise-based intervention vs control for aerobic exercise capacity outcomes by LTC

LTC	Unit of analysis	N trials	N participants	Exercise vs control effect SMD (95% CI)	Exercise vs control effect MD (95% CI)	I^2	p-value	Vote counting* or qualitative statement by authors	GRADE rating†
**$VO_{2max/peak}$**									
Alcohol problems	SMD	3	NR	0.487 (0.144 to 0.830)	-	0%	0.005	-	NR
Anaemia	mL/kg/min	1	55	-	-	-	-	1/1 trial favours exercise	NR
Asthma	mL/kg/min	5	217	-	3.1 (1.9 to 4.3)	0%	<0.00001	-	NR
Atrial fibrillation	SMD	4	NR	0.37 (0.16 to 0.57)	-	0%	<0.001	-	NR
Cancer (solid tumour)	mL/kg/min	7	NR	-	2.2 (1.0 to 3.4)	18%	<0.01	-	NR
Chronic liver disease	mL/kg/min	4	100	-	0.30 (-2.74 to 3.35)	28%	0.85	-	Low
Connective tissue disease	mL/kg/min	3	94	-	1.28 (0.51 to 2.05)	57%	0.001	-	NR
Diabetes mellitus	mL/kg/min	3	95	-	4.84 (2.55 to 7.12)	76%	<0.0001	-	NR
Hypertension	mL/kg/min	9	NR	-	4.90 (2.71 to 7.09)	87%	<0.001	-	NR
Multiple sclerosis	SMD (aerobic studies only)	7	366	0.88 (0.25 to 1.50)	-	78%	0.001	-	NR
Parkinson's disease	SMD & mL/kg/min<br>Aerobic studies only<br><br>Resistance studies only	 6<br><br>3	 427<br><br>75	 0.27 (0.07 to 0.47)<br><br>-0.10 (-0.56 to 0.36)	 1.76 (0.43 to 3.08)<br><br>NR	 0%<br><br>0%	 0.008<br><br>0.67	-	 NR<br><br>NR
Polycystic ovarian syndrome	SMD & mL/kg/min post-intervention<br><br>change from baseline	6<br><br>6	184<br><br>229	1.19 (0.40 to 1.99)<br><br>1.43 (0.84 to 2.03)	5.01 (3.48 to 6.54)<br><br>3.84 (2.87 to 4.81)	42%<br><br>17%	≤0.001<br><br>≤0.001	-	NR<br><br>NR
Prostate disorders	SMD	3	220	0.27 (-0.00 to 0.54)	-	0%	0.05	-	NR
Schizophrenia	SMD & mL/kg/min	7	NR	0.36 (0.08 to 0.63)	1.66 (0.49 to 2.83)	61%	0.011	-	NR
Stroke/TIA	mL/kg/min<br>Cardiorespiratory training (end of intervention)<br><br>Mixed training (end of intervention)	 9<br><br>2	 438<br><br>140	-	 3.40 (2.98 to 3.83)<br><br>1.40 (-0.19 to 2.99)	 0%<br><br>35%	 <0.0001<br><br>0.08	-	 Moderate<br><br>Low
Viral hepatitis	mL/kg/min	1	22	-	-	-	-	1/1 trial favours exercise	NR
**Other aerobic/overall**									
Anaemia	Peak workload (Watts)	1	55	-	-	-	-	1/1 trial favours exercise	NR
Anxiety	"Fitness levels"	7	NR	-	-	NA	NA	4/7 (57%) trials favour exercise<br>3/7 (45%) neutral	NR
Asthma	Overall health-related physical fitness	12	477	0.67 (95% CI 0.46 to 0.89)	-	16%	p<0.0001	-	NR

Cancer (solid tumour)	Peak power output (W)	3	NR	-	21.0 (13.0 to 29.1)	0%	<0.01	-	NR
Cancer (haem)	Aerobic capacity/CV fitness measures	8	NR	-	-	-	-	11/14 (79%) results favour exercise 3/14 (21%) neutral	NR
Connective tissue disease	Peak work rate (W)	4	159	-	9.04 (6.07 to 12.00)	89%	<0.00001	-	Low
Prostate disorders	Submaximal aerobic capacity	6	346	0.49 (0.12 to 0.85)	-	61%	0.01	-	NR
Psychoactive substance abuse	Aerobic fitness	1	-	-	-	-	-	"As assessments and interventions varied greatly between each study, it is not possible to observe any trends in results"	NR
Schizophrenia	Peak power output (SMD & Watts)	2	NR	0.11 (-0.29 to 0.51)	25.01 (-67.93 to 117.95)	0%	0.604	-	NR
Stroke/TIA	Max cycling work rate (watts)	6	336	-	10.60 (1.88 to 19.33)	85%	0.02	-	NR

*'favours exercise': exercise better than control (P≤0.05); neutral: 'no difference' between exercise and control (P>0.05); 'favours control': control better than exercise (P≤0.05). †GRADE rating as reported in SR
MD: mean difference; SMD: standardised mean difference; CI: confidence interval; NR: not reported

13. Supplementary Figure 3 – Forest plot showing meta-analysis results for VO2max/peak. Panel A shows results reported as ml/min/kg with mean differences and as standardised mean differences on panel B.

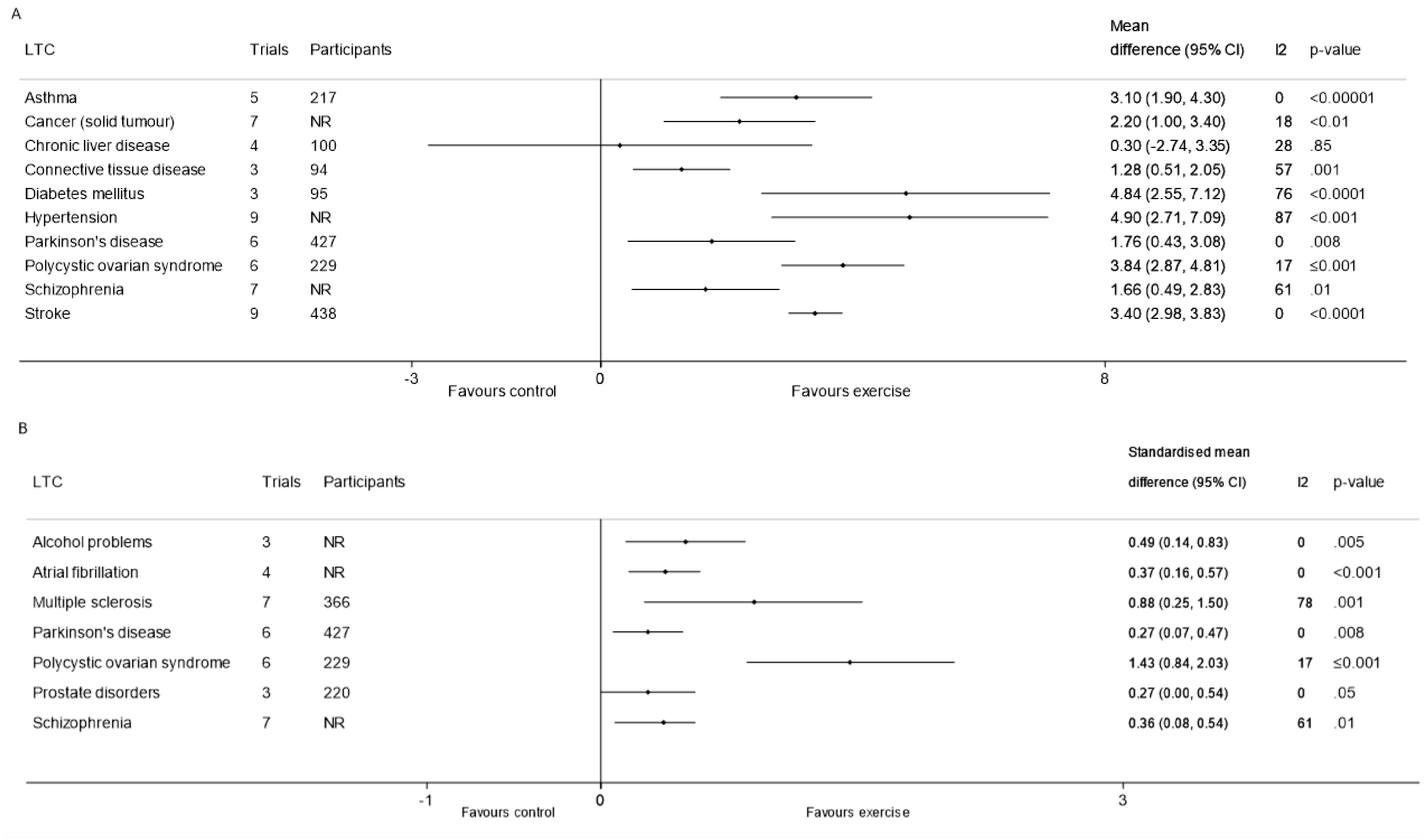

14. Supplementary Table 8 – Summary of exercise-based intervention vs control for functional exercise capacity outcomes by LTC

LTC	Unit of analysis	N trials	N participants	Exercise vs control effect SMD (95% CI)	Exercise vs control effect MD (95% CI)	I^2	p-value	Vote counting* or qualitative statement by authors	GRADE rating†
**6MWT**									
Atrial fibrillation	SMD	5	NR	0.69 (0.19 to 1.19)	-	70.6%	0.007	-	NR
Cancer (solid tumour)	m	5	NR	-	29 (4 to 55)	20%	0.03		NR
Chronic Kidney disease	SMD (aerobic)<br><br>m (combination training)	2<br><br>2	61<br><br>69	0.99 (0.45 to 1.54)	 <br>63.56 (43.44 to 83.68)	64%<br><br>0%	0.003<br><br><0.0001	-	NR
Chronic liver disease	m	4	105	-	56.06 (-9.14 to 121.26)	64%	0.09	-	Very low
COPD	m	34	NR	-	36.34 (26.51 to 46.17)	92%	<0.001	-	NR
Connective tissue disease	m, post-intervention<br><br>m, long-term FU	13<br><br>5	585<br><br>297	-	40.07 (32.70 to 47.44)<br><br>32.43 (15.58 to 49.28)	26%<br><br>43%	<0.00001<br><br>0.0002	-	Moderate<br><br>Moderate
Dementia	m	7	402	-	50 (18 to 81)	79%	0.002	-	Moderate
Long-COVID	post-intervention<br><br>follow-up	1<br><br>1	120<br><br>120	-	-	-	-	1/1 trial favours exercise<br>1/1 trial favours exercise	NR
Painful condition (fibromyalgia)	m<br>short-term FU<br><br>long-term FU	 5<br><br>3	 306<br><br>145	-	 52.77 (34.11 to 71.43)<br><br>61.71 (15.37 to 108.1)	 0%<br><br>59%	 <0.0001<br><br>0.01	-	 NR<br><br>NR
Parkinson's disease	SMD and m	5	250	0.89 (0.17 to 1.62)	69.2 (28.9 to 109.5)	83%	0.02	-	NR
Schizophrenia	SMD and m	3	NR	0.86 (0.15 to 1.56)	67.41 (36.21 to 98.62)	50%	0.018	-	NR
Stroke/TIA	m<br>cardiorespiratory training (end of intervention)<br><br>cardiorespiratory training (end of FU)<br><br>resistance training (end of intervention)<br><br>resistance training (end of FU)<br><br>mixed training (end of intervention)	 16<br><br>5<br><br>5<br><br>2<br><br>10	 882<br><br>283<br><br>238<br><br>117<br><br>720	-	 33.41 (19.04 to 47.78)<br><br>38.29 (7.19 to 69.39)<br><br>24.98 (11.98 to 37.98)<br><br>22.41 (-27.87 to 72.69)<br><br>35.00 (15.91 to 54.09)	 31%<br><br>32%<br><br>0%<br><br>0%<br><br>28%	 <0.0001<br><br>0.02<br><br>0.00<br><br>0.38<br><br>0.00	-	 High<br><br>NR<br><br>Low<br><br>NR<br><br>Low

	mixed training (end of FU)	4	464		47.48 (23.72 to 71.23)	0%	<0.0001		NR
Viral hepatitis	m	1	40	-	-	-	-	1/1 neutral	NR
**Other functional/walking tests**									
Arthritis (rheumatoid)	50ft walk test (SMD)	4	134	-0.64 (-0.99 to -0.28)	-	0%	NR	-	NR
Asthma	Walking distance (m)	7	139	-	40.5 (26.6 to 54.5)	0%	<0.00001	-	NR
Bronchiectasis	ISWT (m)	3	135	-	66.52 (51.57 to 81.68)	38%	<0.00001	-	NR
Cancer (solid tumour)	Sit and reach (cm)	2	NR	-	2 (-3 to 8)	0%	0.36	-	NR
Cancer (haem)	6MWD, 12 min walking test, balance, sit-to-stand - narrative	4	NR	-	-	-	-	5/6 results favour exercise<br>1/6 (17%) neutral	NR
Dementia	Timed-up-and-go (s)<br><br>Walking speed (m/s)	11<br><br>7	606<br><br>568	-	-1.0 (-2 to 0)<br><br>0.13 (0.03 to 0.24)	18%<br><br>90%	0.05<br><br>0.01	-	Moderate<br><br>Moderate
Multiple sclerosis	Short walking test (aerobic trials only)<br><br>Short walking test (resistance trials only)<br><br>Long walking test (aerobic trials only)<br><br>Long walking test (resistance trials only)	3<br><br>5<br><br>7<br><br>4	124<br><br>132<br><br>291<br><br>169	0.33 (-1.49 to 2.06)<br><br>0.27 (0.07 to 0.47)<br><br>0.37 (-0.04 to 0.78)<br><br>0.36 (-0.35 to 1.08)	-	69%<br><br>0%<br><br>43%<br><br>48%	0.202<br><br>0.000<br><br>0.026<br><br>0.108	-	NR<br><br>NR<br><br>NR<br><br>NR
Osteoporosis	Mobility	16	1065	-0.72 (-1.00 to -0.44)	-	77%	<0.00001	-	NR
Parkinson's disease	Timed up-and-go (SMD and s)<br>Aerobic studies only<br><br>Resistance studies only	 6<br><br>8	 255<br><br>309	 -0.21 (-0.46 to 0.04)<br><br>-0.62 (-1.01 to -0.24)	 -0.51 (-1.16 to 0.15)<br><br>-1.45 (-2.56 to -0.34)	 0%<br><br>53%	 0.1<br><br>0.002	-	 NR<br><br>NR
Peripheral vascular disease	Maximum walking distance (m)<br><br>Pain-free walking distance (m)<br><br>Max walking time (min)	10<br><br>9<br><br>12	500<br><br>391<br><br>577	-	120.36 (50.79 to 189.92)<br><br>82.11 (71.73 to 92.48)<br><br>4.51 (3.11 to 5.92)	89%<br><br>40%<br><br>82%	0.00<br><br><0.0001<br><br><0.0001	-	High<br><br>High<br><br>High
Psychoactive substance abuse	Functional capacity	2	-	-	-	-	-	"As assessments and interventions varied greatly between each	NR

								study, it is not possible to observe any trends in results”	
Stroke/TIA	**Functional ambulation categories**							-	
	Cardiorespiratory training (end of intervention)	2	73		0.53 (0.21 to 0.85)	0%	0.00		NR
	**Walking maximal speed (over 5-10m)**								
	Cardiorespiratory training (end of intervention)	17	782		7.66 (3.65 to 11.68)	66%	0.00		NR
	**Walking preferred speed (m/min)**								
	Cardiorespiratory training (end of intervention)	12	588		4.47 (2.07 to 6.87)	0%	0.00		High
	Cardiorespiratory training (end of FU)	3	176		1.67 (-3.27 to 6.62)	0%	0.51		NR
	Resistance training (end of intervention)	5	203		2.15 (-3.57 to 7.87)	76%	0.46		oderate
	Mixed training (end of intervention)	10	738		4.71 (1.32 to 8.10)	76%	0.01		Moderate
	Mixed training (end of FU)	5	542		2.54 (-3.65 to 8.72)	77%	0.42		NR
	**Walking capacity (m/min)**								
	Cardiorespiratory training (end of intervention)	3	154		8.87 (1.35 to 16.40	42%	0.02		NR
	Cardiorespiratory training (end of FU)	5	312		6.71 (2.40 to 11.02)	0%	0.00		NR
	Resistance training (end of intervention)	7	274		2.83 (-0.49 to 6.14)	54%	0.1		NR
	Resistance training (end of FU)	2	117		7.80 (-3.32 to 18.91)	0%	0.17		NR

	Mixed training (end of intervention)	3	168		8.48 (1.76 to 15.20)	0%	0.01		NR
	**Community walk (min)** Cardiorespiratory training (end of intervention)	2	47		-10.54 (-14.11 to 6.98)	0%	<0.0001		NR
	**Community ambulation speed (>0.8 m/sec, OR)** Mixed training (end of intervention)	3	232		1.38 (0.78 to 2.42)	0%	0.26		NR
	Mixed training (end of FU)	3	217		1.33 (0.70 to 2.53)	15%	0.38		NR
	**Timed up-and-go (sec)** Cardiorespiratory training (end of intervention)	5	223		-3.42 (-4.78 to -2.05)	0%	<0.0001		Moderate
	Resistance training (end of intervention)	5	224		-3.46 (-6.94 to 0.02)	89%	0.05		Low
	Resistance (end of FU)	2	117		-2.64 (-9.24 to 3.95)	0%	0.43		NR
	Mixed training (end of intervention)	7	586		-2.21 (-4.43 to 0.02)	45%	0.05		Low
	Mixed training (end of FU)	5	510		-1.41 (-3.74 to 0.92)	47%	0.24		NR

*'favours exercise': exercise better than control (P≤0.05); neutral: 'no difference' between exercise and control (P>0.05); 'favours control': control better than exercise (P≤0.05). †GRADE rating as reported in SR
MD: mean difference; SMD: standardised mean difference; CI: confidence interval; NR: not reported; FU: follow-up

15. Supplementary Figure 4 – Forest plot showing meta-analysis results for 6MWT (m).

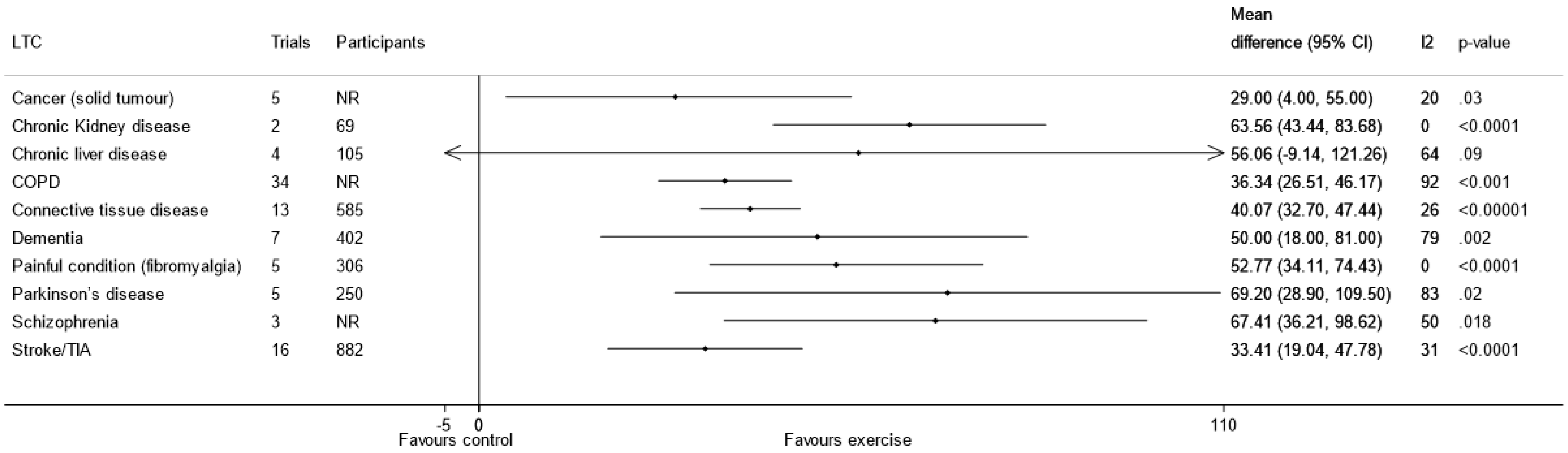

16. Supplementary Table 9 – Summary of exercise-based intervention vs control for strength outcomes by LTC

LTC	Unit of analysis	N trials	N participants	Exercise vs control effect SMD (95% CI)	Exercise vs control effect MD (95% CI)	I^2	p-value	Vote counting* or qualitative statement by authors	GRADE rating†
Anorexia	Strength (narrative, peak torque of knee extensors, knee and elbow flexors)	1	NR	-	-	NA	NA	3/3 results favour exercise	NR
Asthma	Muscular fitness	3	-	-	-	-	-	2/3 trials favour exercise<br>1/3 neutral	NR
Bronchiectasis	Quadriceps muscle strength	1	NR	-	-	-	-	1/1 trial neutral	NR
Cancer (solid tumour)	Kg (1 rep max):<br>Bench press<br>Leg press<br>Left handgrip<br>Right handgrip	 3<br>3<br>3<br>5	 NR<br>NR<br>NR<br>NR	-	 6 (4 to 8)<br>19 (9 to 28)<br>4.3 (-1.5 to 10.2)<br>3.5 (0.3 to 6.7)	 54%<br>71%<br>71%<br>56%	 <0.01<br><0.01<br>0.15<br>0.03	-	 NR<br>NR<br>NR<br>NR
Cancer (haem)	Strength change, muscle strength, bicep curl, knee extension, grip strength	4	NR	-	-	-	-	4/6 results favour exercise<br>2/6 (43%) neutral	NR
Dementia	30s sit-to-stand (repetitions)<br>Other types of sit-to-stand test<br>Upper limb strength	4<br>4<br>4	278<br>NR<br>NR	-	2.1 (0.3 to 3.9)<br>-<br>-	82%	0.02	 4/4 trials favour exercise<br>1/4 (25%) trials favour exercise<br>3/4 (75%) neutral	Moderate
Hypertension	Muscle strength (SMD)	9	NR	1.10 (0.66 to 1.55)	-	73%	<0.01	-	NR
Long-COVID	30s sit-to-stand<br>Static quat test at the wall (post-intervention)<br>Static quat test at the wall (FU)	1<br>1<br>1	196<br>120<br>120	-	-	-	-	1/1 trial favour exercise<br>1/1 trial favour exercise<br>1/1 trial favour exercise	NR
Multiple Sclerosis	Muscle strength (resistance trials only)	7	214	0.86 (0.02 to 1.70)	-	75%	0.013	-	NR

Osteoporosis	Leg strength	6	349	2.02 (0.71 to 3.33)	-	95%	0.002	-	NR
	Trunk strength	2	48	0.60 (0.01 to 1.18)		0%	0.04		NR
	Grip strength	4	380	0.43 (0.18 to 0.67)		29%	0.0007		NR
Painful condition (fibromyalgia)	Muscle strength	4	163	0.74 (-0.67 to 2.14)	-	93%	0.3	-	NR
Parkinson's disease	Muscle strength	7	211	0.83 (0.54 to 1.12)	-	0%	<0.001	-	NR
Prostate disorders	Upper body	4	277	0.26 (0.02 to 0.51)	-	0%	0.04	-	NR
	Lower body	6	245	0.29 (0.07 to 0.50)		0%	0.01		NR
Psychoactive substance abuse	Strength	2	-	-	-	-	-	"As assessments and interventions varied greatly between each study, it is not possible to observe any trends in results"	NR
Stroke/TIA	**Composite measure of muscle strength**<br>Resistance training (end of intervention)	2	60	0.58 (0.06 to 1.10)		0%	0.03	-	Low
	**Paretic knee flexion**<br>Resistance training (end of intervention)	3	93	0.72 (0.10 to 1.34)		47%	0.02		Moderate
	**Paretic knee extension**<br>Resistance training (end of intervention)	3	93	1.09 (-0.23 to 2.41)		87%	0.10		Low
	Mixed training (end of intervention)	3	202	0.33 (0.05 to 0.61)		0%	0.02		Low
	**Ankle dorsiflexion**<br>Mixed training (end of intervention)	2	148	0.80 (-0.82 to 2.41)		94%	0.33		Very low
	**Grip strength**<br>Mixed training (end of intervention)	3	147	0.32 (-0.88 to 1.52)		0%	0.60		Low
Viral hepatitis	Grip strength	1	22	-	-	-	-	1/1 trial favour exercise	NR
	5 times sit-to-stand test	1	22					1/1 trial favour exercise	

*’favours exercise’: exercise better than control (P≤0.05); neutral: ‘no difference’ between exercise and control (P>0.05); ‘favours control’: control better than exercise (P≤0.05). †GRADE rating as reported in SR
MD: mean difference; SMD: standardised mean difference; CI: confidence interval; NR: not reported; FU: follow-up

17. Supplementary Figure 5 – Forest plot showing meta-analysis results for strength (standardised mean difference).

LTC	Trials	Participants	Standardised mean difference (95% CI)	I2	p-value
Hypertension	9	NR	1.10 (0.66, 1.55)	73	<0.01
Multiple sclerosis	7	214	0.86 (0.02, 1.70)	75	.013
Osteoporosis	4	380	0.43 (0.18, 0.67)	29	.0007
Painful condition (fibromyalgia)	4	163	0.74 (-0.67, 2.14)	93	.3
Parkinson's disease	7	211	0.83 (0.54, 1.12)	0	<0.001
Prostate disorders	4	277	0.26 (0.02, 0.51)	0	.04
Stroke	3	202	0.33 (0.05, 0.61)	0	.02

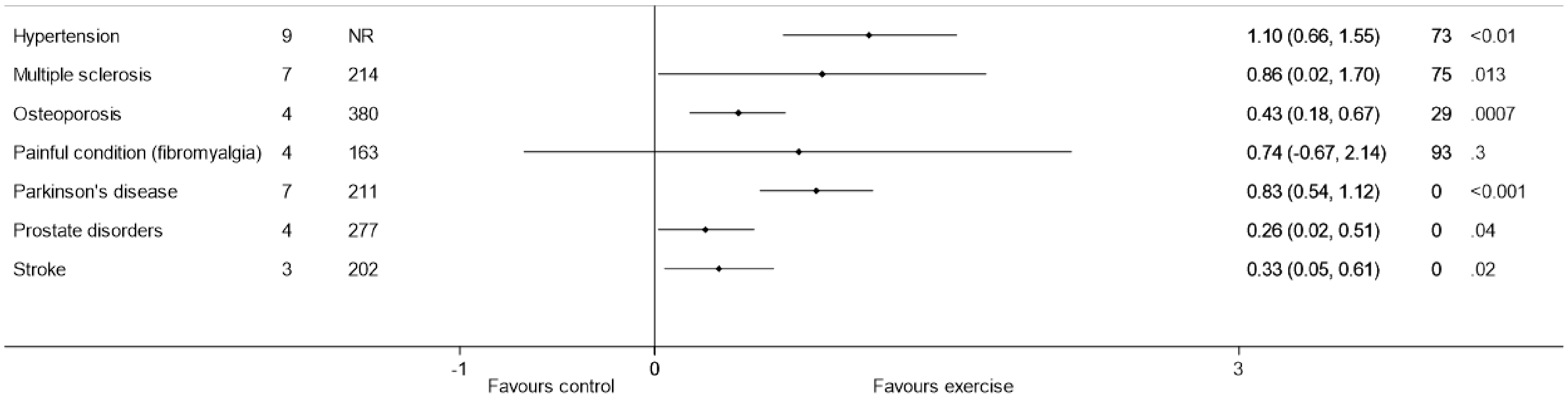

18. Supplementary Table 10 – Summary of exercise-based intervention vs control for disability outcomes by LTC

LTC	Questionnaire	N trials	N participants	Exercise vs control effect SMD (95% CI)	Exercise vs control effect MD (95% CI)	I^2	p-value	Vote counting*	GRADE rating†
Arthritis (osteo-hip)	WOMAC/GARS Physical function post intervention	9	521	0.30 (-0.54 to -0.05)	-	41%	0.02	-	High
	3-6 month FU	5	365	-0.37 (-0.57 to -0.16)		0%	0.0006		High
Arthritis (osteo-, knee)	WOMAC/VAS Physical function post treatment	44	3913	-0.52 (-0.64 to -0.39)	-	68%	<0.00001	-	Moderate
	2-6 month FU	10	1468	-0.15 (-0.26 to -0.04)		0%	<0.01		NR
	>6 month FU	8	1266	-0.57 (-1.05 to -0.1)		90%	<0.02		NR
Arthritis (rheumatoid)	HAQ	13	745	-	-0.10 (-0.26 to 0.06)	84%	<0.001	-	NR
COPD	MRC dyspnoea scale	11	NR	-	-0.59 (-0.81 to -0.37)	77%	<0.001	-	NR
Dementia	Barthel Index Score	4	237	-	10 (3 to 16)	89%	0.004	-	NR
Migraine	6-item headache impact test; pain disability index	2	NR	-	-	-	-	2/2 trials favour exercise	NR
Painful condition (chronic back pain)	Functional limitations – Roland-Morris Disability Questionnaire or Oswestry Disability Index			-				-	
	Overall – earliest FU	38	2942		-6.8 (-8.3 to -5.3)	38%	<0.00001		Moderate
	Short-term FU (3-months)	30	2555		-7.4 (-9.2 to -5.6)	43%	<0.00001		NR
	Medium-term FU (6 months)	22	1831		-6.6 (-8.3 to -4.8)	21%	<0.00001		NR
	Long-term FU (12 months)	6	550		-4.4 (-7.3 to -1.5)	0%	0.003		NR
Painful condition (fibromyalgia)	FIQ – physical function short-term FU	9	477	-	-10.99 (-14.8 to -7.18)	12%	<0.0001	-	NR
	Long-term FU (13-26 weeks)	3	179		-8.13 (-18.24 to 1.97)	63%	0.11		NR
Parkinson's disease	UPDRS III 'On-medication state'	6	277	-0.15 (-0.38 to 0.09)	-2.33 (-5.16 to 0.49)	0%	0.23	-	NR
	'Off-medication state'	4	320	-0.19 (-0.41 to 0.04)	-2.62 (-5.64 to 0.41)	0%	0.10		NR

Schizophrenia	Global functioning	10	NR	0.35 (0.16 to 0.54)	-	38%	<0.001	-	NR
Stroke/TIA	**Functional independence measure**							-	
	Cardiorespiratory training (end of intervention)	3	162	0.21 (-0.10 to 0.52)		0%	0.18		NR
	**Barthel index**								
	Cardiorespiratory training (end of intervention)	3	243		6.65 (-0.67 to 13.98)	94%	0.08		NR
	Mixed training (end of intervention)	6	256		2.84 (-0.48 to 6.17)	2	0.09		NR
	Mixed training (end of FU)	2	103		1.82 (-13.69 to 17.33)	68%	0.82		NR
	**Rivermead mobility index**								
	Cardiorespiratory training (end of intervention)	3	146		1.56 (0.20 to 2.92)	43%	0.02		NR
	Mixed training (end of intervention)	3	348		0.41 (-0.02 to 0.84)	23%	0.06		NR
	Mixed training (end of FU)	3	349		0.35 (0.02 to 0.69)	0%	0.04		NR
	**Combined disability scales**								
	Cardiorespiratory training (end of intervention)	8	462	0.52 (0.19 to 0.84)		61%	0.00		Moderate
	Cardiorespiratory training (end of FU)	3	220	0.20 (-0.07 to 0.46)		0%	0.14		NR
	Mixed training (end of intervention)	9	604	0.23 (0.03 to 0.42)		21%	0.02		Low
	Mixed training (end of FU)	5	452	0.10 (-0.17 to 0.37)		40%	0.45		NR
	**Lawton IADL**								
	Mixed training (end of intervention)	2	113		0.83 (-0.51 to 2.17)	0%	0.22		NR

	**Nottingham Extended ADL** Mixed training (end of FU)	2	106		3.10 (-5.20 to 11.40)	60%	0.46		NR

*'favours exercise': exercise better than control (P≤0.05); neutral: 'no difference' between exercise and control (P>0.05); 'favours control': control better than exercise (P≤0.05). †GRADE rating as reported in SR

MD: mean difference; SMD: standardised mean difference; CI: confidence interval; NR: not reported; FU: follow-up

19. Supplementary Table 11 – Summary of exercise-based intervention vs control for disease specific HRQoL outcomes by LTC

LTC	Questionnaire	N trials	N participants	Exercise vs control effect SMD (95% CI)	Exercise vs control effect MD (95% CI)	I^2	p-value	Vote counting*	GRADE rating†
Anaemia	**FACT-An**	1	55	-	-3.2 (-16.7 to 10.4)	NA	0.637	-	NR
Bronchiectasis	**SGRQ**	2	91	-	-4.65 (-6.70 to -2.60)	34%	<0.00001	-	NR
	**LCQ**	2	103		1.27 (-0.89 to 3.42)	77%	0.25		NR
Cancer (solid tumour)	**EORTC:**			-				-	NR
	Physical function	2	NR		-1.2 (-5.1 to 2.6)	0%	0.54		
	Emotional function	3	NR		1.5 (-9.8 to 12.7)	77%	0.80		NR
	Total	3	NR		3.7 (-5.2 to 12.6)	70%	0.41		NR
	**FACT:**								
	Physical wellbeing	6	NR		1.4 (-1.7 to 4.5)	92%	0.39		NR
	Emotional wellbeing	6	NR		0.7 (-0.6 to 1.9)	61%	0.31		NR
	Social/family wellbeing	6	NR		0.7 (-0.2 to 1.6)	15%	0.14		NR
	Functional wellbeing	6	NR		1.9 (-1.9 to 5.8)	93%	0.33		NR
	Breast	4	NR		1.9 (-1.6 to 5.4)	78%	0.28		NR
	Breast total	10	NR		7.6 (0.6 to 14.5)	86%	0.03		NR
	Colorectal total	3	NR		-1.6 (-6.2 to 2.9)	0%	0.48		NR
	General total	7	NR		4.2 (-3.1 to 11.5)	92%	0.26		NR
Chronic kidney disease	**KDQOL-SF 1.3**	3	NR	-	-	-	-	3/3 (100%) in favour of exercise	NR
Chronic liver disease	**CLDQ**	3	81	-	0.11 (-0.44 to 0.67)	33%	0.69	-	Low
COPD	**SGRQ**	25	NR	-	-6.66 (-8.38 to -4.94)	78%	<0.001	-	NR
Connective tissue disease	**SGRQ**			-				-	
	symptoms score (post-intervention)	7	312		-15.58 (-19.54 to -11.62)	52%	<0.00001		NR
	activity score (post-intervention)	7	312		-2.47 (-4.11 to -0.83)	54%	0.003		NR
	impact score (post-intervention)	7	312		-8.81 (-11.17 to -6.46)	0%	<0.00001		NR
	total score	11	478		-9.29 (-11.06 to -7.52)	27%	<0.00001		Moderate

	(post-intervention)<br><br>symptoms score<br>(long-term FU)<br><br>activity score<br>(long-term FU)<br><br>impact score<br>(long-term FU)<br><br>total score<br>(long-term FU)	 <br>4<br><br><br>4<br><br><br>4<br><br><br>4	 <br>240<br><br><br>240<br><br><br>240<br><br><br>240		 <br>-11.31 (-16.58 to -6.03)<br><br><br>-1.54 (-3.11 to 0.02)<br><br><br>-4.73 (-7.76 to -1.69)<br><br><br>-4.93 (-7.81 to -2.06)	 <br>0%<br><br><br>56%<br><br><br>60%<br><br><br>63%	 <br><0.0001<br><br><br>0.05<br><br><br>0.002<br><br><br>0.0008		 <br>NR<br><br><br>NR<br><br><br>NR<br><br><br>Low
Endometriosis	**EHP-30**	1	NR	-	-	-	-	1/1 trial favours exercise	NR
Glaucoma	**Visual quality of life**	1	NR	-	-	-	-	1/1 trial favours exercise	NR
Heart failure	**MLWHFQ**<br>6-12 month FU<br><br>>12 month FU	 17<br><br>3	 1995<br><br>329	-	 -7.11 (-10.49 to -3.73)<br><br>-9.49 (-17.48 to -1.50)	 82%<br><br>73%	 <0.0001<br><br><0.02	-	 Low<br><br>Low
Inflammatory bowel disease	**IBDQ**	6	NR	-	-	-	-	5/6 trials favour exercise 1/6 neutral	NR
Irritable bowel syndrome	**IBS-QOL**	4	NR	-	-	-	-	3/4 trials favour exercise 1/4 neutral	NR
Migraine	**Migraine specific QoL questionnaire**	1	NR	-	-	-	-	1/1 trial neutral	NR
Painful condition (fibromyalgia)	**FIQ**<br>short-term FU<br><br>long-term FU (13-26 weeks)<br><br>long-term FU (27-52 weeks)	 13<br><br>4<br><br>2	 610<br><br>224<br><br>146	-	 -6.95 (-10.51 to -3.38)<br><br>-8.44 (-15.22 to -1.66)<br><br>-5.29 (-11.42 to 0.84)	 51%<br><br>56%<br><br>0%	 0.00<br><br>0.01<br><br>0.09	-	 Moderate<br><br>NR<br><br>NR
Parkinson’s disease	**PDQ-39**<br>Resistance studies only	5	165	-0.41 (-0.72 to -0.09)	-5.4 (-10.5 to -0.3)	0%	<0.01	-	NR
Prostate disorders	Cancer-specific QoL	7	912	0.13 (-0.08 to 0.34)	-	46%	0.23	-	NR
Psychoactive substance abuse	**QOL DA**	1	NR	-	-	-	-	2/5 results favour exercise 3/5 neutral	NR

Viral hepatitis	**CLDQ**	1	40	-	-	-	-	1/7 results favour exercise 6/7 neutral	NR

*'favours exercise': exercise better than control (P≤0.05); neutral: 'no difference' between exercise and control (P>0.05); 'favours control': control better than exercise (P≤0.05). †GRADE rating as reported in SR

MD: mean difference; SMD: standardised mean difference; CI: confidence interval; NR: not reported; FU: follow-up

CLDQ = chronic liver disease questionnaire; EHP-30 = Endometriosis health profile-30; EORTC = European Organisation for Research and Treatment of Cancer; FACT = functional assessment of cancer therapy; FIQ = fibromyalgia impairment questionnaire; IBDQ = Inflammatory bowel disease questionnaire; IBS-QOL = irritable bowel syndrome quality of life; KDQOL-SF = kidney disease quality of life short form; MLWHFQ = Minnesota Living with Heart Failure Questionnaire; PDQ-39 = Parkinson's disease questionnaire; QOL DA = quality of life scale for drug addiction; SGRQ = St George Respiratory Questionnaire;

20. Supplementary Table 12 – Summary of exercise-based intervention vs control for generic HRQoL outcomes by LTC

LTC	Questionnaire Subscale	N trials	N participants	Exercise vs control effect SMD (95% CI)	Exercise vs control effect MD (95% CI)	I^2	p-value	Vote counting*	GRADE rating†
Arthritis (osteo-, hip)	SF-36/VAS - SMD	3	183	0.10 (-0.23 to 0.36)	-	0%	0.66	-	Low
Arthritis (osteo-, knee)	SMD (mix of generic and disease-specific questionnaires)	13	1073	0.28 (0.15 to 0.40)	-	0%	<0.00001	-	High
Atrial fibrillation	**SF-36:**				-			-	NR
	physical functioning	8	632	0.63 (0.18 to 1.09)		85.6%	<0.001		
	general health	8	632	0.64 (0.35 to 0.93)		66.2%	0.004		
	vitality	8	632	0.51 (0.31 to 0.71)		32%	0.172		
Cancer (solid tumour)	**SF-36:**			-				-	
	Physical function	2	NR		3.0 (0.7 to 5.3)	0%	0.01		NR
	Role physical	2	NR		6.1 (-4.1 to 16.2)	56%	0.24		NR
	Bodily pain	2	NR		4.3 (-0.6 to 9.2)	54%	0.08		NR
	General Health	2	NR		5.8 (-1.2 to 13.0)	76%	0.12		NR
	Vitality	2	NR		2.1 (-0.4 to 4.5)	0%	0.10		NR
	Social function	2	NR		3.4 (0.4 to 6.4)	0%	0.03		NR
	Role emotion	2	NR		-0.01 (-3.7 to 3.6)	0%	1.00		NR
	Mental Health	2	NR		2.4 (0.7 to 4.1)	0%	0.01		NR
	Physical component scale	4	NR		1.2 (-1.9 to 4.4)	55%	0.44		NR
	Mental component scale	4	NR		0.4 (-2.3 to 3.2)	39%	0.76		NR
Cancer (haem)	**Not reported**				-			-	
	overall	8	1259	0.11 (-0.03 to 0.24)		26%	0.14		Low
	physical functioning	8	1329	0.15 (-0.01 to 0.32)		48%	0.06		Low
	depression	6	445	0.19 (0.0 to 0.38)		0%	0.05		Low
	anxiety	6	445	0.03 (-0.30 to 0.36)		63%	0.85		Very low
Cancer (advanced metastatic)	**Not reported**				-			-	
	overall	8	564	0.22 (0.06 to 0.38)		21%	0.009		NR
	physical functioning	8	564	0.22 (0.05 to 0.38)		24%	0.009		NR

	social functioning	8	564	0.18 (0.02 to 0.34)		0%	0.03		NR
	Emotional function	7	500	0.18 (-0.00 to 0.35)		0%	0.05		NR
	Role function	7	510	0.13 (-0.04 to 0.30)		0%	0.13		NR
	Cognitive function	5	456	0.00 (-0.32 to 0.33)		56%	0.99		NR
Chronic fatigue syndrome	**SF-36**			-				-	
	physical functioning (end of treatment)	5	725		-13.10 (-24.22 to -1.98)	89%	0.02		Low
	physical functioning (52-70 weeks)	3	621		-16.33 (-36.74 to 4.08)	96%	0.12		Very low
Chronic kidney disease	**SF-36**	5	NR	-	-	-	-	2/5 trials favour exercise<br>3/5 neutral	
Chronic liver disease	**EuroQol-VAS**	2	56	-	3.11 (-24.03 to 30.24)	88%	0.82	-	NR
	**SF-36**<br>mental component summary	2	51		2.26 (-3.84 to 8.37)	0%	0.47		NR
Coronary heart disease	**SF-36:**			-				-	
	Physical component score	6	1741		1.70 (-0.08 to 3.47)	73%	0.06		NR
	Mental component score	6	1741		2.14 (1.07 to 3.22)	21%	<0.0001		NR
	Physical functioning	8	2756		8.47 (3.69 to 13.24)	92%	0.0005		NR
	Physical performance	8	2756		8.08 (2.89 to 13.27)	87%	0.002		NR
	Bodily pain	8	2756		-0.06 (-8.97 to 8.84)	97%	0.99		NR
	General health	8	2756		5.66 (2.08 to 9.25)	84%	0.002		NR
	Vitality	7	2638		5.78 (1.89 to 9.67)	85%	0.004		NR
	Social functioning	8	2756		1.98 (0.26 to 3.70)	20%	0.02		NR
	Emotional performance	7	2638		0.69 (-1.33 to 2.71)	18%	0.50		NR
	Mental health	8	2756		5.60 (1.21 to 9.98)	93%	0.01		NR
	**EQ-5D**	3	476		0.05 (-0.01 to 0.10)	69%	0.08		NR
Dementia	Not reported	8	Not reported	-	-	-	-	1/8 trials favour exercise<br>7/8 neutral	Moderate
Depression	**Not reported**				-			-	

	Overall QoL	3	104	0.39 (0.05 to 0.74)		0%	0.002		NR
	Physical domain	5	Not reported	0.53 (0.22 to 0.84)		0%	<0.001		NR
	Psychological domain	5	Not reported	0.53 (0.22 to 0.85)		5%	<0.001		NR
	Social domain	3	Not reported	0.28 (-0.13 to 0.71)		13%	0.18		NR
	Environment domain	3	Not reported	0.36 (-0.12 to 0.85)		31%	0.14		NR
Diabetes mellitus	**Not reported**	1	Not reported	-	-	-	-	1/1 trial neutral	NR
Epilepsy	**SWLS**<br><br>**WHOQOL-BREF**	1<br><br>1	Not reported<br><br>Not reported	-	-	-	-	1/1 trial neutral<br>1/1 trial neutral	NR
Heart failure	all HRQoL measures (SMD)	26	3833	-0.60 (-0.82 to -0.39)	-	87%	<0.0001	-	Low
Inflammatory bowel disease	**SF-36**	1	-	-	-	-	-	1/1 trial favour exercise	NR
Irritable bowel syndrome	**SF-36**	2	-	-	-	-	-	4/5 results favour exercise<br>1/5 neutral	NR
Long-COVID	**SF-12**	1	120	-	-	-	-	1/4 results favour exercise<br>3/4 neutral	NR
Migraine	**Profile of quality of life in the chronically ill**	1	Not reported	-	-	-	-	1/1 trial favour exercise	NR
Osteoporosis	Self-reported functioning	22	1254	-0.64 (-0.89 to -0.40)	-	75%	<0.00001	-	NR
Parkinson's disease	Quality of life (Not reported)<br>Aerobic only	 4	 230	 -0.09 (-0.51 to 0.33)	-	 51%	 0.67	-	NR
Peripheral vascular disease	**SF-36**<br>*3 month*			-				-	
	Physical function	2	82		6.60 (2.37 to 10.83)	74%	0.00		NR
	Bodily pain	2	82		3.89 (-1.91 to 9.68)	0%	0.19		NR
	General health	2	82		4.52 (-0.01 to 9.04)	70%	0.05		NR
	Mental health	2	82		1.44 (-0.93 to 3.81)	0%	0.23		NR
	Role emotional	2	82		1.26 (-4.84 to 7.36)	0%	0.69		NR
	Social function	2	82		1.49 (-4.16 to 7.14)	0%	0.61		NR
	Vitality	2	82		5.55 (1.54 to 9.56)	0%	0.01		NR

	Role physical	2	82		10.31 (3.64 to 16.98)	90%	0.00		NR
	6 month								
	Physical function	2	85		9.78 (0.82 to 18.74)	0%	0.03		NR
	Bodily pain	2	85		4.85 (-3.79 to 13.50)	0%	0.27		NR
	General health	2	85		10.19 (1.83 to 18.55)	0%	0.02		NR
	Mental health	2	85		1.82 (-5.28 to 8.92)	0%	0.62		NR
	Role emotional	2	85		4.90 (-7.15 to 16.94)	0%	0.43		NR
	Social function	2	85		3.48 (-6.74 to 13.71)	77%	0.50		NR
	Vitality	2	85		5.32 (-2.57 to 13.22)	0%	0.19		NR
	Role physical	2	85		8.55 (-2.69 to 19.79)	73%	0.14		NR
	Physical summary score	5	429		2.15 (1.26 to 3.04)	66%	<0.00001		Moderate
	Mental summary score	4	343		3.76 (2.70 to 4.82)	87%	<0.00001		Moderate
Polycystic ovarian syndrome	**SF-36**			-				-	
	Physical functioning	3	84		11.81 (2.36 to 21.25)	74%	0.01		NR
	Role physical	3	84		5.33 (-13.70 to 24.37)	73%	0.58		NR
	Role emotional	3	84		7.46 (-8.78 to 23.69)	56%	0.37		NR
	Bodily pain	3	84		-2.21 (-9.56 to 5.13)	0%	0.56		NR
	General health	3	84		10.05 (3.89 to 16.20)	0%	0.001		NR
	Energy/vitality	3	84		6.43 (-0.82 to 13.67)	5%	0.08		NR
	Social functioning	3	84		11.75 (2.56 to 20.95)	6%	0.01		NR
	Mental health	3	84		11.70 (1.27 to 22.13)	47%	0.03		NR
Psychoactive substance abuse	**SF-36, WHOQOL-BREF**	7	Not reported	-	-	-	-	19/44 results favour exercise 25/44 neutral	NR
Schizophrenia	SMD - QLES, WHOQOL, SF-36, EQ-5D, MANSA				-			-	
	Overall	10	Not reported	0.30 (0.02 to 0.57)		64%	0.03		NR
	Physical	14	Not reported	0.60 (0.32 to 0.87)		74%	<0.001		NR

	Mental	12	Not reported	0.37 (0.13 to 0.61)		65%	0.003		NR
	Social	8	Not reported	0.65 (0.22 to 1.08)		82%	0.003		NR
	Environmental	4	Not reported	0.17 (-0.01 to 0.36)		0%	0.07		NR
Stroke/TIA	**SF-36/SF-12** **Physical health component** Cardiorespiratory training (end of intervention)	2	164	0.51 (-5.02 to -2.13)		0%	0.00		NR
	**SF-36/SF-12** **Mental health component** Cardiorespiratory training (end of intervention)	2	164	0.58 (-0.52 to 1.68)		87%	0.3		NR
	**EQ-5D** Cardiorespiratory training (end of intervention)	2	158		6.55 (-1.36 to 14.47)	48%	0.1		NR
	Cardiorespiratory training (end of FU)	2	150		-4.25 (-8.00 to 1.49)	0%	0.15		NR
	**SF-36 physical functioning** Resistance training (end of intervention)	3	70		5.72 (-5.26 to 16.70)	80%	0.31		NR
	Mixed training (end of intervention)	2	112			0%	0.01		NR
	Mixed training (end of FU)	2	146	0.48 (0.10 to 0.85)	2.46 (-7.20 to 12.11)	57%	0.62		NR
	**SF-36 mental health** Resistance training (end of intervention)	3	70		7.69 (1.56 to 13.83)	38%	0.01		NR
	**SF-36 physical role functioning** Mixed training (end of intervention)	3	178			0%	0.00		NR
	Mixed training (end of FU)	2	146	0.56 (0.26 to 0.86)	11.61 (2.38 to 20.84)	0%	0.01		NR
	**SF-36 social role functioning** Mixed training (end of intervention)	2	112	0.48 (-0.22 to 1.17)		54%	0.18		NR

Treated constipation	**SF-36**, others Not reported	3	NR	-	-	-	-	8/8 results favour exercise	NR
Viral hepatitis	**SF-36**	1	22	-	-	-	-	1/19 results favour exercise 18/19 neutral	NR

*'favours exercise': exercise better than control (P≤0.05); neutral: 'no difference' between exercise and control (P>0.05); 'favours control': control better than exercise (P≤0.05). †GRADE rating as reported in SR
MD: mean difference; SMD: standardised mean difference; CI: confidence interval; NR: not reported; FU: follow-up
EuroQoL = European Quality of Life; EQ-5D = European Quality of Life 5 Dimensions; MANSA = Manchester Short Assessment of Quality of Life; QLES = QoL Enjoyment and Satisfaction scale; SF-36/12 = Short Form 36/12 Health Survey; SWLS = Satisfaction with Life Scale; VAS = visual analogue scale; WHOQOL-BREF = World Health Organization Quality of Life-BREF.

21. Supplementary Figure 6 – Forest plot for generic HRQoL for exercise-based intervention vs control across LTCs – standardized mean difference scale.
* Direction of HRQoL effect reversed so higher follow up outcome score indicates exercise intervention as beneficial.

LTC	Trials	Participants	Standardised mean difference (95% CI)	I2	p-value
Arthritis (osteo-, hip)	3	183	0.10 (-0.23, 0.36)	0	.66
Arthritis (osteo-, knee)	13	1073	0.28 (0.15, 0.40)	0	<0.00001
Atrial fibrillation	8	632	0.63 (0.18, 1.09)	85.6	<0.001
Cancer (haem)	8	1259	0.11 (-0.03, 0.24)	26	.14
Cancer (advanced mets)	8	564	0.22 (0.06, 0.38)	21	.009
Depression	3	104	0.39 (0.05, 0.74)	0	.002
Heart failure*	26	3833	0.60 (0.39, 0.82)	87	<0.0001
Osteoporosis*	22	1254	0.64 (0.40, 0.89)	75	<0.00001
Parkinson's disease*	4	230	0.09 (-0.33, 0.51)	51	.67
Schizophrenia	10	NR	0.30 (0.02, 0.57)	64	.03
Stroke/TIA	3	178	0.56 (0.26, 0.86)	0	0

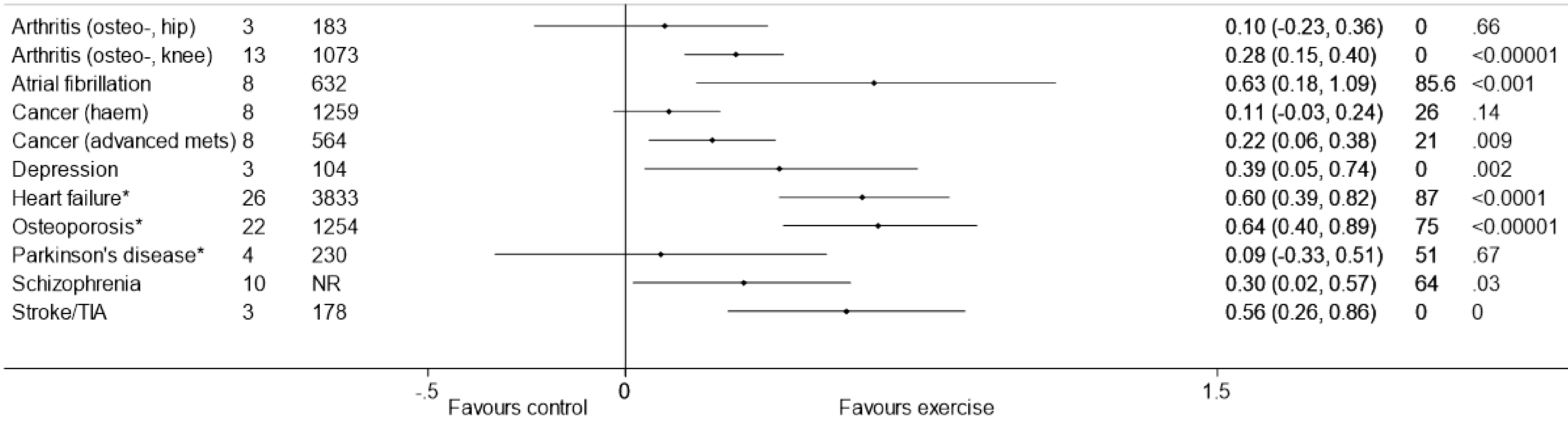

22. Supplementary Figure 7 – Forest plot for HRQoL for exercise-based intervention vs control measured using three different SF-36 domains.

Results are presented as mean difference on 0-100 scale. Panel A shows the physical function subscale, panel B the role physical subscale, and panel C the mental health subscale. * Direction of HRQoL effect reversed so higher follow up outcome score indicates exercise intervention as beneficial.

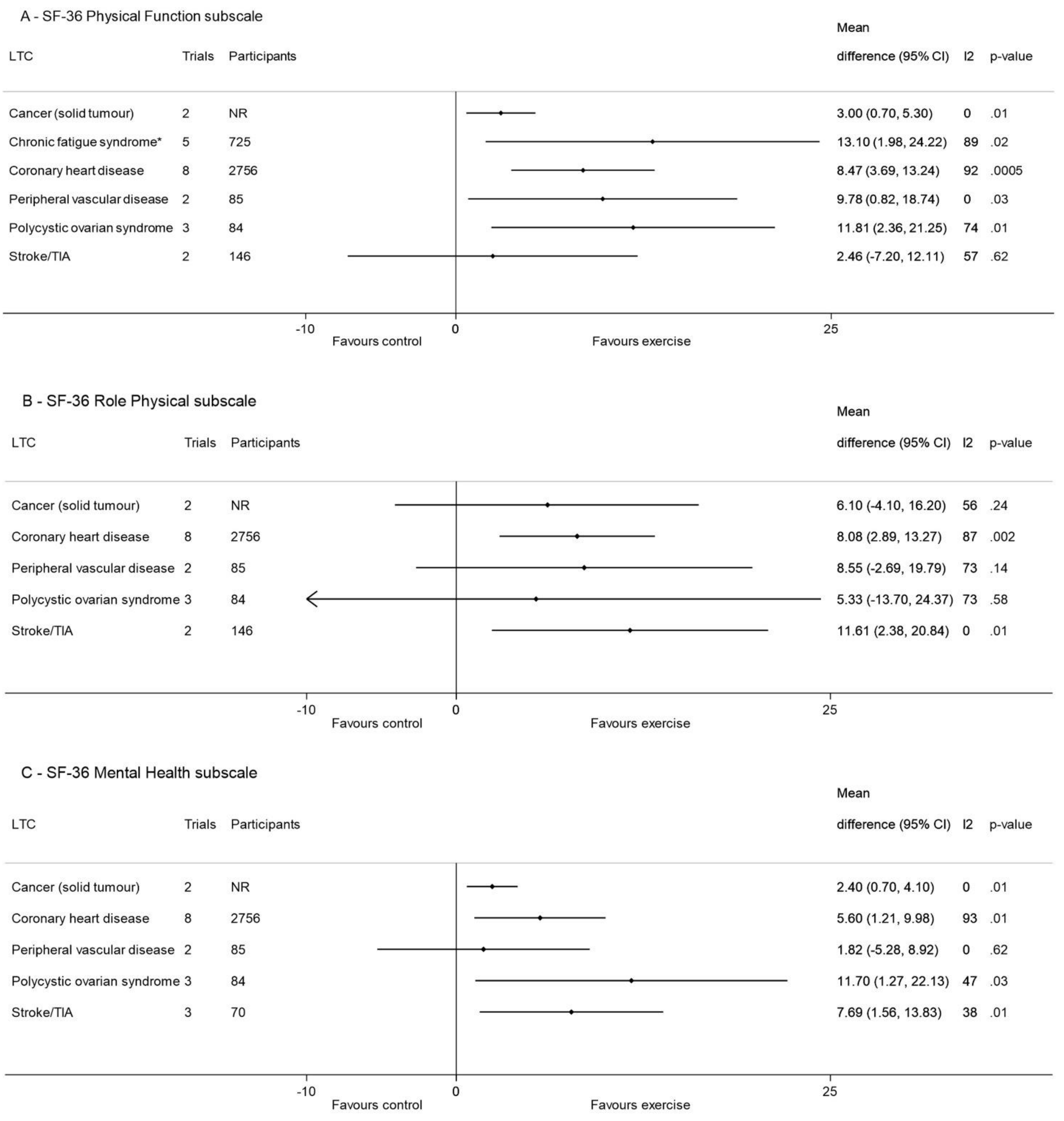

23. Supplementary Table 13 – Summary of exercise-based intervention vs control for physical activity outcomes by LTC

LTC	Measurement method	N trials/ comparisons in MA	N participants in MA	Effect SMD (95% CI)	I^2	P-value	Vote counting
Dementia	Not reported	5	NR	-	-	-	1/4 results favour exercise<br>3/4 neutral
Long COVID	Steps/day (mobile app)	1	196	-	-	-	1/1 trials favour exercise
Psychoactive substance abuse	Moderate and Vigorous physical activity (days, mins; IPAQ)	1	NR	-	-	-	3/4 results favour exercise<br>1/4 neutral
Schizophrenia	Self-reported physical activity	4	NR	0.04 (-0.22 to 0.29)	35%	0.204	-
Stroke/TIA	Diary & accelerometer (total, light, moderate, vigorous at end of intervention or end of follow up)	1	NR	-	-	-	8/8 results neutral

*'favours exercise': exercise better than control (P≤0.05); neutral: 'no difference' between exercise and control (P>0.05); 'favours control': control better than exercise (P≤0.05). NR: not reported in SR; IPAQ: International Physical Activity Questionnaire
